# Co-catalyzed arylation of aldehydes and aryltrimethylgermanes†

**DOI:** 10.1039/d3ra00836c

**Published:** 2023-03-10

**Authors:** Qiang Zhang, Xiao Zou, Ningqi Zhang, Bo Liu

**Affiliations:** a Shaanxi Key Laboratory of Catalysis, School of Chemistry and Environmental Science, Shaanxi University of Technology Han zhong 723001 P. R. China zhangqiang22@126.com

## Abstract

A novel cobalt-catalyzed protocol for the synthesis of carbinol derivatives and benzil derivatives has been developed. In the presence of CoI_2_ as the catalyst and tmphen (3,4,7,8-tetramethyl-1,10-phenanthroline) as the ligand, the corresponding arylated products were obtained from the addition of aryltrimethylgermanes to aromatic aldehydes and arylglyoxals in moderate to excellent yields under air atmosphere.

## Abstract 2

A novel cobalt-catalyzed protocol for the synthesis of carbinol derivatives and benzil derivatives has been developed. In the presence of CoI_2_/tmphen as the catalyst, the corresponding arylated products were obtained in moderate to excellent yields under air atmosphere.

## Graphical abstract

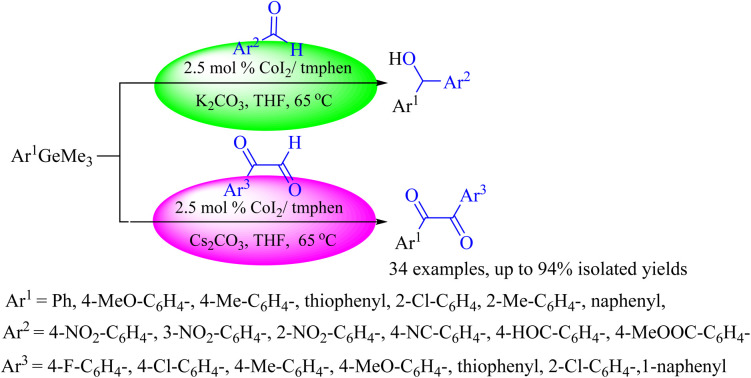

## 1. Introduction

In the past decade, transition metal catalysis has been recognized as a powerful synthetic tool for diarylmethanols through the addition of organometallic reagents.^1,2^ Organogermanes^3^ have received much less attention so far, compared with their congeners such as organosilanes^4^ and organostannanes due to their lower reactivity, the higher cost of germanium relative to silicon^5^ and the less reported synthetic methodology of organogermanes.^5–9^ To the best of our knowledge, organogermanes are more susceptible to breaking the C–Ge bond than arylsilane analogues,^9^ and have lower carbon–metal bond energy and a larger covalent radius than their silicon counterparts in group IVA. However, examples of employing aryltrimethylgermanes in addition reactions have been never reported before. Our previous work^10^ prompted us to explore the possibility of employing low-cost catalysts in addition reactions. Herein, we report our preliminary results on the first example of cobalt-catalyzed addition of aromatic aldehydes and arylglyoxals with ArGeMe_3_ using a CoI_2_/tmphen catalytic system.

**Fig. 1:**
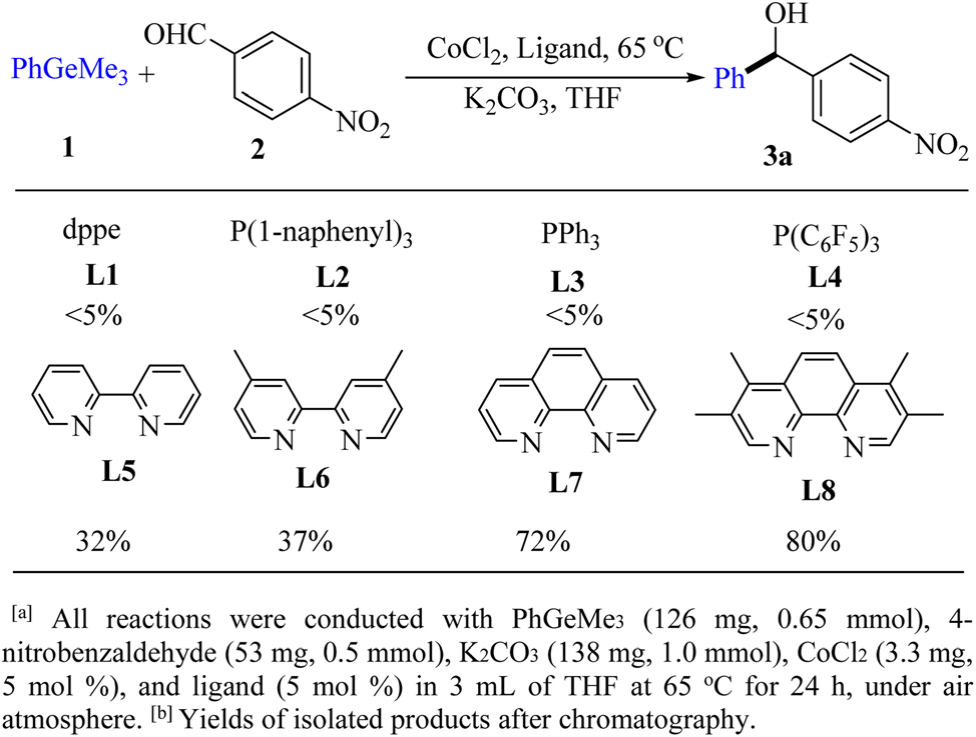
Ligand screening.^*a*^

The reaction of PhGeMe_3_ (1a) and 4-nitrobenzaldehyde (2a) was firstly chosen as the model reaction for this cobalt-catalyst system (Fig. 1).

Ligands were firstly screened since it often plays an important role in transition-metal-catalyzed chemistry.^9^ The effects of phosphine ligand with different electron-donating, electron-withdrawing and steric hindrance groups were examined (Fig. 1, L1–L4), but no target product was detected. However, the yield of 3a could be improved to 80% when the combination of CoCl_2_ and tmphen (L8) was employed (Fig. 1, L8). Subsequently, various reaction conditions concerning the types and amount of cobalt sources, the effects of time and temperature, bases, solvents, were examined to increase the yield of product (Table 1). After extensive screening, the optimized reaction condition was established as follows: CoI_2_ (2.5 mol%), tmphen (L8, 2.5 mol%), K_2_CO_3_ (1.0 mmol), THF (3.0 mL), ArGeMe_3_ (0.65 mmol) and aldehydes (0.5 mmol), 65 °C, 12 h. Among the bases we used, K_2_CO_3_ was superior to other bases such as NaHCO_3_, Na_2_CO_3_, NaOAc, KF, Li_2_CO_3_, and Cs_2_CO_3_. 14% yield of benzophenone was detected when using Cs_2_CO_3_ as the base under model reaction condition (Table 1, entry 7). The choice of solvents was also crucial to the reaction. THF was proved to be the best one of all the solvents we chosed.

**Table 1:** Selected results for the optimal reaction conditions^a^

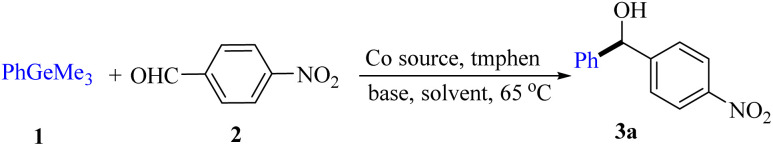				
Entry	Catalyst	Base	Solvent	Yield^b^ (%)
1	—	—	THF	N.R
2	CoCl_2_	—	THF	N.R
3	CoCl_2_	NaHCO_3_	THF	21
4	CoCl_2_	Na_2_CO_3_	THF	51
5	CoCl_2_	NaOAc	THF	28
6	CoCl_2_	KF	THF	53
7	CoCl_2_	Cs_2_CO_3_	THF	71
8	CoCl_2_	K_2_CO_3_	THF	80
9	CoCl_2_	K_2_CO_3_	DME	62
10	CoCl_2_	K_2_CO_3_	CH_3_CN	<5
11	CoCl_2_	K_2_CO_3_	DMF	<5
12	CoCl_2_	K_2_CO_3_	Dioxane	37
13^c^	CoI_2_	K_2_CO_3_	THF	92
14^d^	CoI_2_	K_2_CO_3_	THF	90
15	CoI_2_	K_2_CO_3_	THF	87
16	CoBr_2_	K_2_CO_3_	THF	67
17	Co(OAc)_2_	K_2_CO_3_	THF	58
18	Co(C_5_H_5_)_2_	K_2_CO_3_	THF	14
19	Co_3_O_4_	K_2_CO_3_	THF	<5
20	PdCl_2_	K_2_CO_3_	THF	<5
21	RhCl_3_·3H_2_O	K_2_CO_3_	THF	<5

a Reaction conditions: 1 (126 mg, 0.65 mmol), 2 (76 mg, 0.5 mmol), cobalt source (5 mol%), tmphen (L8, 5.9 mg, 5 mol%); base (1.0 mmol), solvent (3 mL), 65 °C for 12 h, under air in reaction tubes.

b Yields of isolated products after chromatography.

c CoI_2_ (3.9 mg, 2.5 mol%), tmphen (L8, 3.0 mg, 2.5 mol%).

d CoI_2_ (15.6 mg, 10 mol%), tmphen (L8, 11.8 mg, 10 mol%).

Subsequently, various reaction conditions concerning the types and amounts of cobalt sources, the effects of time and temperature, bases, solvents, were examined to increase the yield (Table 1). After extensive screening, the optimized reaction condition was established as follows: CoI_2_ (2.5 mol%), tmphen (L8, 2.5 mol%), K_2_CO_3_ (1.0 mmol), THF (3.0 mL), ArGeMe_3_ (0.65 mmol) and aldehydes (0.5 mmol), 65 °C, 12 h. Among the bases we used, K_2_CO_3_ was superior to other bases such as NaHCO_3_, Na_2_CO_3_, NaOAc, KF, Li_2_CO_3_, and Cs_2_CO_3_. 14% yield of benzophenone was detected when using Cs_2_CO_3_ as the base under model reaction condition (Table 1, entry 7). The choice of solvents was also crucial to the reaction. THF was proved to be the best one of all the solvents we chosed.

With the optimized conditions in hand, a variety of aldehydes with electron-rich, electron-deficient and steric hindrance was examined to broaden the extent of the reaction. Typical functional groups such as methyl, methoxyl, fiuoro, chloro were well tolerated under the reaction conditions. Electron-deficient analogues of aldehyde reacted with ArGeMe_3_ easily and gave biarylmethanols in good yields (Table 2, entries 1–10). Particularly, 4-formylbenzaldehyde could react with PhGeMe_3_ and the product of 3n and keep one formyl group untouched (Table 2, entry 14). The chloro and bromo groups untouched in this catalytic system (Table 2, entries 9 and 10). Unfortunately, the reaction was stopped by using aldehydes with neutral and electron-rich groups or aliphatic aldehydes due to its low activity to aryltrimethylgermane under this reaction condition. However, butyraldehyde or 4-methoxybenzaldehyde as substrate react with phenyltrimethylgermane did not give the responding products. Similarly, tetramethylgermane as substrate react with 4-nitro-phenyladehyde also did not give the responding products.

**Table 2:** Selected results for the optimal reaction conditions^a^

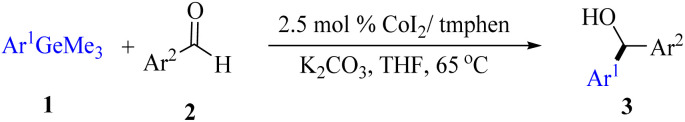		
Entry	Product	Yield^b^ (%)
1	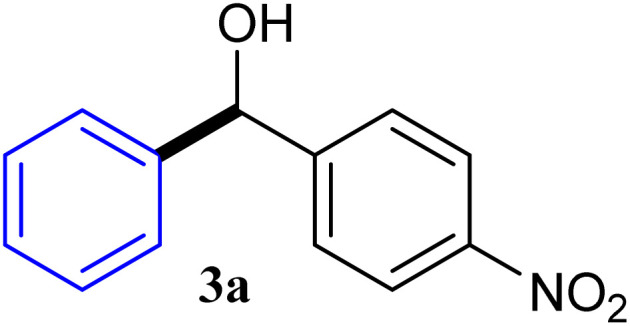	92
2	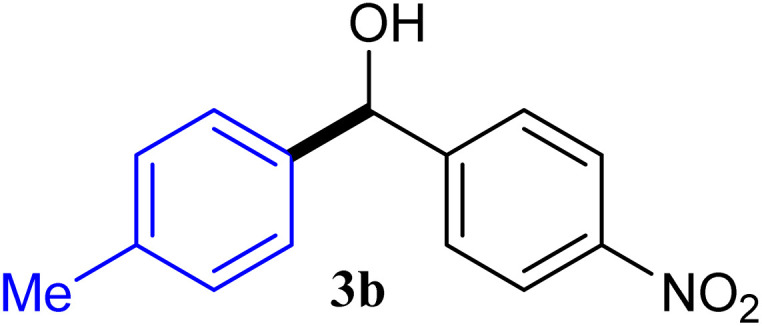	94
3	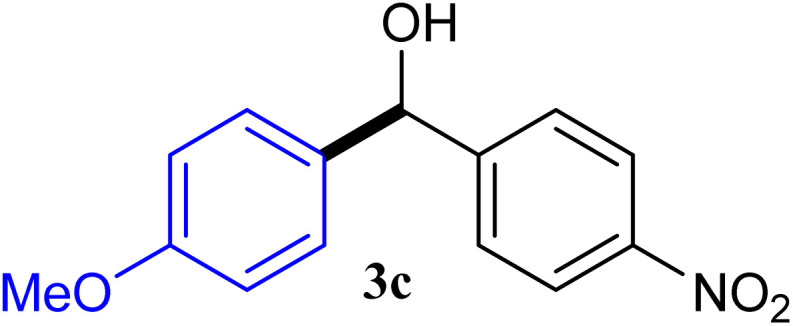	93
4	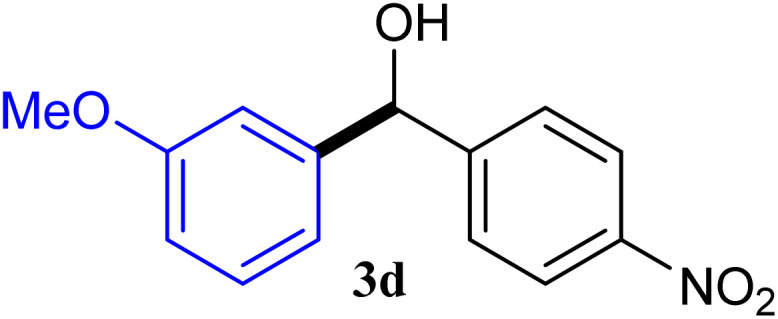	93
5	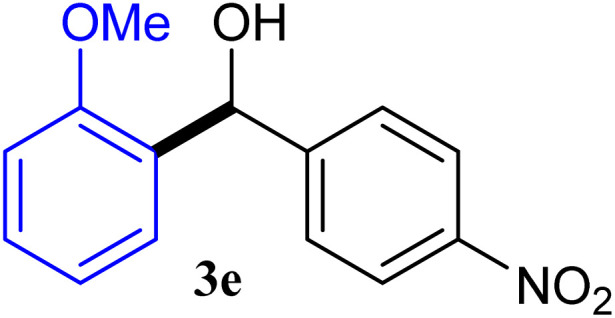	91
6	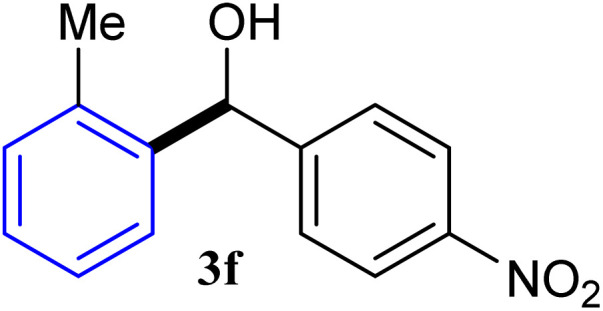	92
7	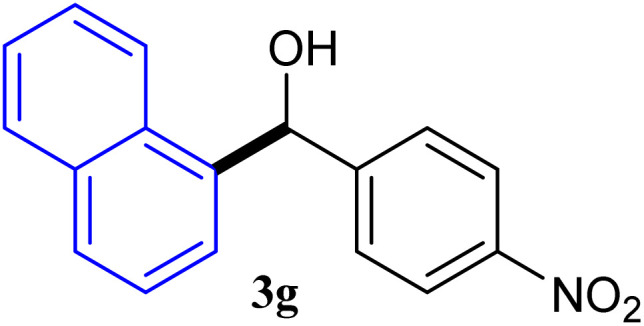	93
8	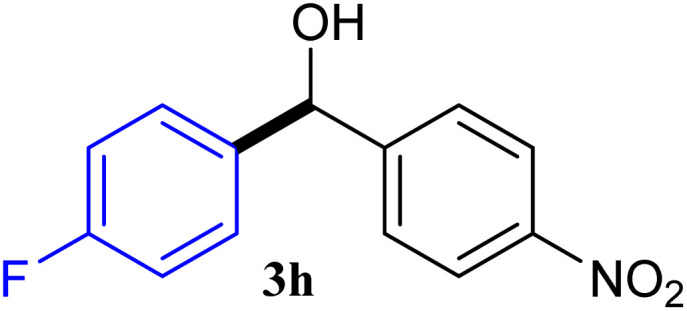	95
9	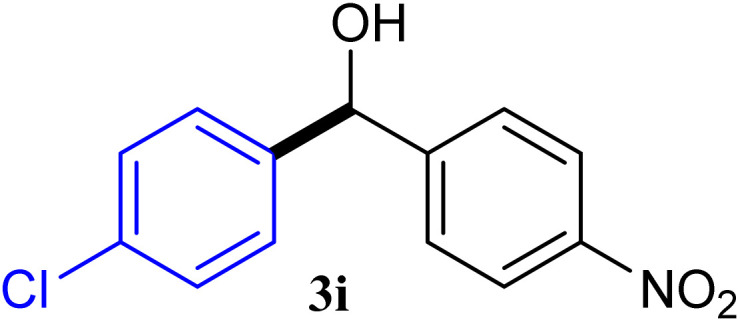	92
10	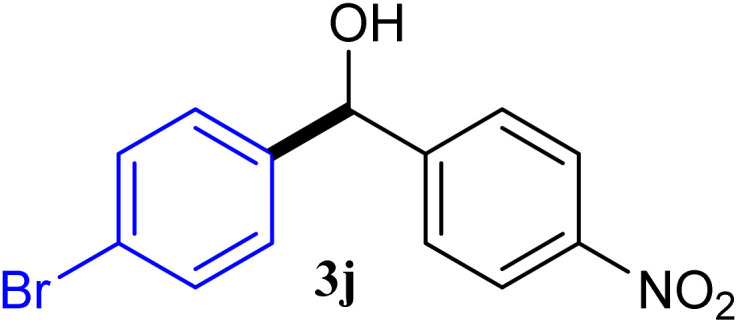	91
11	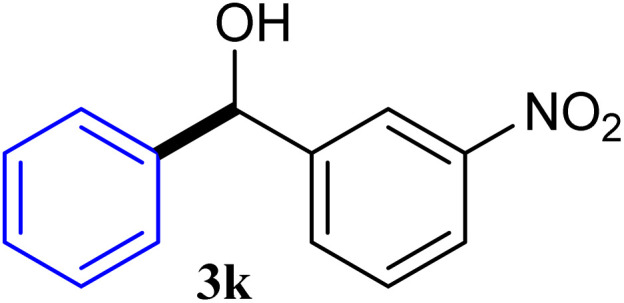	85
12	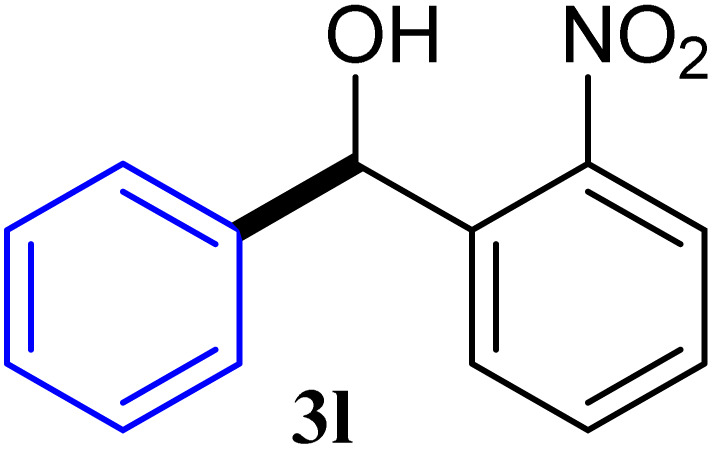	76
13	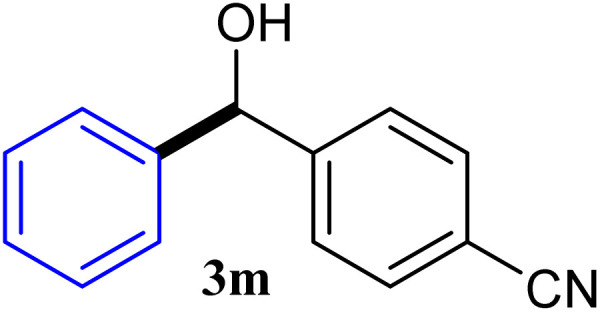	87
14	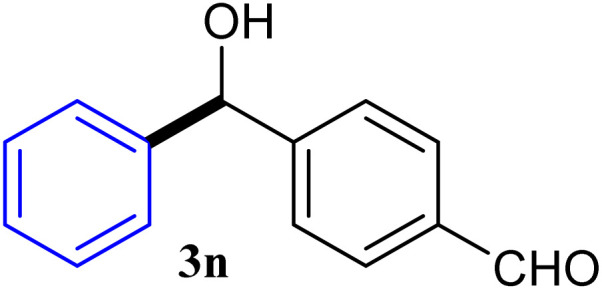	93
15	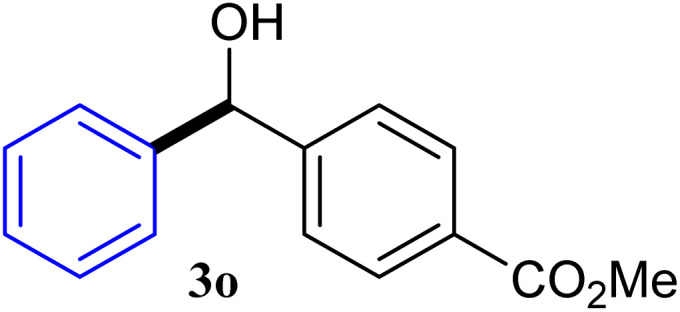	55
16	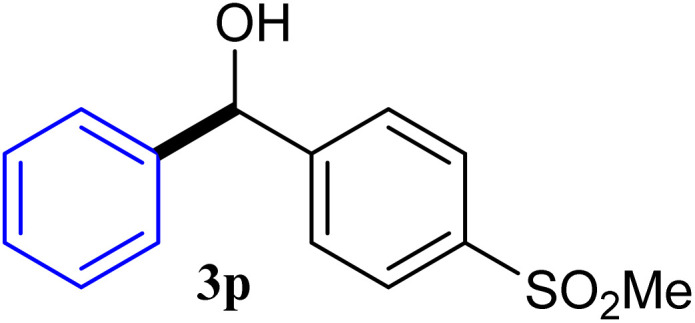	91

a Reaction conditions: ArGeMe_3_1 (0.65 mmol), aldehyde 2 (0.5 mmol), CoI_2_ (3.9 mg, 2.5 mol%), tmphen (L8, 3.0 mg, 2.5 mol%), K_2_CO_3_ (138 mg, 1.0 mmol), THF (3 mL), 65 °C for 12 h, under air in pressure tubes.

b Yields of isolated products after chromatography.

During broadening the extent of the reaction, the phenylglyoxal hydrate was examined to broaden the scope of the reaction, which could be seen as the electron-deficient analogue instead of 4-nitrobenzaldehyde. Only a trace of benzoin formed, the benzil was instead the major final product. Obviously, benzil was resulted from catalytic oxidation of *in situ* generated benzoin in the presence of K_2_CO_3_ in air. It was noteworthy that the over oxidation product could not be detected under argon atmosphere with degassed THF. A more efficient catalytic system with the dual ability to facilitate the addition of ArGeMe_3_ to phenylglyoxal hydrate was obtained when K_2_CO_3_ was exchange by Cs_2_CO_3_ as the base. Then, the optimized reaction conditions were then extended to conversions of PhGeMe_3_ to phenylglyoxal hydrate as follows: CoI_2_ (3.9 mg, 2.5 mol%), tmphen (L8, 3.0 mg, 2.5 mol%), Cs_2_CO_3_ (326 mg, 1.0 mmol), THF (3.0 mL), PhGeMe_3_(0.65 mmol) and phenylglyoxal hydrate (0.5 mmol), 65 °C, 12 h (Table 3).

**Table 3:** Selected results for the optimal reaction conditions^a^

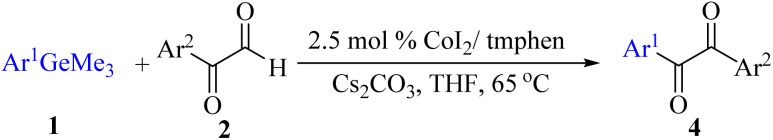		
Entry	Product	Yield^b^ (%)
1	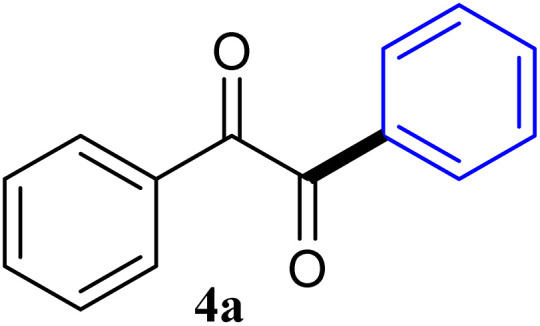	95
2	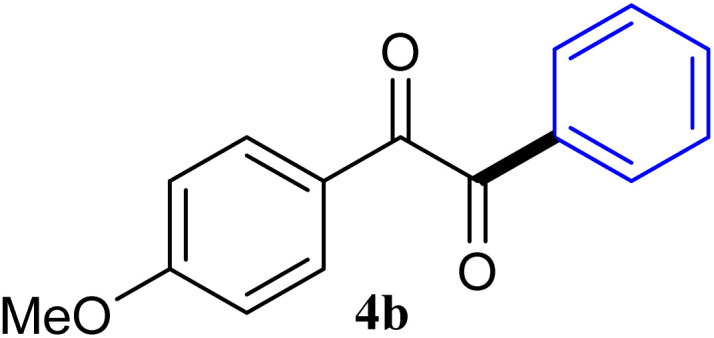	94
3	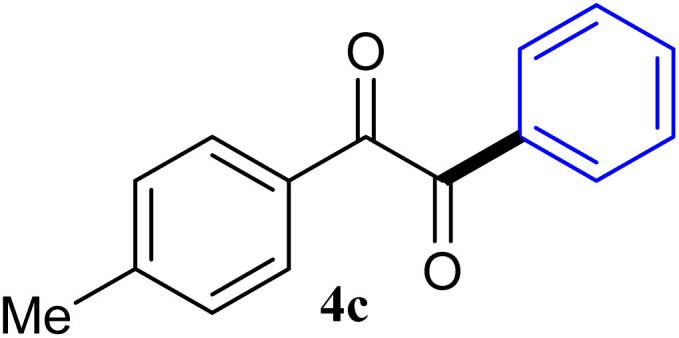	93
4	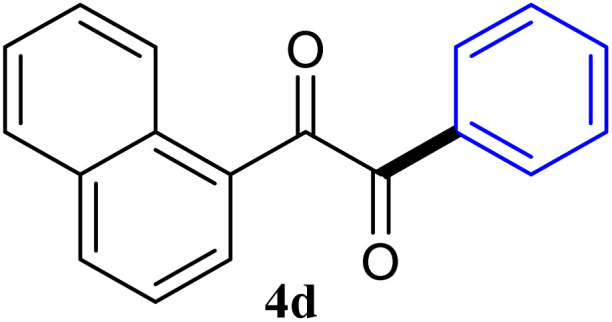	92
5	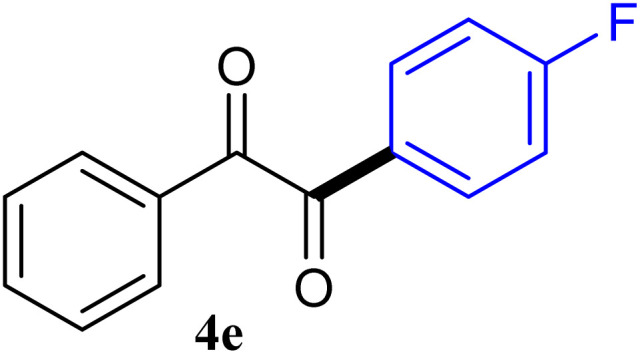	91
6	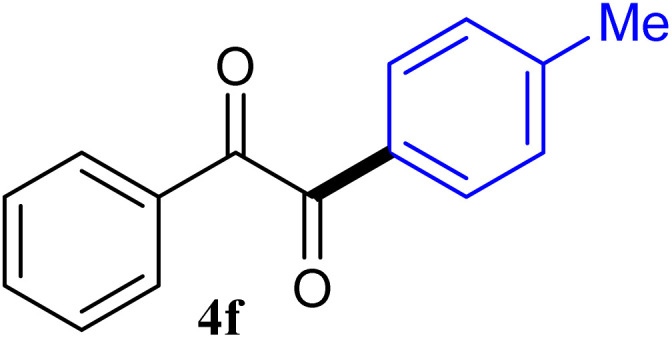	93
7	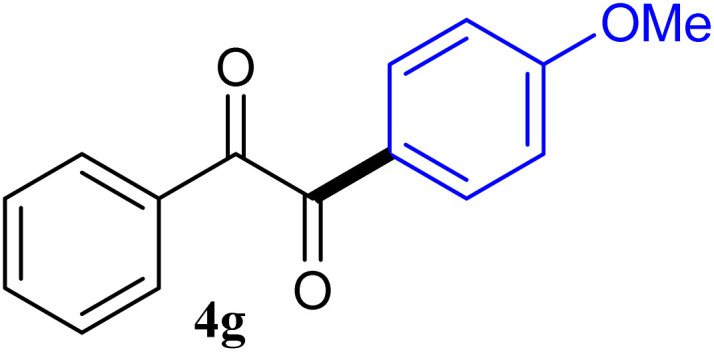	92
8	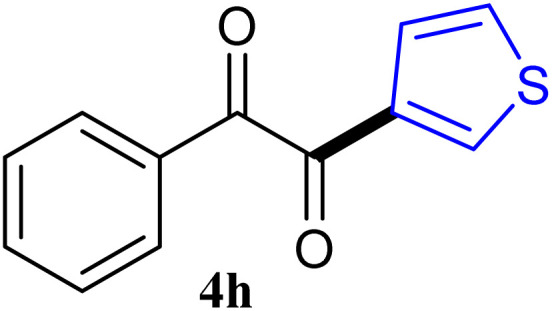	67
9	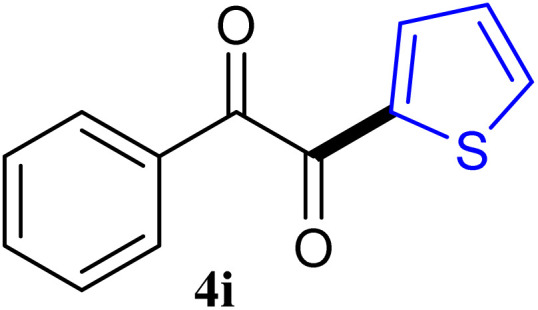	59
10	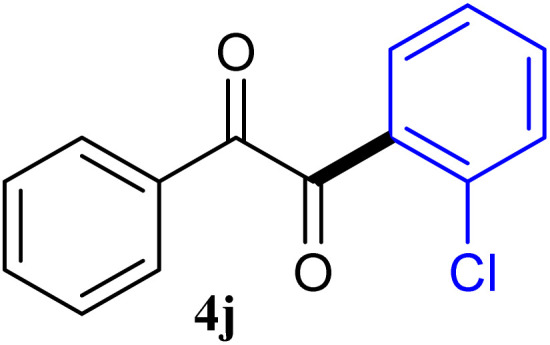	78
11	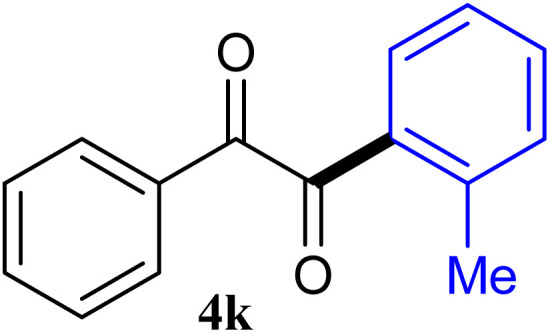	87
12	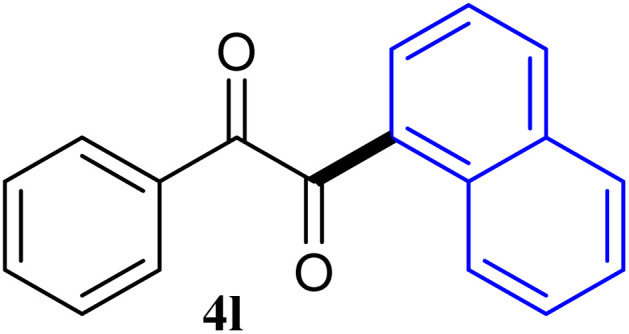	91
13	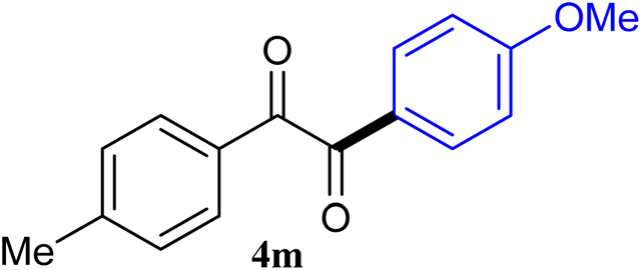	92
14	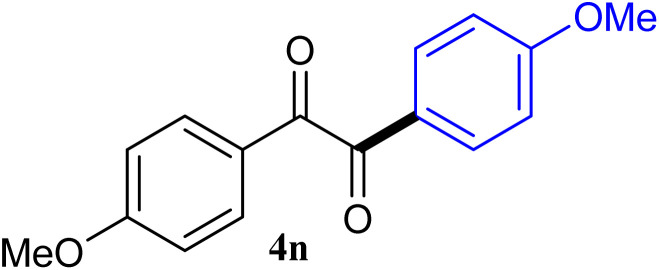	94
15	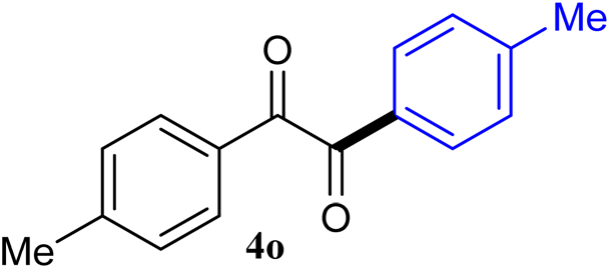	93

a Reaction conditions: ArGeMe_3_ (0.65 mmol), arylglyoxal (0.5 mmol), CoI_2_ (3.9 mg, 2.5 mol%), tmphen (L8, 3.0 mg, 2.5 mol%), Cs_2_CO_3_ (138 mg, 1.0 mmol), THF (3 mL), 65 °C for 12 h, under air in pressure tubes.

b Yields of isolated products after chromatography.

The reactions of different ArGeMe_3_ with arylglyoxals were examined to broaden the scope of the reaction. All the reactions catalyzed by CoI_2_/tmphen proceeded well and provided the desired products in good yields. Although the hetero-atoms in the heteroaryltrimethylgermanes might coordinate to transition-metal, trimethyl(thiophen-3-yl)germane and trimethyl(thiophen-2-yl)germane were still good partners for the addition reaction. The corresponding products were isolated in 67% and 59% yields, respectively (Table 3, entries 11 and 12). It seemed that the ortho substituents had little influence on their activities. For instance, (2-chlorophenyl)trimethylgermane, trimethyl(*o*-tolyl)germane and trimethyl(naphthalen-1-yl)germane could react with phenylglyoxal hydrate to furnish 4j, 4k, and 4l in excellent yields (Table 3, entries 13–15). The comparison of PhGeMe_3_ and its congener PhSiMe_3_ was also investigated under the optimised reaction conditions. However, PhSiMe_3_ was not the proper candidates and recovered the reactants. Similarly, tetramethylgermane as substrate to react phenylglyoxal hydrate did not give the responding products.

**Fig. 2:**
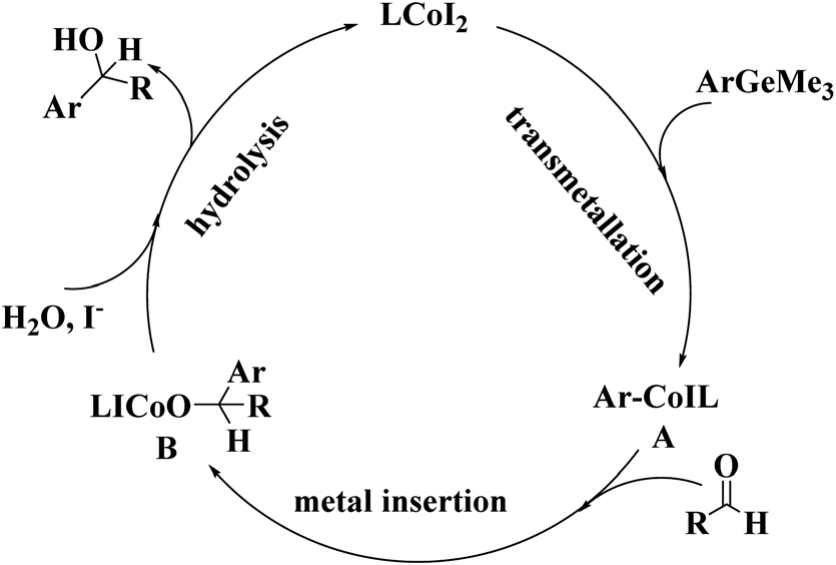
A plausible mechanism.

To further understand the mechanism, the model reaction under optimized reaction conditions was studied by gas chromatography-mass spectrometry. The data showed that 1,1′-biphenyl and hexamethyldigermane were the by-products, except for the addition product and the reactants. To account for the present reaction, a plausible mechanism based upon the above experimental results was proposed as follows (Fig. 2).

A plausible mechanism for forming diarylmethanols (Fig. 2): the catalytic cycle may contain three steps: Co(ii) undergoes transmetallation to form ArCo(ii)IL (**A**), which exhibits high nucleophilicity toward carbonyl carbon might produce the byproduct with 1,1′-diphenyl and hexamethyldigermane through cross-coupling reaction. Then arylcobalt^11^ should be transferred to carbonyl carbon through the insertion gives the intermediate (B). Finally, the hydrolysis of intermediate (B) affords the diarylmethanols. Cs_2_CO_3_ might facilitate the addition of aryltrimethylgermane to arylglyoxal and prompt the aerobic oxidation of the carbinol; The ICo–OH species reacts with I^−^ to regenerate the active CoI_2_ for the next cycle.

In summary, we describe here the first time a mild cobalt-catalyzed nucleophilic arylation of aromatic aldehydes and arylglyoxals with ArGeMe_3_ using CoI_2_/tmphen catalytic system. In the presence of CoI_2_/tmphen catalytic system, a variety of electron-deficient arylaldehydes and arylglyoxals was found to be suitable substrates for the reaction with ArGeMe_3_ in moderate to excellent yields. It was noteworthy that our methodology could keep the formyl group chloro and bromo groups untouched for further functionalization. This method might provide potential opportunities for the addition of ArGeMe_3_ to unsaturated carbon–carbon bonds and unsaturated carbon-hetero bonds. The detailed mechanism of the reaction and further applications of ArGeMe_3_ are the focus of ongoing efforts in our laboratory.

## Conflicts of interest

There are no conflicts to declare.

## Supplementary Material

RA-013-D3RA00836C-s001
