# Supplementary material for: Co-catalyzed arylation of aldehydes and aryltrimethylgermanes
Source: RSC Adv. 2023 Mar 10;13(12):8043–8. doi: 10.1039/d3ra00836c (PMC9999368; doi:10.1039/d3ra00836c)
Supplement: RA-013-D3RA00836C-s001 [file RA-013-D3RA00836C-s001.pdf]

Electronic Supplementary Material (ESI) for RSC Advances.
This journal is © The Royal Society of Chemistry 2023

Electronic Supplementary Material (ESI) for RSC Advances.

This journal is © The Royal Society of Chemistry 2023

Co-catalyzed Arylation of Aldehydes and Aryltrimethylgermanes

Qiang Zhang,* Xiao Zou, Ningqi Zhang, Bo Liu

Shaanxi Key Laboratory of Catalysis, School of Chemistry and Environmental Science, Shaanxi University of Technology, Han zhong, 723001, P. R. China

E-mail: zhangqiang22@126.com

1. Experimental reagents and instruction 1

2. General experimental procedures 2

3. Experimental characterization data for diarylmethanols 3

4. Experimental characterization data for benzil derivatives 10

5. The NMR spectra of diarylmethanols 16

6. The NMR spectra of benzil derivatives 33

1. Experimental reagents and instruction

Chemicals were either purchased or purified by standard techniques without special instructions. The reactions were monitored using analytical thin layer chromatography (TLC, GF-254). Flash chromatography was performed using silica gel (300-400 mesh) with freshly distilled solvents. The boiling range of petroleum ether in this research is 60-90 ºC. Arylglyoxals[1] and were prepared according to the reported procedures. ^{1}H NMR and ^{13}C NMR spectra were measured on a Bruker spectrometer, using $CDCl_3$ as the solvent with tetramethylsilane (TMS) as an internal standard at room temperature. The mass spectra of the starting materials and the intermediates were recorded on a Bruker Avance Mass Spectrometer (maXis, ESI). Chemical shifts are given in δ relative to TMS, the coupling constants J are given in Hz.

[1] a) M. B. Floyd, M. T. Du, P. F. Fabio, L. A. Jacob, B. D. Johnson, *J. Org. Chem.*, **1985**, *50*, 5022–5027; (b) B. Khalili, P. Jajarmi, B. Eftekhari-Sis, M. M. Hashemi, *J. Org. Chem.* **2008**, *73*, 2090–2095.

2. General experimental procedures

Aryltrimethylgermanes were synthesized from the corresponding arylbromides and chlorotrimethylgermane according to the literature procedure [2] with a slight modification. Trimethyl(phenyl)germane. To a 500 mL three necked flask were fitted with a magnetic stirrer, a thermometer, and a pressure equalizing dropping funnel. The flask was charged with bromobenzene (15.7 g, 100 mmol), and dry THF (200 mL) under Ar atomosphere. The reaction mixture was cooled to *ca.* – 80 ºC and *n*-BuLi/hexane (2.5 M, 52 mL, 130 mmol) was added dropwise over 1.5 h. Upon completion of the addition, the reaction mixture was stirred at –80 ºC for 3 h, chlorotrimethylgermane (18.4, 120 mmol) was added dropwise and then allowed to warm to room temperature. The resulting mixture was acidified with 2 N HCl (26 mL) and stirred for overnight. After being concentrated to 1/3 volume, the mixture was poured into water, extracted with diethyl ether and washed with water, dried over $MgSO_4$, followed by filtration under rotary evaporation, and the residue was purified by column chromatography (silica gel, petroleum ether / ethyl acetate, v/v) to give a colorless liquid (22.3 g, yield 86 %) (17.3 g, yield 89 %). The product[2c] was used without further purification in the next step.

(1) The best reaction condition of the synthesis of diarylmethanols

A 10 mL pressure tubes was charged with CoI_2 (3.9 mg, 2.5 mol %), **tmphen** (**L8**，3.0 mg, 2.5 mol %), K_2CO_3 (1. 0 mmol), aldehyde (0. 5 mmol), aryltrimethylgermane(0.65 mmol), and THF (2 mL) into the reaction tube stirred for 10 min at room temperature. Then, the mixture was heated at 65 ºC for 12 h. After completion of the reaction, as indicated by TLC, the reaction mixture was extracted with ethyl acetate (3×10 mL), concentrated and purified by flash column on a silica gel (silica gel 200-300 mesh), petroleum ether/ethyl acetate as the eluent, to give the product. The identity and purity of the product was confirmed by ^{1}H NMR and ^{13}C NMR spectroscopic analysis.

(2) The best reaction condition of the synthesis of benzil derivatives

A 10 mL pressure tubes was charged with CoI_2 (3.9 mg, 2.5 mol %), **tmphen** (**L8**，3.0 mg, 2.5 mol %), Cs_2CO_3 (1.0 mmol), arylglyoxal (0.5 mmol), aryltrimethylgermane(0.65 mmol), and THF (2 mL) into the reaction tube stirred for 10 min at room temperature. Then, the mixture was heated at 65 ºC for 12 h. After completion of the reaction, as indicated by TLC, the reaction mixture was

---

[2] a) S. M. Moerlein, *J. Org. Chem.* **1987**, *52*, 664–667; b) Z. T. Zhang, J. P. Pitteloud, L. Cabrera, Y. Liang, M. Toribio, S. F. Wnuk, *Org. Lett.* **2010**, *12*, 816–819; b) N. Komami, K. Matsuoka, A. N. M. Kojima, T. Yoshino, S. Matsunaga, *Chemistry A Eurppean Journal,* **2019**, 24, 1217-1220.

extracted with ethyl acetate (3×10 mL), concentrated and purified by flash column on a silica gel(silica gel 200-300 mesh), petroleum ether/ethyl acetate as the eluent, to give the product. The identity and purity of the product was confirmed by ^{1}H NMR and ^{13}C NMR spectroscopic analysis.

3. Experimental characterization data for diarylmethanols

4-nitrophenyl(phenyl)methanol[3] (**3a**)

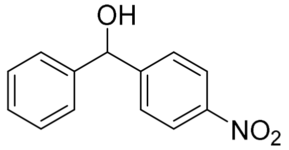

(4-nitrophenyl)(phenyl)methanol (**3a**). The product was isolated as a light-yellow solid (105 mg, 92%) after column chromatography purification using a solution of petroleum ether and ethyl acetate 5:1(*v/v*) as the eluent. mp 52–53 °C; IR (neat, cm^{-1}): 1350(NO_2), 1540(NO_2), 3340(OH); The NMR spectra of 4-nitrophenyl(phenyl)methanol are shown as Attached Fig.1 and Attached Fig.2 in supplementary material, respectively.^{1}H NMR ($CDCl_3$, 600 MHz) δ 2.48 (brs, 1H), 5.91 (s, 1H), 7.30-7.35 (m, 5H), 7.57 (d, J = 12.0 Hz, 2H), 8.18 (d, J = 12.0 Hz, 2H); ^{13}C NMR ($CDCl_3$, 150 MHz) δ 123.7, 126.7, 127.0, 127.0, 128.4, 128.9, 142.6, 147.1, 150.6; HRMS (ESI) m/z calcd for ($C_{13}H_{11}NO_3$+Na) 252.0637, found 252.0651. ($C_{13}H_{11}NO_3$+Na).

(4-nitrophenyl)(4-tolyl)methanol[4] (**3b**)

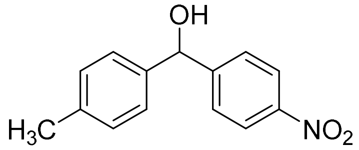

4-nitrophenyl-4-tolylmethanol (**3b**). The product was isolated as a light-yellow solid (114 mg, 94%) after column chromatography purification using a solution of petroleum ether and ethyl acetate 6:1(*v/v*) as the eluent. mp 98–100 °C; IR (neat cm^{-1}): 1350(NO_2), 1540(NO_2), 2870(CH_3), 2960(CH_3), 3341(OH); The NMR spectra of 4-nitrophenyl-4-tolylmethanol are shown as Attached Fig.3 and Attached Fig.4 in supplementary material, respectively.^{1}H NMR ($CDCl_3$, 300 MHz) δ 2.32 (d, J = 3.0 Hz, 1H), 2.36 (s, 3H), 5.90 (d, J = 3.0 Hz, 1H), 7.16-7.26 (m, 4 H), 7.59 (d, J = 6.9 Hz, 2H), 8.19 (d, J = 6.9 Hz, 2H); ^{13}C NMR ($CDCl_3$, 75 MHz) δ 21.0, 75.3, 123.5, 126.6, 126.9, 129.5, 138.2, 139.9, 147.0, 150.9;; HRMS (ESI) m/z calcd for ($C_{14}H_{13}NO_3$+Na) 266.0793, found 266.0784. ($C_{14}H_{13}NO_3$+Na).

[3] C. M. Qin, H. Y. Wu, J. Cheng, J. X. Chen, M. C. Liu, W. W. Zhang, W. K. Su, J. C. Ding, *J. Org. Chem.* **2007**, *72*, 4102–667.
[4] S. H. Lin, X. Y. Lu, *J. Org. Chem.* 2007, 72, 9757–9761.

(4-methoxyphenyl)(4-nitrophenyl)methanol[4] (**3c**)

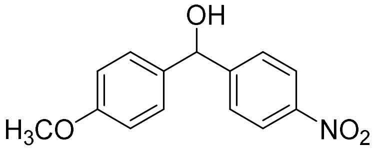

(4-methoxyphenyl)(4-nitrophenyl)methanol (**3c**). The product was isolated as a light-yellow solid (120 mg, 93%) after column chromatography purification using a solution of petroleum ether and ethyl acetate 6:1(*v/v*) as the eluent. mp 54–56 °C; IR (neat cm^{-1}): 1350(NO_2), 1540(NO_2), 2850(OCH_3), 2930(OCH_3), 3340 (OH); The NMR spectra of (4-methoxyphenyl)(4-nitrophenyl) methanol are shown as Attached Fig.5 and Attached Fig.6 in supplementary material, respectively. ^{1}H NMR ($CDCl_3$, 300 MHz) δ 2.74 (brs, 1H), 3.79 (s, 3H), 5.86 (s, 1H), 6.82-6.93(m, 4H), 7.25-7.30 (m, 3H), 7.58 (d, J = 8.6 Hz, 2H), 8.18 (d, J = 8.6 Hz, 2H); ^{13}C NMR ($CDCl_3$, 75 MHz) δ 55.2, 75.3, 112.4, 113.4, 118.9, 127.0, 128.2, 130.9, 144.0, 147.1, 151.0, 159.9; HRMS (ESI) m/z calcd for ($C_{14}H_{13}NO_4$+Na) 282.0742, found 282.0749. ($C_{14}H_{13}NO_3$+Na).

3-methoxyphenyl(4-nitrophenyl)methanol[5] (**3d**)

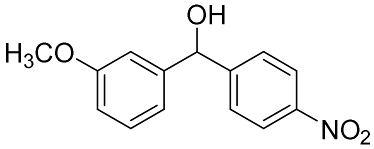

(3-methoxyphenyl)(4-nitrophenyl)methanol (**3d**). The product was isolated as an Oil (120 mg, 93%) after column chromatography purification using a solution of petroleum ether and ethyl acetate 8:1(*v/v*) as the eluent. IR (neat cm^{-1}): 1360(NO_2), 1550(NO_2), 2851(OCH_3), 2932(OCH_3), 3342(OH); The NMR spectra of (3-methoxyphenyl)(4-nitrophenyl)methanol are shown as Attached Fig.7 and Attached Fig.8 in supplementary material, respectively. ^{1}H NMR ($CDCl_3$, 300 MHz) δ 2.78 (brs, 1H), 3.79 (s, 3H), 5.86 (s, 1H), 6.82 (m, 3H), 7.25-7.30 (m, 1H), 7.57 (d, J = 9.0 Hz, 2H), 8.17 (d, J = 9.0 Hz, 2H); ^{13}C NMR ($CDCl_3$, 75 MHz) δ 55.2, 75.3, 112.3, 113.4, 118.9, 123.6, 127.0, 129.9, 144.3, 147.1, 150.6, 159.9; HRMS (ESI) m/z calcd for ($C_{14}H_{13}NO_4$+Na) 282.0742, found 282.0753. ($C_{14}H_{13}NO_3$+Na).

(2-methoxyphenyl)(4-nitrophenyl)methanol[6] (**3e**)

[5] H. M. Zheng, Q. Zhang, J. X. Chen, M. C. Liu, S. H. Cheng, J. C. Ding, H. Y. Wu, W. K. Su, *J. Org. Chem.* **2009**, *74*, 943–945.

[6] H. Zhao, M. Cheng, T. Zhang, M. Z. Cai, *J. Organomet. Chem.* **2015**, *777*, 50–56.

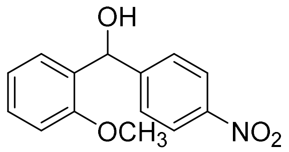

(2-methoxyphenyl)(4-nitrophenyl)methanol (**3e**). The product was isolated as an Oil (118 mg, 91%) after column chromatography purification using a solution of petroleum ether and ethyl acetate 8:1(*v/v*) as the eluent. IR (neat cm^{-1}): 1355(NO_2), 1554(NO_2), 2853(OCH_3), 2935(OCH_3), 3345(OH); The NMR spectra of (2-methoxyphenyl)(4-nitrophenyl)methanol are shown as Attached Fig.9 and Attached Fig.10 in supplementary material, respectively. ^{1}H NMR ($CDCl_3$, 300 MHz) δ 3.17 (s, 1H), 3.80 (s, 3H), 6.09 (s, 1H), 6.89-6.92 (m, 2H), 7.21-7.32 (m, 2H), 7.55 (d, J = 8.8 Hz, 2H), 8.15 (d, J = 8.8 Hz, 2H); ^{13}C NMR ($CDCl_3$, 75 MHz) δ 55.4, 71.6, 110.9, 121.1, 123.4, 127.1, 127.8, 128.2, 129.0, 129.5, 130.6, 150.8; HRMS (ESI) m/z calcd for ($C_{14}H_{13}NO_4$+Na) 282.0742, found 282.0746. ($C_{14}H_{13}NO_3$+Na).

(4-nitrophenyl)(o-tolyl)methanol[7] (**3f**)

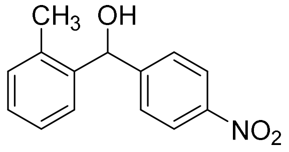

(4-nitrophenyl)(o-tolyl)methanol (**3f**). The product was isolated as an Oil (25.4 mg, 92%) after column chromatography purification using a solution of petroleum ether and ethyl acetate 5:1(*v/v*) as the eluent. IR (neat cm^{-1}): 1355(NO_2), 1554(NO_2), 2870(CH_3), 2960(CH_3), 3345(OH); The NMR spectra of (4-nitrophenyl)(o-tolyl)methanol are shown as Attached Fig.11 and Attached Fig.12 in supplementary material, respectively. ^{1}H NMR ($CDCl_3$, 300 MHz) δ 2.30 (s, 3H), 2.45 (s, 1H), 6.07 (s, 1H), 7.15-7.25 (m, 3H), 7.30-7.33 (m, 1H), 7.50 (d, J = 8.8 Hz, 2H), 8.16 (d, J = 8.8 Hz, 2H); ^{13}C NMR ($CDCl_3$, 75 MHz) δ 19.4, 72.7, 123.6, 126.5, 127.0, 127.5, 128.3, 131.0, 135.6, 140.4, 147.1, 150.2; HRMS (ESI) m/z calcd for ($C_{14}H_{13}NO_3$+Na) 266.0793, found 266.0797. ($C_{14}H_{13}NO_3$+Na).

naphthalen-1-yl(4-nitrophenyl)methanol[5] (**3g**)

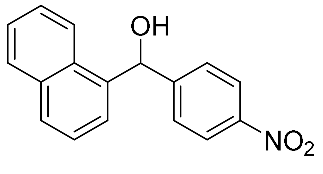

naphthalen-1-yl(4-nitrophenyl)methanol (**3g**). The product was isolated as a light-yellow solid (129 mg, 93%) after column chromatography purification using a solution of petroleum ether and ethyl

[7] T. Zou, S. S. Pi, J. H. Li, *Org. Lett.* **2009**, *11*, 453–456.

acetate 5:1(*v/v*) as the eluent. mp 51–52 °C; IR (neat cm^{-1}): 1380(NO_2), 1564(NO_2), 3345(OH); The NMR spectra of naphthalen-1-yl(4-nitrophenyl)methanol are shown as Attached Fig.13 and Attached Fig.14 in supplementary material, respectively. ^{1}H NMR ($CDCl_3$, 300 MHz) δ 2.79 (brs, 1H), 6.55 (s, 1H), 7.47-7.51 (m, 4H), 7.58 (d, *J* = 8.9 Hz, 2H), 7.85-7.91 (m, 2H), 8.01-8.04 (m, 1H), 8.15 (d, *J* = 8.9 Hz, 2H); ^{13}C NMR ($CDCl_3$, 75 MHz) δ 73.4, 123.6, 123.7, 125.3, 125.6, 126.0, 126.6, 127.5, 129.0, 129.4, 130.5, 134.2, 137.8, 147.3, 150.3; HRMS (ESI) m/z calcd for ($C_{17}H_{13}NO_3$+Na) 302.0793, found 302.0799. ($C_{17}H_{13}NO_3$+Na).

(4-fluorophenyl)(4-nitrophenyl)methanol[5] (**3h**)

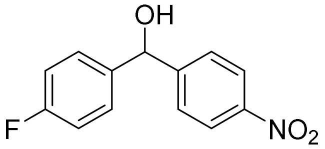

(4-fluorophenyl)(4-nitrophenyl)methanol (**3h**). The product was isolated as a light-yellow solid (117 mg, 95%) after column chromatography purification using a solution of petroleum ether and ethyl acetate 10:1(*v/v*) as the eluent. mp 74–76 °C; IR (neat cm^{-1}): 1384(NO_2), 1562(NO_2), 3350(OH); The NMR spectra of (4-fluorophenyl)(4-nitrophenyl)methanol are shown as Attached Fig.15 and Attached Fig.16 in supplementary material, respectively. ^{1}H NMR ($CDCl_3$, 300 MHz) δ 2.40 (s, 1H), 5.93 (s, 1H), 7.04-7.09 (m, 2H), 7.32-7.36 (m, 2H), 7.57 (d, *J* = 6.9 Hz, 2H), 8.21 (d, *J* = 6.9 Hz, 2H); ^{13}C NMR ($CDCl_3$, 75 MHz) δ 74.8, 115.8 (d, $J\text{-}C_F$ = 21.5 Hz), 123.7, 127.0, 128.5 (d, $J\text{-}C_F$ = 8.2 Hz), 138.5, 147.3, 150.5, 162.5 (d, $J\text{-}C_F$ = 246.2 Hz); HRMS (ESI) m/z calcd for ($C_{13}H_{10}FNO_3$+Na) 270.0542, found 270.0549. ($C_{13}H_{10}FNO_3$+Na)._

(4-chlorophenyl)(4-nitrophenyl)methanol[8] (**3i**)

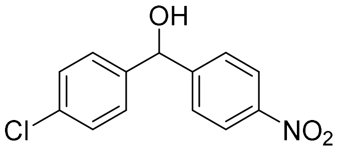

(4-chlorophenyl)(4-nitrophenyl)methanol (**3i**). The product was isolated as a light-yellow solid (121 mg, 92%) after column chromatography purification using a solution of petroleum ether and ethyl acetate 8:1(*v/v*) as the eluent. mp 132–134 °C; IR (neat cm^{-1}): 1386(NO_2), 1566(NO_2), 3348(OH); The NMR spectra of (4-chlorophenyl)(4-nitrophenyl)methanol are shown as Attached Fig.17 and Attached Fig.18 in supplementary material, respectively. ^{1}H NMR ($CDCl_3$, 300 MHz) δ 2.43 (d, *J* =

[8] K. Li, N. F. Hu, R. S.Luo, W. C. Yuan, W. J. Tang. *J. Org. Chem.* **2013**, *78*, 6350–6355.

3.5 Hz"1H), 5.90 (d, J = 3.5 Hz, 1H), 7.27-7.35 (m, 4H), 7.55 (d, J = 8.9 Hz, 2H), 8.19 (d, J = 8.9 Hz, 2H); ^{13}C NMR ($CDCl_3$, 75 MHz) δ 74.6, 123.6, 126.8, 127.8, 127.8, 128.9, 134.0, 140.9, 150.0; HRMS (ESI) m/z calcd for ($C_{13}H_{10}ClNO_3$+Na) 286.0247, found 286.0254. ($C_{13}H_{10}ClNO_3$+Na).

(4-bromophenyl)(4-nitrophenyl)methanol[9] (**3j**)

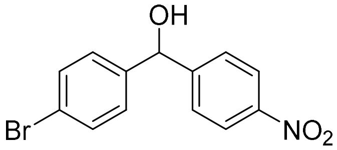

(4-bromophenyl)(4-nitrophenyl)methanol (**3j**). The product was isolated as a light-yellow solid (140 mg, 91%) after column chromatography purification using a solution of petroleum ether and ethyl acetate 6:1(*v/v*) as the eluent. mp 160–162 °C; IR (neat cm^{-1}): 1378(NO_2), 1561(NO_2), 3353(OH); The NMR spectra of (4-bromophenyl)(4-nitrophenyl)methanol are shown as Attached Fig.19 and Attached Fig.20 in supplementary material, respectively. ^{1}H NMR ($CDCl_3$, 300 MHz) δ 2.43 (d, J = 3.3 Hz, 1H), 5.89 (d, J = 3.3 Hz, 1H), 7.23 (d, J = 6.7 Hz, 2H), 7.48-7.56 (m, 4H), 8.20 (d, J = 6.8 Hz, 2H); ^{13}C NMR ($CDCl_3$, 75 MHz) δ 74.5, 122.0, 123.5, 126.7, 128.0, 131.7, 141.3, 147.1, 149.8; HRMS (ESI) m/z calcd for ($C_{13}H_{10}BrNO_3$+Na) 329.9742, found 329.9747. ($C_{13}H_{10}BrNO_3$+Na).

3-nitrophenyl-phenylmethanol[10] (**3k**)

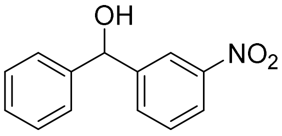

(3-nitrophenyl)(phenyl)methanol (**3j**). The product was isolated as a light-yellow solid (97 mg, 85%) after column chromatography purification using a solution of petroleum ether and ethyl acetate 5:1(*v/v*) as the eluent. mp 68–70 °C; IR (neat cm^{-1}): 1379(NO_2), 1562(NO_2), 3351(OH); The NMR spectra of (3-nitrophenyl)(phenyl)methanol are shown as Attached Fig.21 and Attached Fig.22 in supplementary material, respectively. ^{1}H NMR ($CDCl_3$, 300 MHz) δ 2.75 (s, H), 5.91 (s, 1H), 7.33-7.38 (m, 5H), 7.49 (t, J = 7.9 Hz, 1H), 7.71(d, J = 7.9 Hz, 1H), 8.11 (d, J = 7.9 Hz, 1H), 8.29 (s, 1H); ^{13}C NMR (75 MHz) δ 75.3, 121.3, 122.4, 126.6, 128.3, 128.9, 129.3, 132.4, 142.8, 145.8,148.3; HRMS (ESI) m/z calcd for ($C_{13}H_{11}NO_3$+Na) 252.0637, found 252.0644 ($C_{13}H_{11}NO_3$+Na).

2-nitrophenyl-phenylmethanol[10] (**3l**)

---

9 R. G. La, A. Coluccia, V. Famiglini, S. Pelliccia, L. Monti, D. Vullo, E. Nuti, V. Alterio, G. De Simone, S. M. Monti, P. W. Pan, S. Parkkila, C. T. Supuran, A. Rossello, R. Silvestri, *J. Med. Chem.* **2015**, *58*, 8564–8572;

10 S. J. Chang, S. L. Zhou, H. M. Gau, *RSC Advances*, **2015**, *5*, 9368–9373.

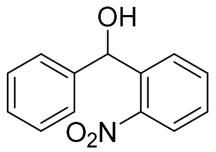

(2-nitrophenyl)(phenyl)methanol(**3j**). The product was isolated as a light-yellow solid (87 mg, 76%) after column chromatography purification using a solution of petroleum ether and ethyl acetate 5:1(*v/v*) as the eluent. mp 58–59 °C; IR (neat cm^{-1}): 1373(NO_2), 1558(NO_2), 3348(OH); The NMR spectra of (2-nitrophenyl)(phenyl)methanol are shown as Attached Fig.23 and Attached Fig.24 in supplementary material, respectively. ^{1}H NMR ($CDCl_3$, 300 MHz) δ 3.04 (s, 1H), 6.40 (s, 1H), 7.29-7.33 (m, 5H), 7.45 (t, J = 7.5 Hz, 1H), 7.63 (t, J = 8.1 Hz, 1H), 7.74 (d, J = 7.5 Hz, 1H), 7.91 (d, J = 8.1 Hz, 1H); ^{13}C NMR ($CDCl_3$, 75 MHz) δ 71.4, 124.7,126.9, 128.0, 128.5, 128.5, 129.4, 133.4, 138.5, 141.5, 148.3; HRMS (ESI) m/z calcd for ($C_{13}H_{11}NO_3$+Na) 252.0637, found 252.0645 ($C_{13}H_{11}NO_3$+Na).

4-cyanophenyl-phenylmethanol[11] (**3m**)

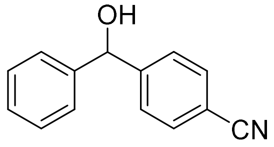

4-(hydroxy(phenyl)methyl)benzonitrile (**3m**). The product was isolated as a light-yellow solid (91 mg, 87%) after column chromatography purification using a solution of petroleum ether and ethyl acetate 6:1(*v/v*) as the eluent. mp 68–70 °C; IR (neat cm^{-1}): 2221(CN), 3348(OH); The NMR spectra of 4-(hydroxy(phenyl)methyl)benzonitrile are shown as Attached Fig.25 and Attached Fig.26 in supplementary material, respectively. ^{1}H NMR ($CDCl_3$, 300 MHz) δ 2.31 (d, J = 3.0 Hz, 1H), 5.87 (d, J = 3.0 Hz, 1H), 7.33-7.34 (m, 5H), 7.52 (d, J = 8.6 Hz, 2H), 7.63 (d, J = 8.6 Hz, 2H); ^{13}C NMR ($CDCl_3$, 75 MHz) δ 75.6, 118.8, 126.6, 126.7, 127.0, 127.0, 128.3, 128.9, 132.2, 142.8; HRMS (ESI) m/z calcd for ($C_{14}H_{11}NO$+Na) 232.0738, found 232.0734 ($C_{14}H_{11}NO$+Na).

4-formylphenyl-phenylmethanol[11] (**3n**)

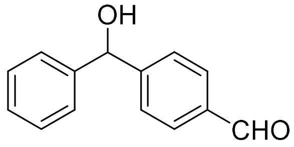

4-formylphenyl-phenylmethanol(**3n**). The product was isolated as a light-yellow solid (98 mg, 93%)

[11] a) Y. Hayashi, N. Yamamura, T. Kusukawa, T. Harada, *Chem. Eur. J.*, **2016,** 22, 12095–12105; b) J. Karthikeyan, M. Jeganmohan, C. H Cheng, *Chem. Eur. J.* **2010**, *16*, 8989–8992.

after column chromatography purification using a solution of petroleum ether and ethyl acetate 5:1(*v/v*) as the eluent. mp 76–78 °C; IR (neat cm^{-1}): 1710(CHO), 2720(CHO), 2749(CHO), 3348(OH); The NMR spectra of 4-formylphenyl-phenylmethanol are shown as Attached Fig.27 and Attached Fig.28 in supplementary material, respectively. ^{1}H NMR ($CDCl_3$, 300 MHz) δ 2.88 (brs, 1H), 5.88 (s, 1H), 7.27-7.36 (m, 5H), 7.56 (d, J = 8.0 Hz, 2H), 7.83 (d, J = 8.0 Hz, 2H), 9.94 (s, 1H); ^{13}C NMR ($CDCl_3$, 75 MHz) δ 75.8, 126.7, 126.9, 128.0, 128.7, 129.9, 135.5, 143.1, 150.5, 192.1; HRMS (ESI) m/z calcd for ($C_{14}H_{12}O_2$+Na) 235.0735, found 235.0742 ($C_{14}H_{12}O_2$+Na).

methyl 4-(hydroxy(phenyl)methyl)benzoate[12] (**3o**)

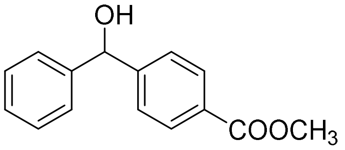

methyl 4-(hydroxy(phenyl)methyl)benzoate(**3o**). The product was isolated as a light-yellow solid (66 mg, 55%) after column chromatography purification using a solution of petroleum ether and ethyl acetate 5:1(*v/v*) as the eluent. mp 61–62 °C; IR (neat cm^{-1}): 1730(COO), 3351(OH); The NMR spectra of methyl 4-(hydroxy(phenyl)methyl)benzoate are shown as Attached Fig.29 and Attached Fig.30 in supplementary material, respectively. ^{1}H NMR ($CDCl_3$, 300 MHz) δ 2.59 (brs, 1H), 3.89 (s, 3H), 5.86 (s, 1H), 7.19-7.35 (m, 5H), 7.45 (d, J = 8.3 Hz, 2H), 7.99 (d, J = 8.3 Hz, 2H); ^{13}C NMR ($CDCl_3$, 75 MHz) δ 52.1, 75.9, 126.3, 126.6, 127.9, 128.7, 129.2, 129.8, 143.3, 148.7, 166.9; HRMS (ESI) m/z calcd for ($C_{15}H_{14}O_3$+Na) 265.0841, found 265.0847 ($C_{15}H_{14}O_3$+Na).

(4-(methylsulfonyl)phenyl)(phenyl)methanol[5] (**3p**)

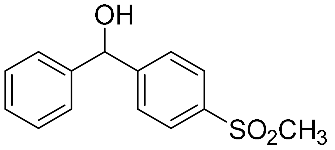

(4-(methylsulfonyl)phenyl)(phenyl)methanol(**3p**). The product was isolated as a light-yellow solid (119 mg, 91%) after column chromatography purification using a solution of petroleum ether and ethyl acetate 5:1(*v/v*) as the eluent. mp 125–126 °C; IR (neat cm^{-1}): 1560(SO_2), 3351(OH); The NMR spectra of (4-(methylsulfonyl)phenyl)(phenyl)methanol are shown as Attached Fig.31 and Attached Fig.32 in supplementary material, respectively. ^{1}H NMR ($CDCl_3$, 300 MHz) δ 2.42 (s, 1H), 3.03 (s, 3H), 5.91 (d, J = 2.5 Hz, 1H), 7.33-7.37 (m, 5H), 7.61 (d, J = 8.1 Hz, 2H), 7.90 (d, J = 8.1

[12] T. Yamamoto, T. Furusawa, A. Zhumagazin, T. Yamakawa, Y. Oe, T. Ohta, *Tetrahedron*, **2015**, *71*, 19–26.

Hz, 2H); ^{13}C NMR ($CDCl_3$, 75 MHz) δ 44.5, 75.6, 126.7, 127.2, 127.5, 127.5, 128.2, 128.8, 142.9, 150.0; HRMS (ESI) m/z calcd for ($C_{14}H_{14}O_3S$+Na) 285.0561, found 285.0567 ($C_{14}H_{14}O_3S$+Na).

4. Experimental characterization data for benzil derivatives

Benzyl[13] (**4a**)

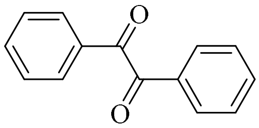

Benzyl(**4a**). The product was isolated as a light-yellow solid (99 mg, 95%) after column chromatography purification using a solution of petroleum ether and ethyl acetate 10:1(*v/v*) as the eluent. mp 94–95 °C; IR (neat cm^{-1}): 1680(-CO-CO-), 1620(-CO-CO-); The NMR spectra of benzyl are shown as Attached Fig.33 and Attached Fig.34 in supplementary material, respectively. ^{1}H NMR ($CDCl_3$, 300 MHz) δ 7.48-7.54 (m, 4H), 7.62-7.68 (m, 2H), 7.95-8.00 (m, 4H); ^{13}C NMR ($CDCl_3$, 75 MHz) δ 128.9, 129.5, 132.2, 134.7, 194.5; HRMS (ESI) m/z calcd for ($C_{14}H_{10}O_2$+H) 211.2400, found 211.2410 ($C_{15}H_{12}O_3$+H).

1-(4-methoxyphenyl)-2-phenylethane-1, 2-dione[14](**4b**)

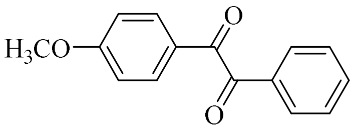

1-(4-methoxyphenyl)-2-phenylethane-1,2-dione(**4b**). The product was isolated as a light-yellow solid (113 mg, 94%) after column chromatography purification using a solution of petroleum ether and ethyl acetate 10:1(*v/v*) as the eluent. mp 62–63 °C; IR (neat cm^{-1}): 2830(OCH_3), 2812(OCH_3), 1680(-CO-CO-), 1620(-CO-CO-); The NMR spectra of 1-(4-methoxyphenyl)-2-phenylethane-1,2-dione are shown as Attached Fig.35 and Attached Fig.36 in supplementary material, respectively. ^{1}H NMR ($CDCl_3$, 300 MHz) δ 3.89 (s, 3H), 6.94-6.99 (m, 2H), 7.46-7.64 (m, 3H), 7.91-7.99 (m, 8.4 Hz, 4H); ^{13}C NMR ($CDCl_3$, 75 MHz) δ 55.6, 114.4, 126.0, 128.9, 129.8, 133.2, 134.6, 140.0, 165.0, 193.1, 194.8; HRMS (ESI) m/z calcd for ($C_{15}H_{12}O_3$+H) 241.0865, found 241.0873 ($C_{15}H_{12}O_3$+H).

1-phenyl-2-*p*-tolylethane-1, 2-dione[14] (**4c**)

16 Z. H. Wan, C. D. Jones, D. Mitchell, J. Y. Pu, T. Y. Zhang, *J. Org. Chem.* **2006**, *71*, 826–828.

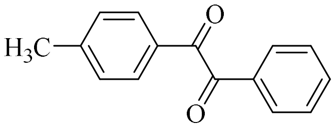

1-phenyl-2-(p-tolyl)ethane-1,2-dione(**4c**). The product was isolated as a light-yellow solid (104 mg, 93%) after column chromatography purification using a solution of petroleum ether and ethyl acetate 10:1(*v/v*) as the eluent. mp 99–101 °C; IR (neat cm^{-1}): 2958(CH_3), 2861(CH_3), 1680(-CO-CO-), 1620(-CO-CO-); The NMR spectra of 1-phenyl-2-(p-tolyl)ethane-1,2-dione are shown as Attached Fig.37 and Attached Fig.38 in supplementary material, respectively. ^{1}H NMR ($CDCl_3$, 300 MHz) δ 2.43 (s, 3H), 7.28-7.31 (m, 2H), 7.46-7.52 (m, 2H), 7.61-7.64 (m, 1H), 7.85-7.88 (m, 2H), 7.94-7.98 (m, 2H); ^{13}C NMR ($CDCl_3$, 75 MHz) δ 21.9, 128.9, 129.7, 129.8, 129.9, 130.0, 130.6, 134.8, 146.2, 194.2, 194.7; HRMS (ESI) m/z calcd for ($C_{15}H_{12}O_2$+H) 225.0916, found 225.0922 ($C_{15}H_{12}O_2$+H).

1-(naphthalen-1-yl)-2-phenylethane-1,2-dione[14] (**4d**)

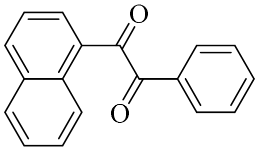

1-(naphthalen-1-yl)-2-phenylethane-1,2-dione(**4d**). The product was isolated as a light-yellow solid (119 mg, 92%) after column chromatography purification using a solution of petroleum ether and ethyl acetate 10:1(*v/v*) as the eluent. mp 101–103 °C; IR (neat cm^{-1}): 1688(-CO-CO-), 1622(-CO-CO-); The NMR spectra of 1-(naphthalen-1-yl)-2-phenylethane- 1,2-dione are shown as Attached Fig.39 and Attached Fig.40 in supplementary material, respectively. ^{1}H NMR ($CDCl_3$, 300 MHz) δ 7.49-7.67 (m, 6H), 7.90-8.05 (m, 5H), 9.30-9.46 (m, 1H); ^{13}C NMR ($CDCl_3$, 75 MHz) δ 124.5, 126.0, 127.2, 128.6, 129.0, 129.5, 131.0, 133.4, 134.1, 134.8, 135.1, 136.0, 194.6, 197.2; HRMS (ESI) m/z calcd for ($C_{18}H_{12}O_2$+H) 261.0916, found 261.0923 ($C_{18}H_{12}O_2$+H).

1-(4-fluorophenyl)-2-phenylethane-1, 2-dione[15] (**4e**)

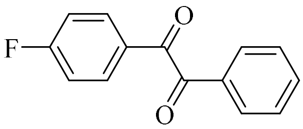

1-(4-fluorophenyl)-2-phenylethane-1,2-dione(4e). The product was isolated as a light-yellow solid (104 mg, 91%) after column chromatography purification using a solution of petroleum ether and

15 E. Anders, T. Clark, T. Gassner, *Chem. Ber.* **1986**, *119*, 1350–1360.

13 R. Ramajayam, R. Giridhar, M. R. Yadav, *Chem. Heterocycl. Compd.* **2006,** *42*, 901–906.

ethyl acetate 10:1(*v/v*) as the eluent. mp 62–64 °C; IR (neat cm^{-1}): 1688(-CO-CO-), 1622(-CO-CO-); The NMR spectra of 1-(4-fluorophenyl)-2-phenylethane-1,2-dione are shown as Attached Fig.41 and Attached Fig.42 in supplementary material, respectively. ^{1}H NMR ($CDCl_3$, 300 MHz) δ 7.15-7.26 (m, 2H), 7.50-7.54 (m, 2H), 7.55-7.68 (m, 1H), 7.95-8.05 (m, 4H); ^{13}C NMR ($CDCl_3$, 75 MHz) δ 116.2, 116.5, 129.0, 129.5, 129.6, 129.9, 132.6, 132.7, 132.9, 134.9, 165.0, 168.5, 192.6, 194.0; HRMS (ESI) m/z calcd for ($C_{14}H_9FO_2$+H) 229.0665, found 229.0669 ($C_{14}H_9FO_2$+H).

1-phenyl-2-*p*-tolylethane-1, 2-dione[14] (**4f**)

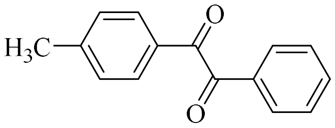

1-phenyl-2-(p-tolyl)ethane-1,2-dione(**4f**). The product was isolated as a light-yellow solid (104 mg, 93%) after column chromatography purification using a solution of petroleum ether and ethyl acetate 10:1(*v/v*) as the eluent. mp 99–101 °C; IR (neat cm^{-1}): 2958(CH_3), 2861(CH_3), 1680(-CO-CO-), 1620(-CO-CO-); The NMR spectra of 1-phenyl-2-(p-tolyl)ethane-1,2-dione are shown as Attached Fig.43 and Attached Fig.44 in supplementary material, respectively. ^{1}H NMR ($CDCl_3$, 300 MHz) δ 2.43 (s, 3H), 7.28-7.31 (m, 2H), 7.46-7.52 (m, 2H), 7.61-7.64 (m, 1H), 7.85-7.88 (m, 2H), 7.94-7.98 (m, 2H); ^{13}C NMR ($CDCl_3$, 75 MHz) δ 21.9, 128.9, 129.7, 129.8, 129.9, 130.0, 130.6, 134.8, 146.2, 194.2, 194.7; HRMS (ESI) m/z calcd for ($C_{15}H_{12}O_2$+H) 225.0916, found 225.0922 ($C_{15}H_{12}O_2$+H).

1-(4-methoxyphenyl)-2-phenylethane-1, 2-dione[14](**4g**)

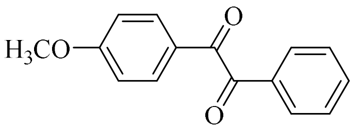

1-(4-methoxyphenyl)-2-phenylethane-1,2-dione(**4b**). The product was isolated as a light-yellow solid (113 mg, 94%) after column chromatography purification using a solution of petroleum ether and ethyl acetate 15:1(*v/v*) as the eluent. mp 62–63 °C; IR (neat cm^{-1}): 2830(OCH_3), 2812(OCH_3), 1680(-CO-CO-), 1620(-CO-CO-); The NMR spectra of 1-(4-methoxyphenyl)-2-phenylethane-1,2-dione are shown as Attached Fig.45 and Attached Fig.46 in supplementary material, respectively. ^{1}H NMR ($CDCl_3$, 300 MHz) δ 3.89 (s, 3H), 6.94-6.99 (m, 2H), 7.46-7.64 (m, 3H), 7.91-7.99 (m, 8.4 Hz, 4H); ^{13}C NMR ($CDCl_3$, 75 MHz) δ 55.6, 114.4, 126.0, 128.9, 129.8, 133.2, 134.6, 140.0, 165.0, 193.1, 194.8; HRMS (ESI) m/z calcd for ($C_{15}H_{12}O_3$+H) 241.0865, found 241.0873 ($C_{15}H_{12}O_3$+H).

1-phenyl-2-(thiophen-3-yl) ethane-1, 2-dione[16](**4h**)

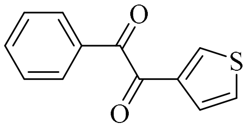

1-phenyl-2-(thiophen-3-yl) ethane-1, 2-dione(**4h**). The product was isolated as a light-yellow solid (72 mg, 67%) after column chromatography purification using a solution of petroleum ether and ethyl acetate 15:1(*v/v*) as the eluent. mp 81–82 °C; IR (neat cm^{-1}): 3011(thiophene), 1620(-CO-CO-); The NMR spectra of 1-phenyl-2-(thiophen-3-yl) ethane-1, 2-dione are shown as Attached Fig.47 and Attached Fig.48 in supplementary material, respectively. ^1H NMR ($CDCl_3$, 300 MHz) δ 7.39-7.54 (m, 3H), 7.63-7.70 (m, 2H), 7.99-8.02 (m, 2H), 8.21(s, 1H); ^{13}C NMR ($CDCl_3$, 75 MHz) δ 120.0, 129.3, 129.8, 131.1, 131.2, 132.7, 135.0, 141.5, 193.0, 193.8; HRMS (ESI) m/z calcd for ($C_{12}H_8O_2S$+H) 217.0323, found 217.0328 ($C_{12}H_8O_2S$+H).

1-phenyl-2-(thiophen-2-yl) ethane-1, 2-dione[17](**4i**)

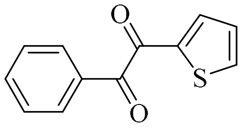

1-phenyl-2-(thiophen-2-yl) ethane-1, 2-dione(**4i**). The product was isolated as a light-yellow solid (25.4 mg, 59%) after column chromatography purification using a solution of petroleum ether and ethyl acetate 5:1(*v/v*) as the eluent. mp 61–62 °C; IR (neat cm^{-1}): 3010(thiophene), 1682(-CO-CO-), 1620(-CO-CO-); The NMR spectra of 1-phenyl-2-(thiophen-2-yl) ethane-1,2-dione are shown as Attached Fig.49 and Attached Fig.50 in supplementary material, respectively. ^1H NMR ($CDCl_3$, 300 MHz) δ 7.19-7.23 (m, 1H), 7.50-7.68 (m, 2H), 7.69-7.86 (m, 3H), 8.07 (d, J = 7.9, 2H); ^{13}C NMR ($CDCl_3$, 75 MHz) δ 128.7, 128.9, 130.2, 132.6, 134.8, 136.6, 136.8, 139.9, 185.5, 192.0; HRMS (ESI) m/z calcd for ($C_{12}H_8O_2S$+H) 217.0323, found 217.0331 ($C_{12}H_8O_2S$+H).

1-(2-chlorophenyl)-2-phenylethane-1,2-dione[17](**4j**)

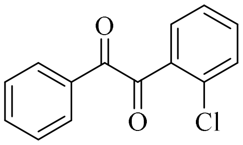

1-(2-chlorophenyl)-2-phenylethane-1,2-dione (**4i**). The product was isolated as a light-yellow solid

17 C. J. Walsh, B. K. Mandal, *J. Org. Chem.* **1999**, *64*, 6102–6105.

18 S. Newman, G. B. Iiahlei, *J. Org. Chem.* **1958**, *23*, 666–669.

(95 mg, 78%) after column chromatography purification using a solution of petroleum ether and ethyl acetate 8:1(*v/v*) as the eluent. mp 47–49 °C; IR (neat cm^{-1}): 1685(-CO-CO-), 1622(-CO-CO-); The NMR spectra of 1-(2-chlorophenyl)-2-phenylethane-1,2-dione are shown as Attached Fig.51 and Attached Fig.52 in supplementary material, respectively. ^{1}H NMR ($CDCl_3$, 300 MHz) δ 7.41-7.57 (m, 5H), 7.64-7.67 (m, 1H), 7.89-7.94 (m, 1H), 8.02-8.05 (m, 2H); ^{13}C NMR ($CDCl_3$, 75 MHz) δ 127.4, 128.9, 130.2, 130.5, 132.1, 132.4, 133.8, 134.0, 134.5, 134.6, 192.1, 193.7; HRMS (ESI) m/z calcd for ($C_{14}H_9ClO_2$+H) 245.0369, found 245.0374 ($C_{14}H_9ClO_2$+H).

1-phenyl-2-o-tolylethane-1,2-dione[18](**4k**)

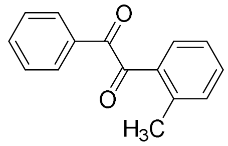

1-phenyl-2-o-tolylethane-1,2-dione(**4k**). The product was isolated as a light-yellow solid (97 mg, 87%) after column chromatography purification using a solution of petroleum ether and ethyl acetate 5:1(*v/v*) as the eluent. mp 57–59 °C; IR (neat cm^{-1}): 2960(CH_3), 2869(CH_3), 1680(-CO-CO-), 1620(-CO-CO-); The NMR spectra of 1-phenyl-2-o-tolylethane-1,2-dione are shown as Attached Fig.53 and Attached Fig.54 in supplementary material, respectively. ^{1}H NMR ($CDCl_3$, 300 MHz) δ 2.72 (s, 3H), 7.27-7.40 (m, 2H), 7.48-7.55 (m, 3H), 7.64-7.70 (m, 2H), 7.97-7.80 (m, 2H); ^{13}C NMR ($CDCl_3$, 75 MHz) δ 21.9, 126.0, 127.9, 129.0, 129.9, 131.8, 132.6, 133.1, 133.8, 134.7, 141.3, 194.8, 196.8; HRMS (ESI) m/z calcd for ($C_{15}H_{12}O_2$+H) 225.0916, found 225.0921 ($C_{15}H_{12}O_2$+H).

1-(naphthalen-1-yl)-2-phenylethane-1,2-dione[19] (**4l**)

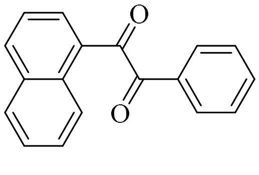

1-(naphthalen-1-yl)-2-phenylethane-1,2-dione(**4l**). The product was isolated as a light-yellow solid (118 mg, 91%) after column chromatography purification using a solution of petroleum ether and ethyl acetate 10:1(*v/v*) as the eluent. mp 101–102 °C; IR (neat):1680(-CO-CO-), 1620(-CO-CO-) cm^{-1}; The NMR spectra of 1-(naphthalen-1-yl)-2-phenylethane-1,2-dione are shown as Attached Fig.55 and Attached Fig.56 in supplementary material, respectively. ^{1}H NMR ($CDCl_3$, 300 MHz) δ

19 J. Tatsugi, Y. Izawa, *J. Chem. Res. S.* **1988**, 356–357.

15 E. Anders, T. Clark, T. Gassner, *Chem. Ber.* **1986**, *119*, 1350–1360.

7.49-7.67 (m, 6H), 7.90-8.05 (m, 5H), 9.30-9.46 (m, 1H); ^{13}C NMR ($CDCl_3$, 75 MHz) δ 124.5, 126.0, 127.2, 128.6, 129.0, 129.5, 131.0, 133.4, 134.1, 134.8, 135.1, 136.0, 194.6, 197.2; HRMS (ESI) m/z calcd for ($C_{18}H_{12}O_2$+H) 261.0916, found 261.0923 ($C_{18}H_{12}O_2$+H).

1-(4-methoxyphenyl)-2-p-tolylethane-1,2-dione[20](**4m**)

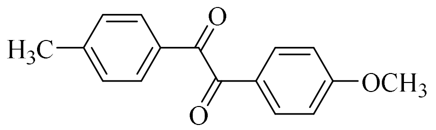

1-(4-methoxyphenyl)-2-p-tolylethane-1,2-dione(**4l**). The product was isolated as a light-yellow solid (117 mg, 92%) after column chromatography purification using a solution of petroleum ether and ethyl acetate 10:1(*v/v*) as the eluent. mp 97–98 °C; IR (neat): 2950(CH_3), 2860(OCH_3), 2812(OCH_3), 1680(-CO-CO-), 1620(-CO-CO-)cm^{-1}; The NMR spectra of 1-(4-methoxyphenyl)-2-p-tolylethane-1,2-dione are shown as Attached Fig.57 and Attached Fig.58 in supplementary material, respectively. ^{1}H NMR ($CDCl_3$, 300 MHz) δ 2 .43 (s, 3H), 3.88 (s, 3H), 6.97 (d, J = 8.9, 2H), 7.30 (d, J = 7.9, 2H), 7.87 (d, J = 8.2, 2H), 7.94 (d, J = 8.2, 2H); ^{13}C NMR ($CDCl_3$, 75 MHz) δ 127.4, 128.9, 130.2, 130.5, 132.1, 132.4, 133.8, 134.0, 134.5, 134.6, 192.1, 193.7; HRMS (ESI) m/z calcd for ($C_{16}H_{14}O_3$+H) 255.1021, found 255.1028 ($C_{16}H_{14}O_3$+H).

1, 2-bis(4-methoxyphenyl)ethane-1,2-dione[21](**4n**)

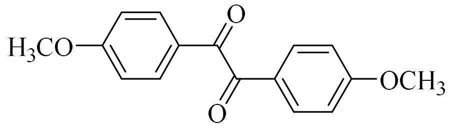

1,2-bis(4-methoxyphenyl)ethane-1,2-dione (**4l**). The product was isolated as a light-yellow solid (127 mg, 94%) after column chromatography purification using a solution of petroleum ether and ethyl acetate 10:1(*v/v*) as the eluent. mp 130–132 °C; IR (neat): 2860(OCH_3), 2812(OCH_3), 1680(-CO-CO-), 1620(-CO-CO-); The NMR spectra of 1,2-bis(4-methoxyphenyl)ethane-1,2-dione are shown as Attached Fig.59 and Attached Fig.60 in supplementary material, respectively. ^{1}H NMR ($CDCl_3$, 300 MHz) δ 3.87 (s, 6H), 6.96 (dd, J_1 = 8.9 Hz, J_2 = 1.95 Hz, 4H), 7.94 (dd, J_1 = 8.9 Hz, J_2 = 1.98 Hz, 4H); ^{13}C NMR ($CDCl_3$, 75 MHz) δ 55.5, 114.2, 126.2, 132.4, 164.7, 193.4; HRMS (ESI) m/z calcd for ($C_{16}H_{14}O_4$+H) 271.0970, found 271.0968 ($C_{16}H_{14}O_4$+H).

20 U. Wille, J. Andropof, *Aust. J. Chem.* **2007**, *60*, 420–428.

21 J. K. Joseph, S. L. Jain, B. Sain, *Eur. J. Org. Chem.* **2006**, 590–594.

1,2-dip-tolylethane-1,2-dione[20](**4o**)

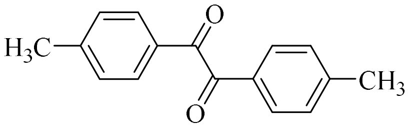

1,2-dip-tolylethane-1,2-dione(**4l**). The product was isolated as a light-yellow solid (118 mg, 93%) after column chromatography purification using a solution of petroleum ether and ethyl acetate 10:1(*v/v*) as the eluent. mp 106–107 °C; IR (neat): 2960(CH_3), 2869(CH_3), 1680(-CO-CO-), 1620(-CO-CO-); The NMR spectra of 1,2-dip-tolylethane-1,2-dione are shown as Attached Fig.61 and Attached Fig.62 in supplementary material, respectively. ^{1}H NMR ($CDCl_3$, 300 MHz) δ 2.42 (s, 6H), 7.29 (dd, J_1 = 8.46 Hz, J_2 = 0.5 Hz, 4H), 7.86 (dd, J_1 = 6.54 Hz, J_2 = 1.7 Hz, 4H), 8.02-8.05 (m, 2H); ^{13}C NMR ($CDCl_3$, 75 MHz) δ 21.8, 129.6, 129.9, 130.6, 146.0, 194.4; HRMS (ESI) m/z calcd for ($C_{16}H_{14}O_2$+H) 239.1072, found 255. 239.1076 ($C_{16}H_{14}O_2$+H).

5. The NMR spectra of diarylmethanols

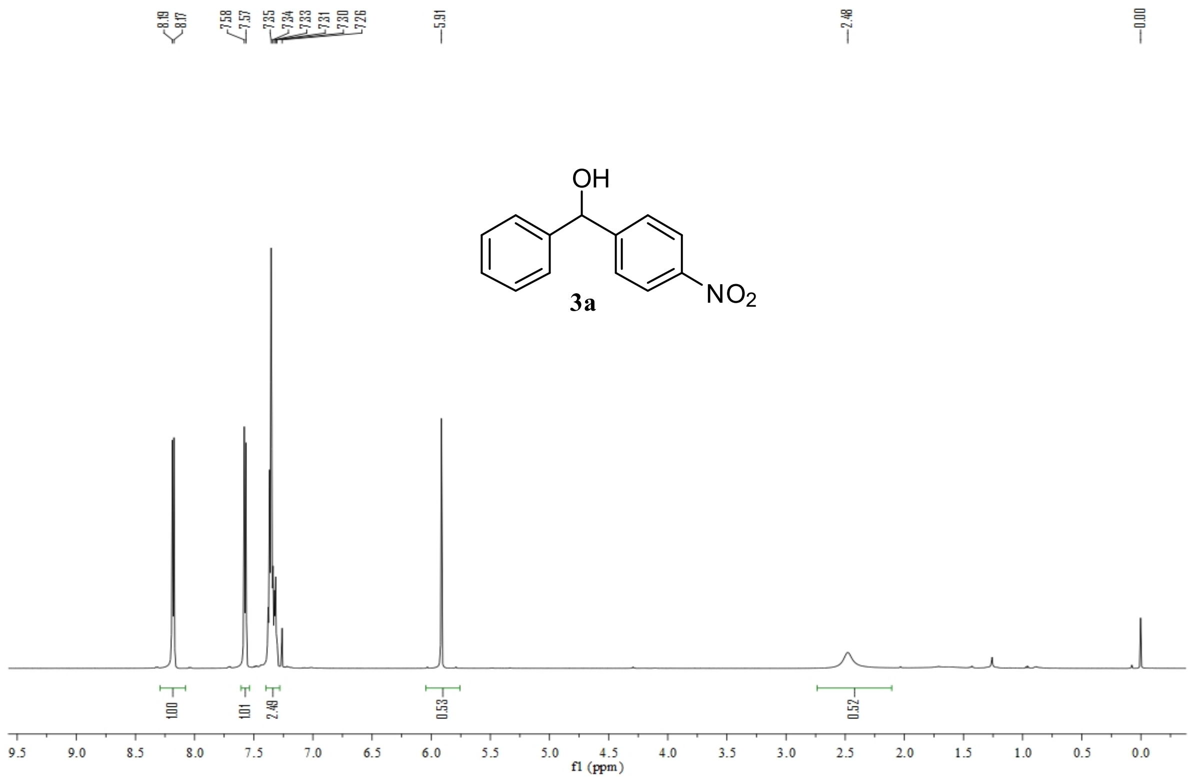

Attached Fig.1 ^{1}H NMR spectrum of (4-nitrophenyl)(phenyl)methanol recorded in $CDCl_3$

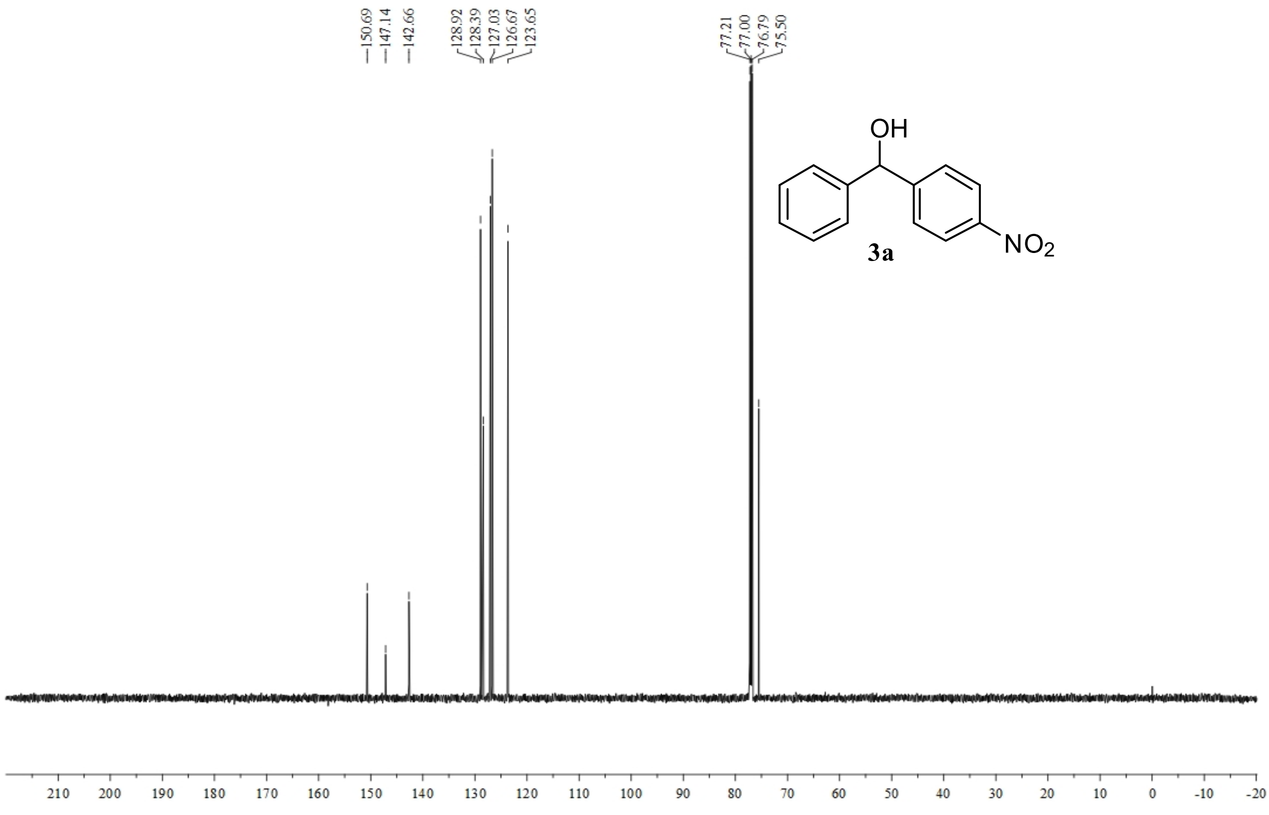

Attached Fig.2 ^{13}C NMR spectrum of (4-nitrophenyl)(phenyl)methanol recorded in $CDCl_3$

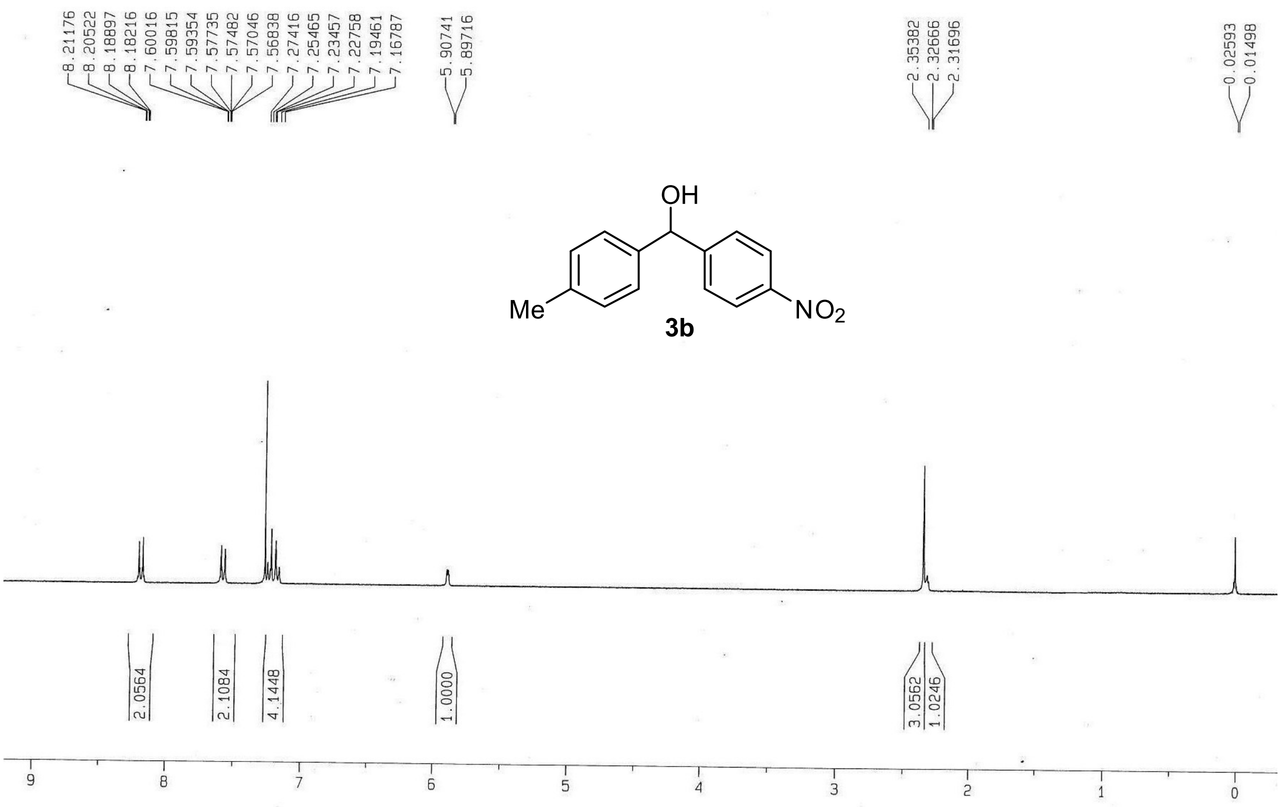

Attached Fig.3 ^{1}H NMR spectrum of (4-nitrophenyl)(*p*-tolyl)methanol recorded in $CDCl_3$

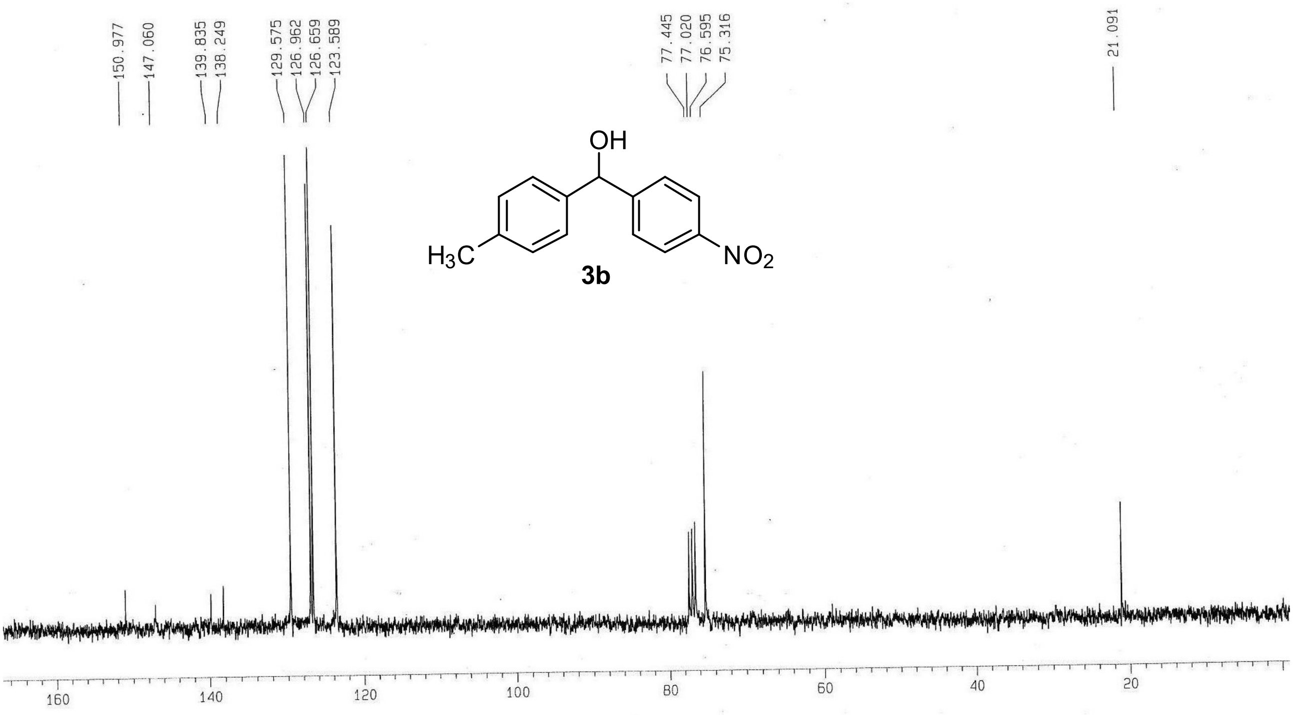

Attached Fig.4 ^{13}C NMR spectrum of (4-nitrophenyl)(*p*-tolyl)methanol recorded in $CDCl_3$

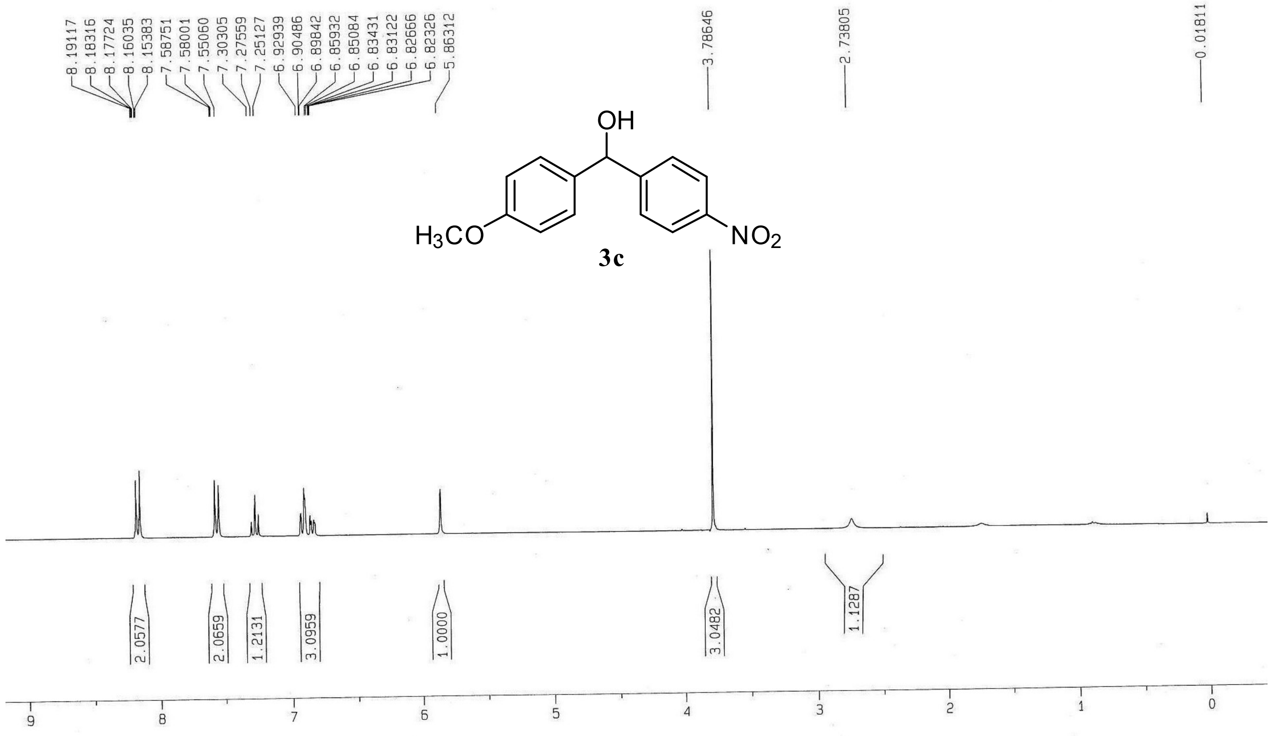

Attached Fig.5 ^{1}H NMR spectrum of (4-methoxyphenyl)(4-nitrophenyl)methanol recorded in $CDCl_3$

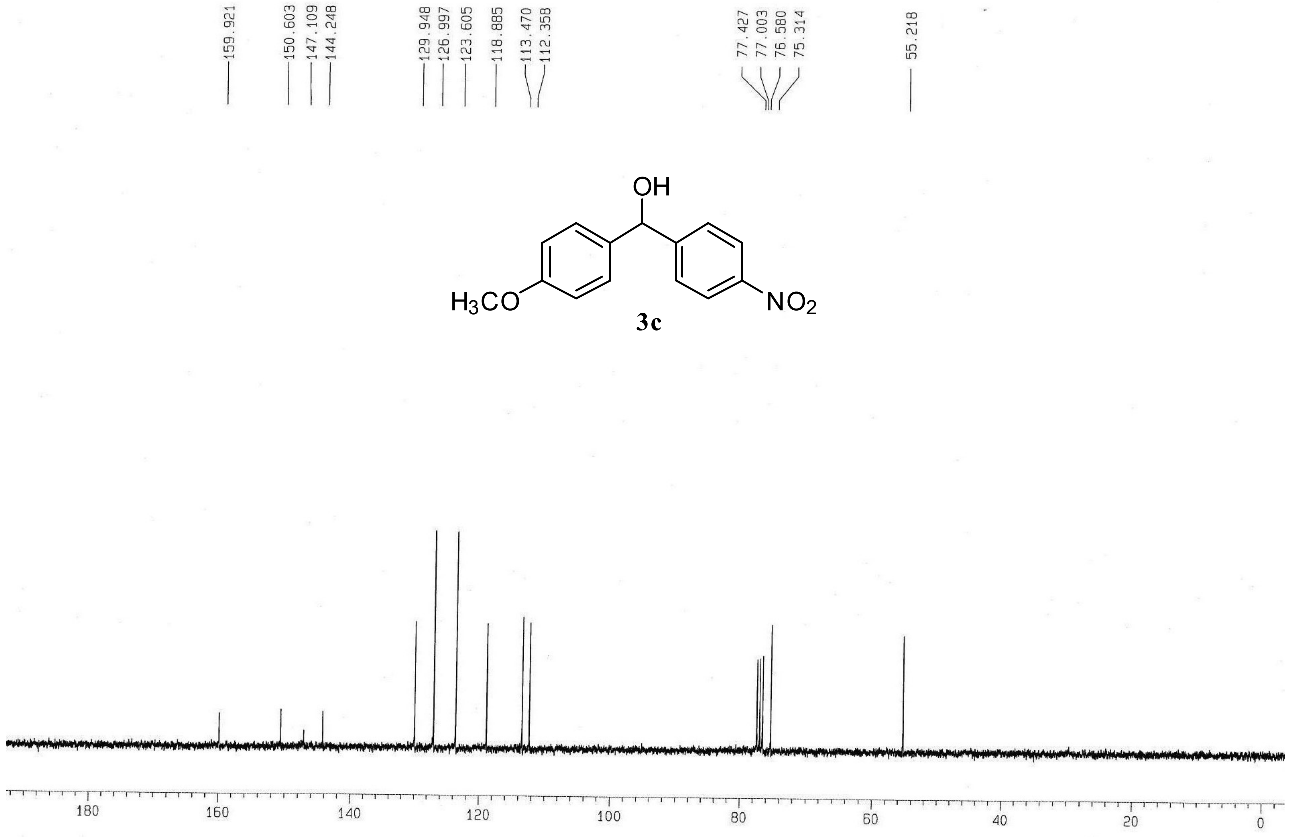

Attached Fig.6 ^{13}C NMR spectrum of (4-methoxyphenyl)(4-nitrophenyl)methanol recorded in $CDCl_3$

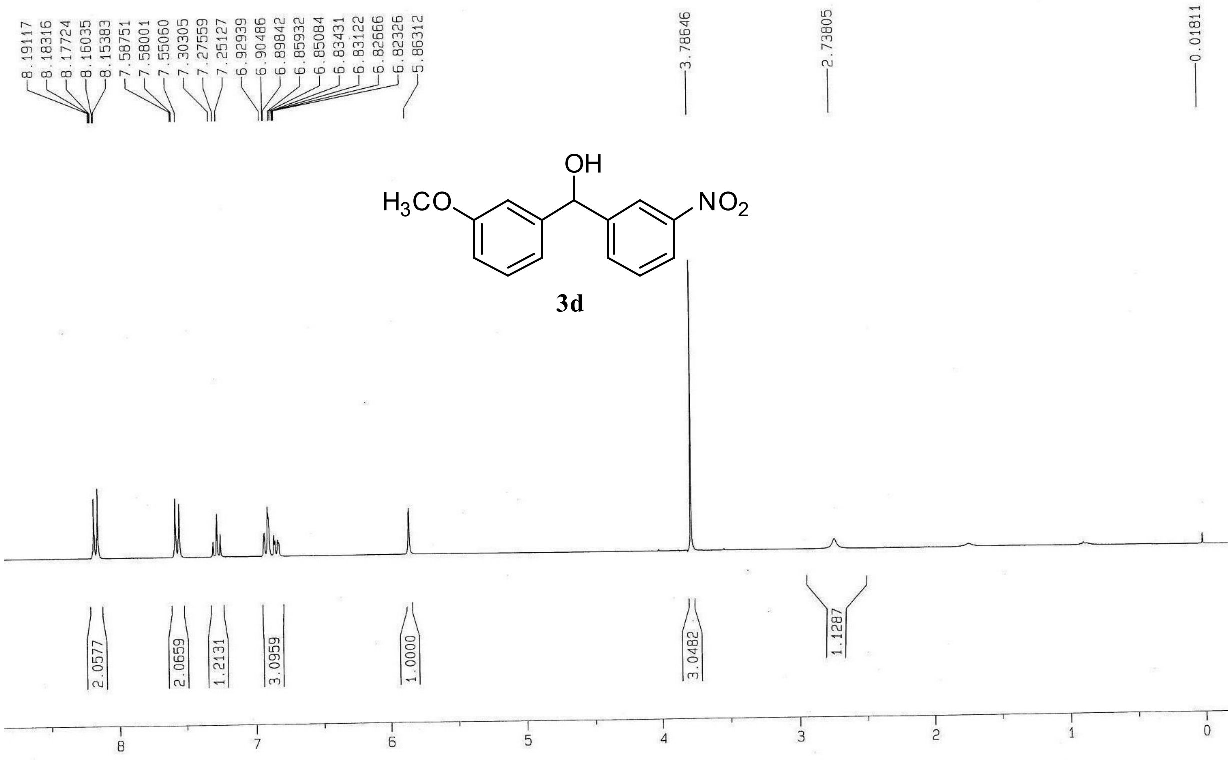

Attached Fig.7 ^{1}H NMR spectrum of (3-methoxyphenyl)(4-nitrophenyl)methanol recorded in $CDCl_3$

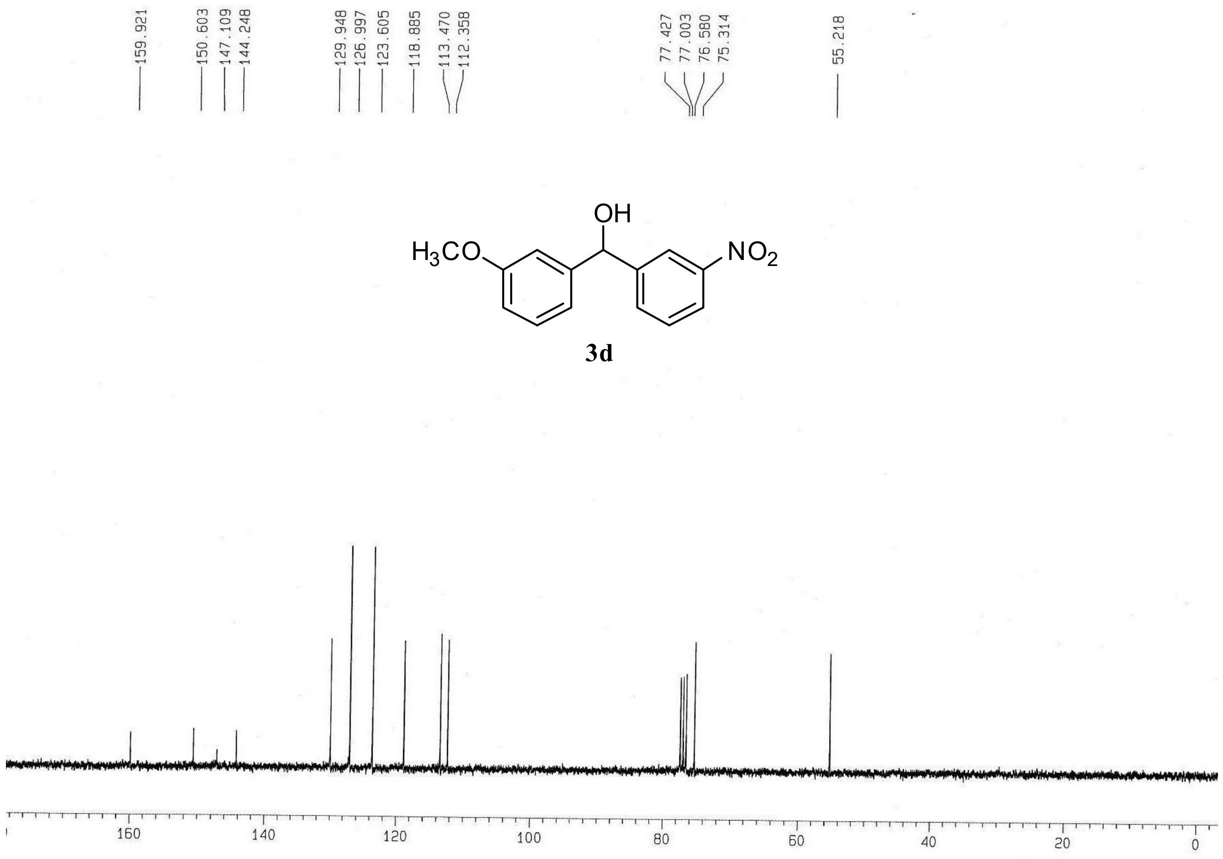

Attached Fig.8 ^{13}C NMR spectrum of (3-methoxyphenyl)(4-nitrophenyl)methanol recorded in $CDCl_3$

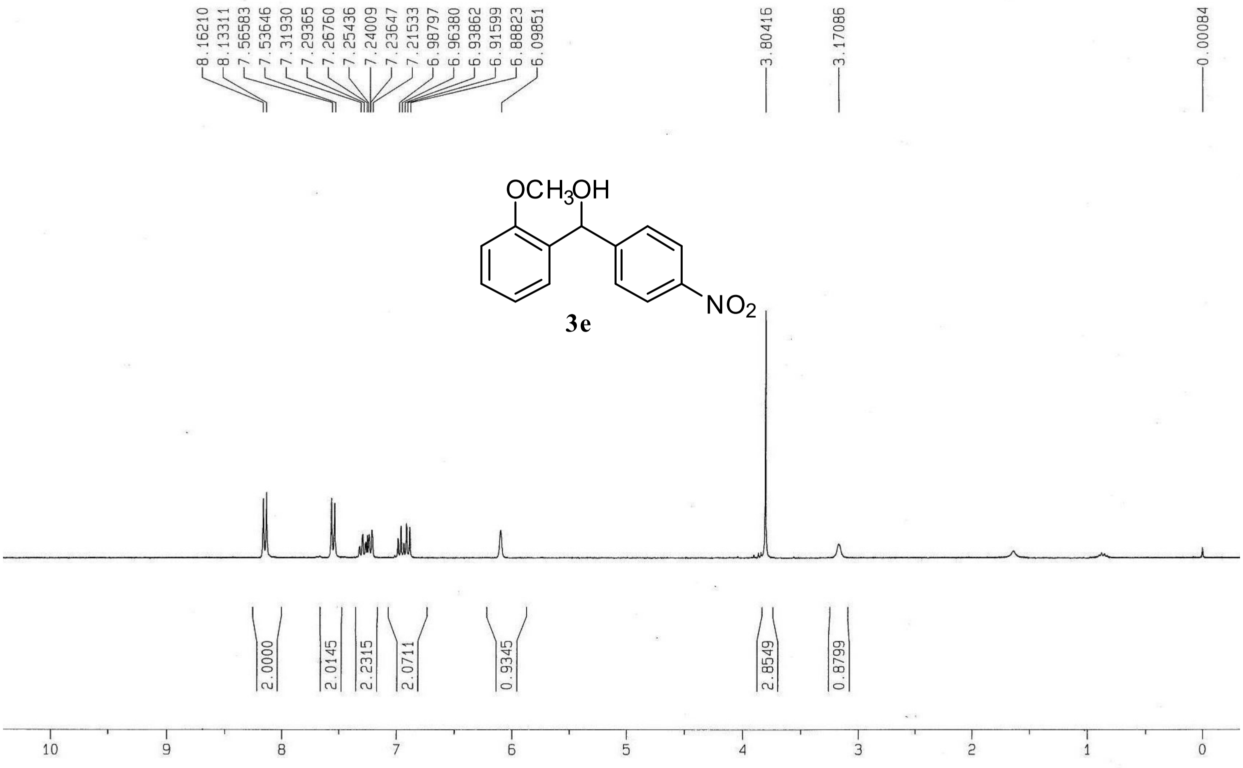

Attached Fig.9 ^{1}H NMR spectrum of (2-methoxyphenyl)(4-nitrophenyl)methanol recorded in $CDCl_3$

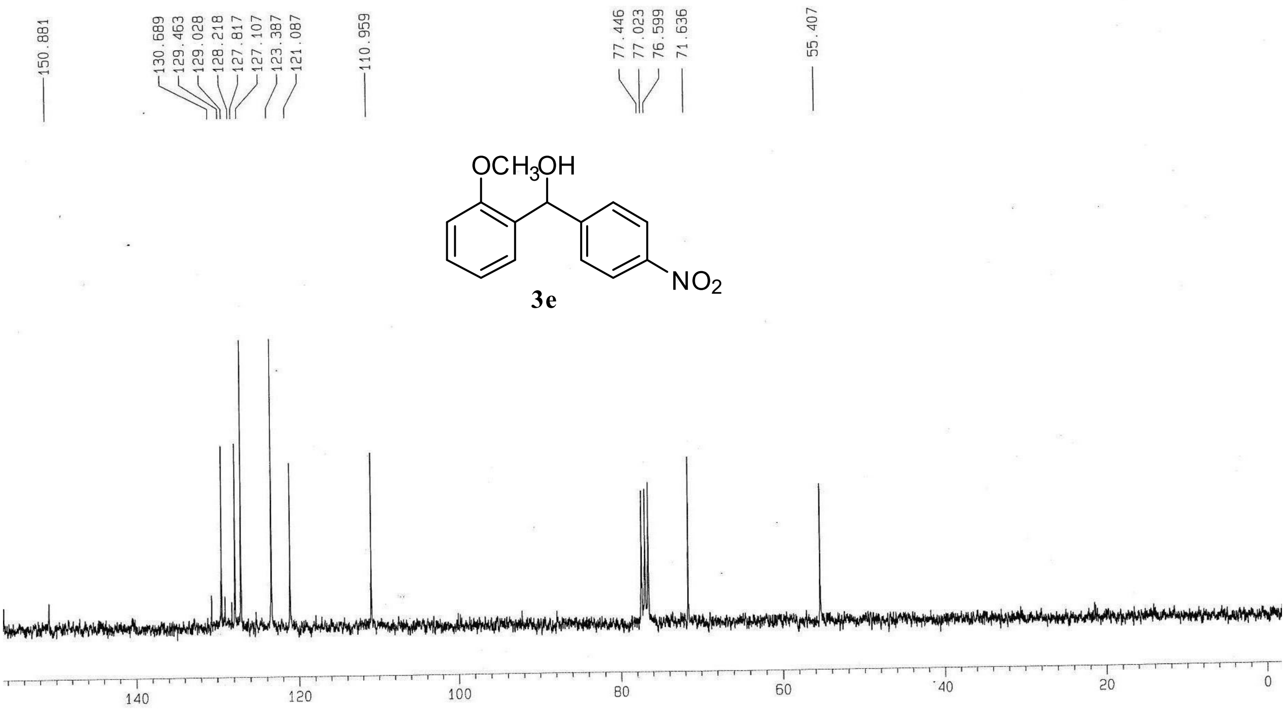

Attached Fig.10 ^{13}C NMR spectrum of (2-methoxyphenyl)(4-nitrophenyl)methanol recorded in $CDCl_3$

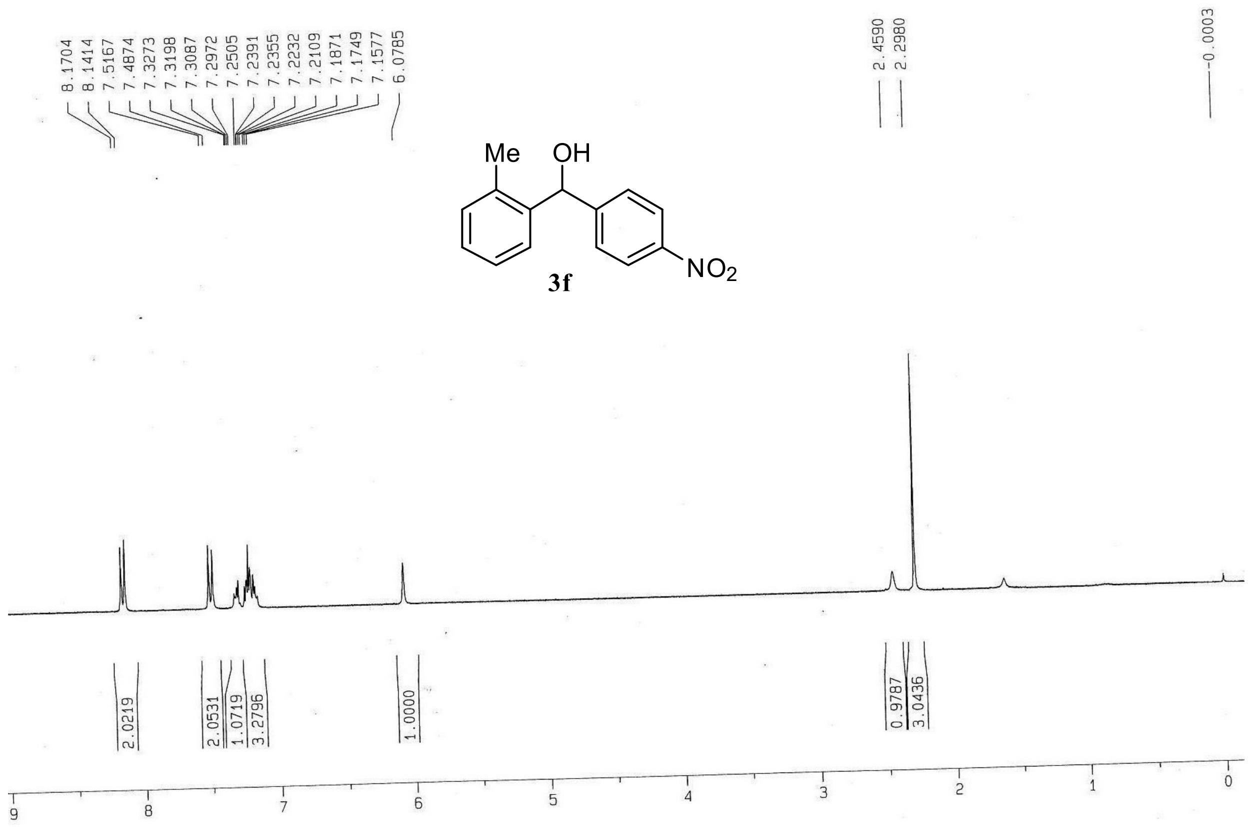

Attached Fig.11 ^{1}H NMR spectrum of (2-methoxyphenyl)(4-nitrophenyl)methanol recorded in $CDCl_3$

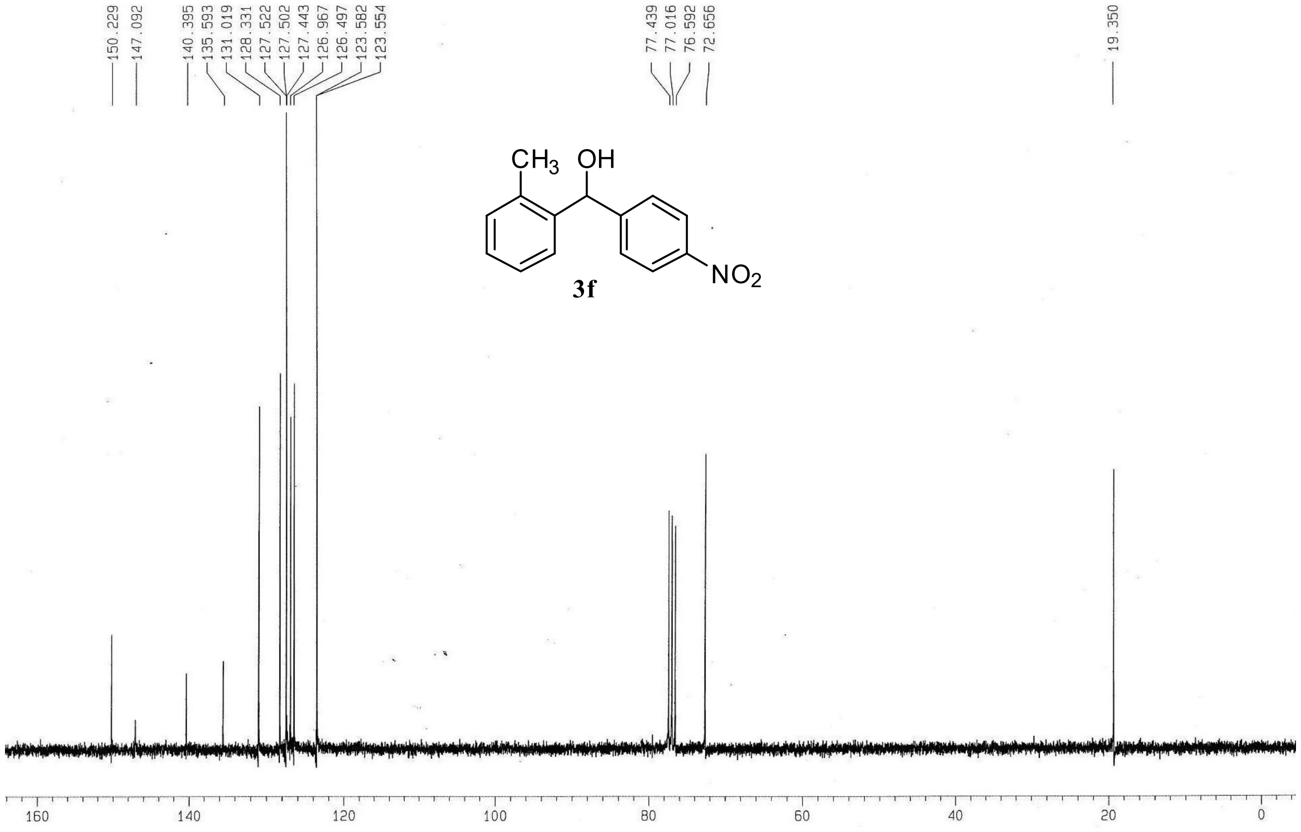

Attached Fig.12 ^{13}C NMR spectrum of (2-methoxyphenyl)(4-nitrophenyl)methanol recorded in $CDCl_3$

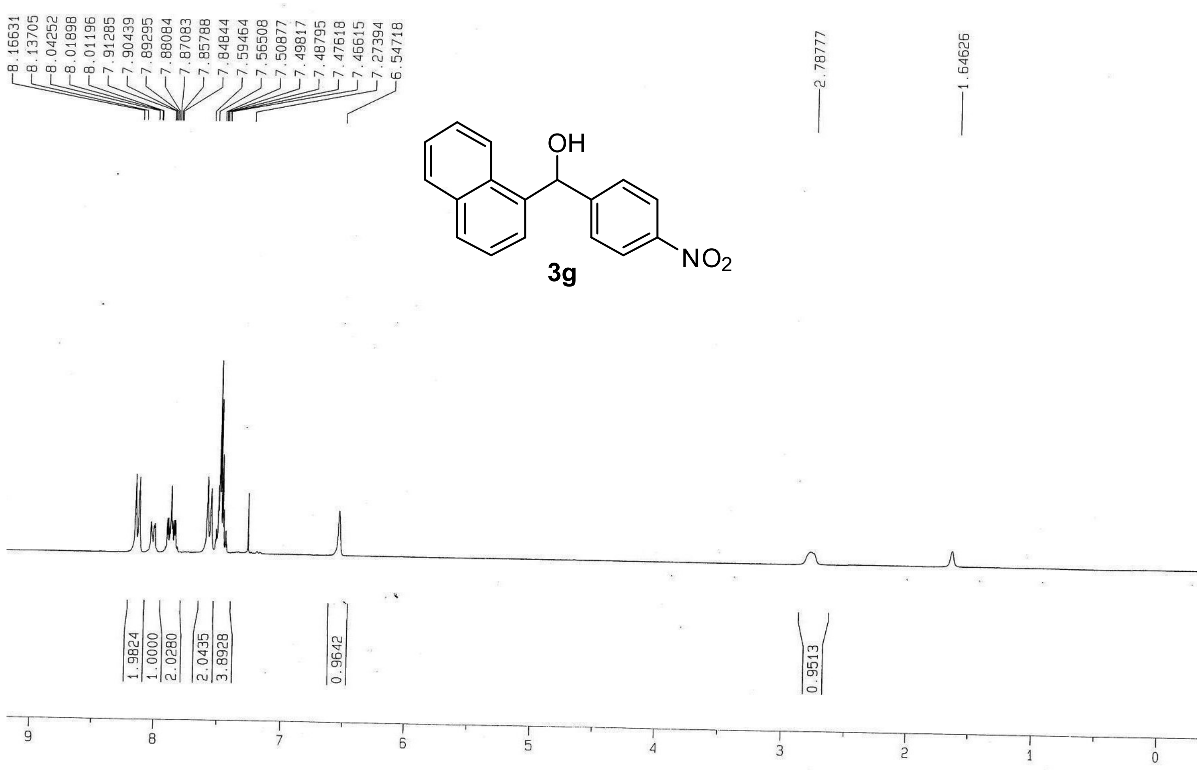

Attached Fig.13 ^{1}H NMR spectrum of naphthalen-1-yl(4-nitrophenyl)methanol recorded in $CDCl_3$

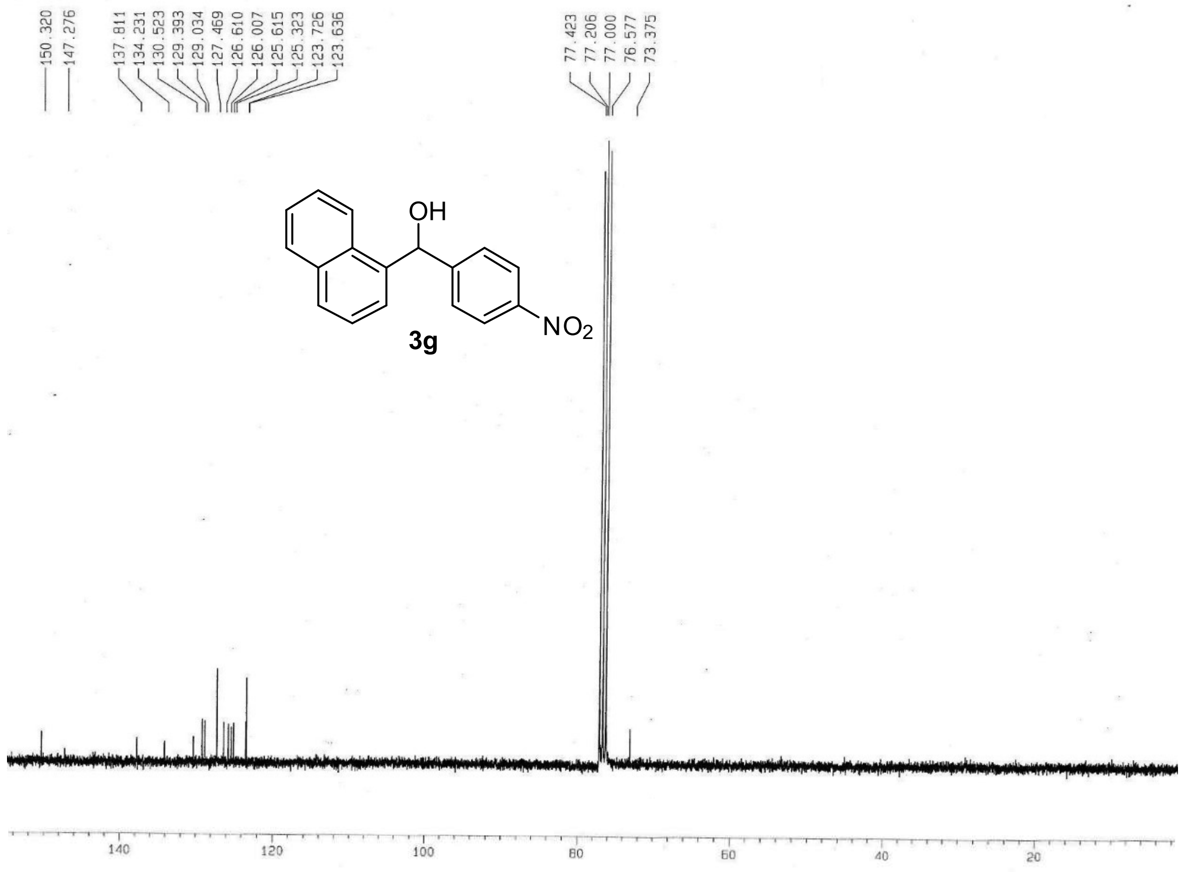

Attached Fig.14 ^{13}C NMR spectrum of naphthalen-1-yl(4-nitrophenyl)methanol recorded in $CDCl_3$

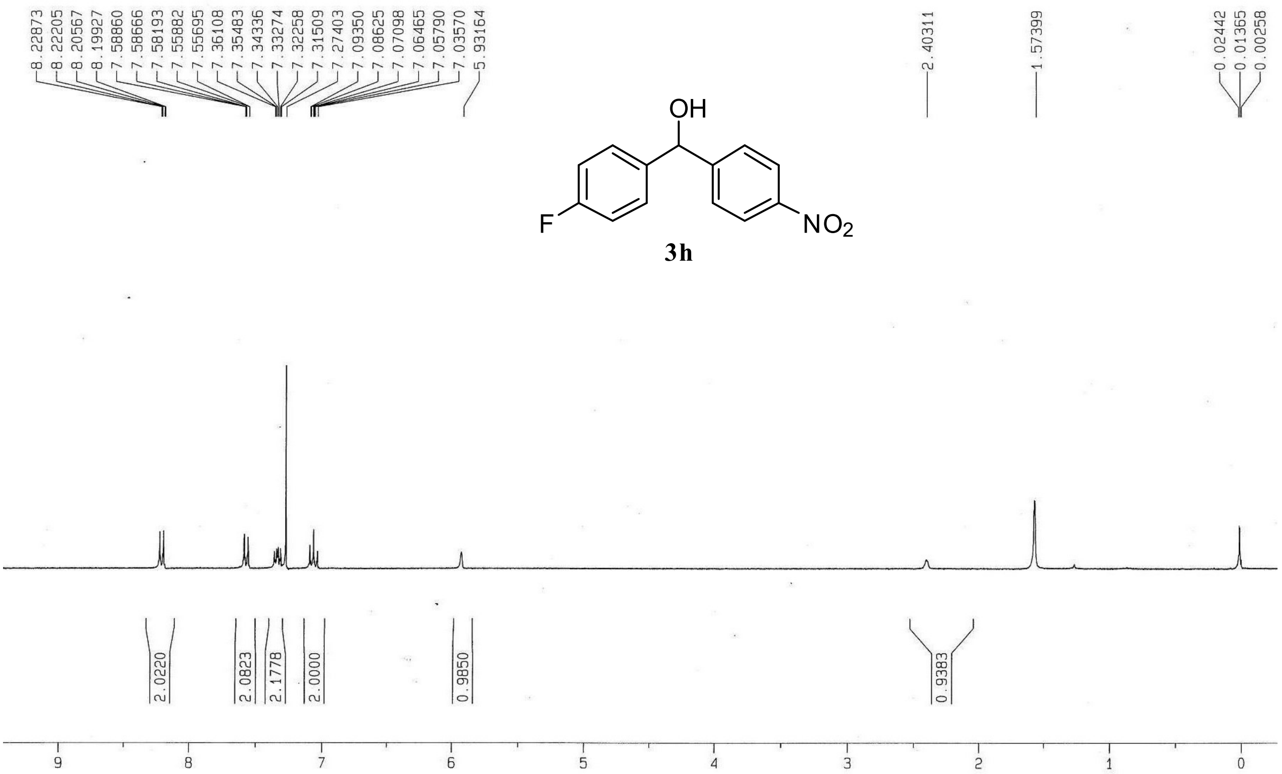

Attached Fig.15 ^{1}H NMR spectrum of (4-fluorophenyl)(4-nitrophenyl)methanol recorded in $CDCl_3$

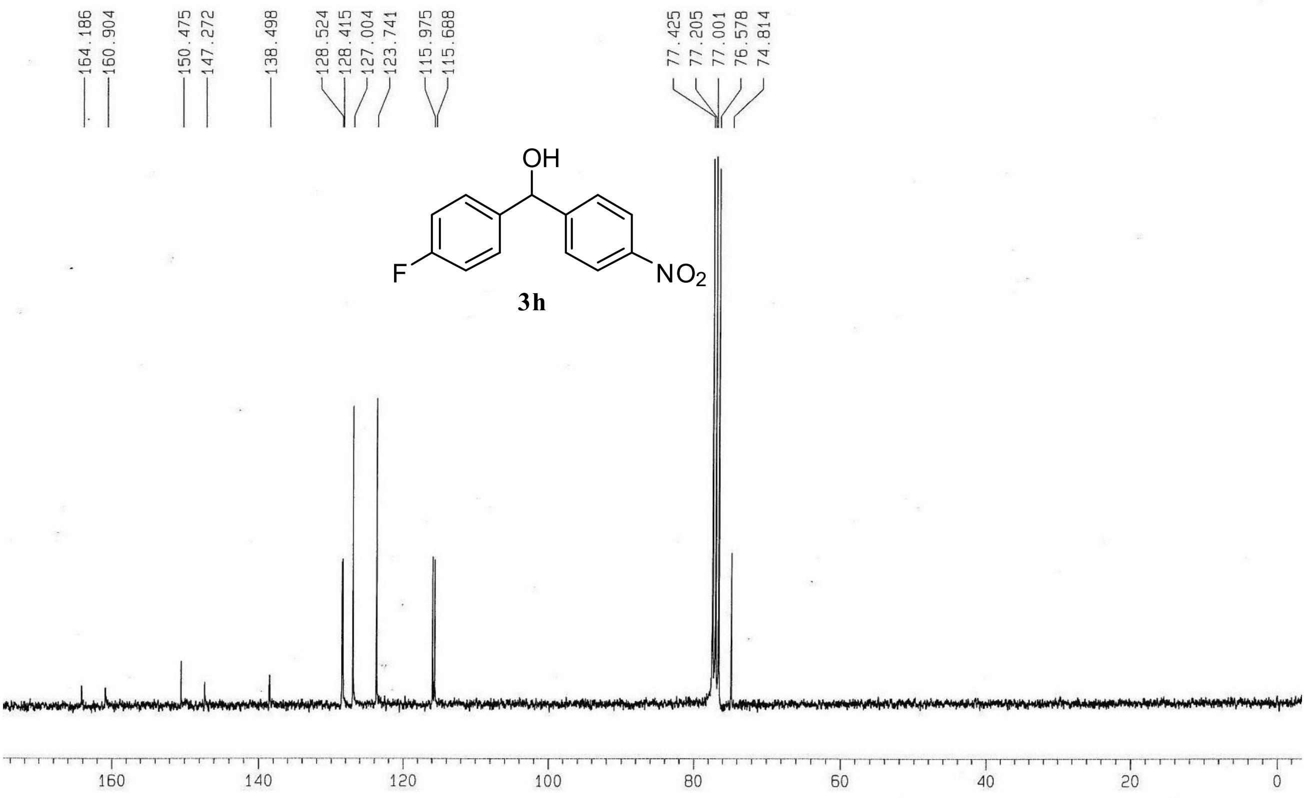

Attached Fig.16 ^{13}C NMR spectrum of (4-fluorophenyl)(4-nitrophenyl)methanol recorded in $CDCl_3$

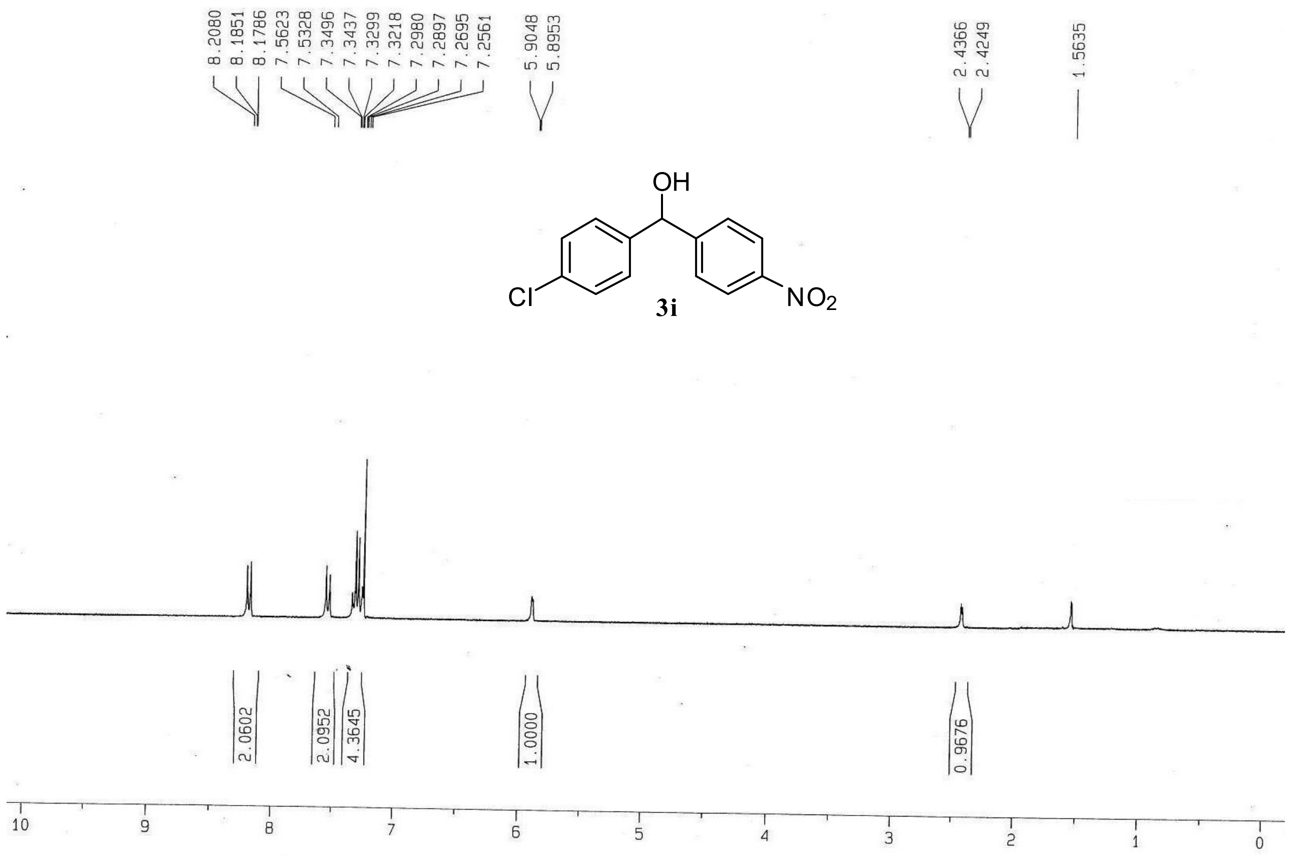

Attached Fig.17 ^{1}H NMR spectrum of (4-chlorophenyl)(4-nitrophenyl)methanol recorded in $CDCl_3$

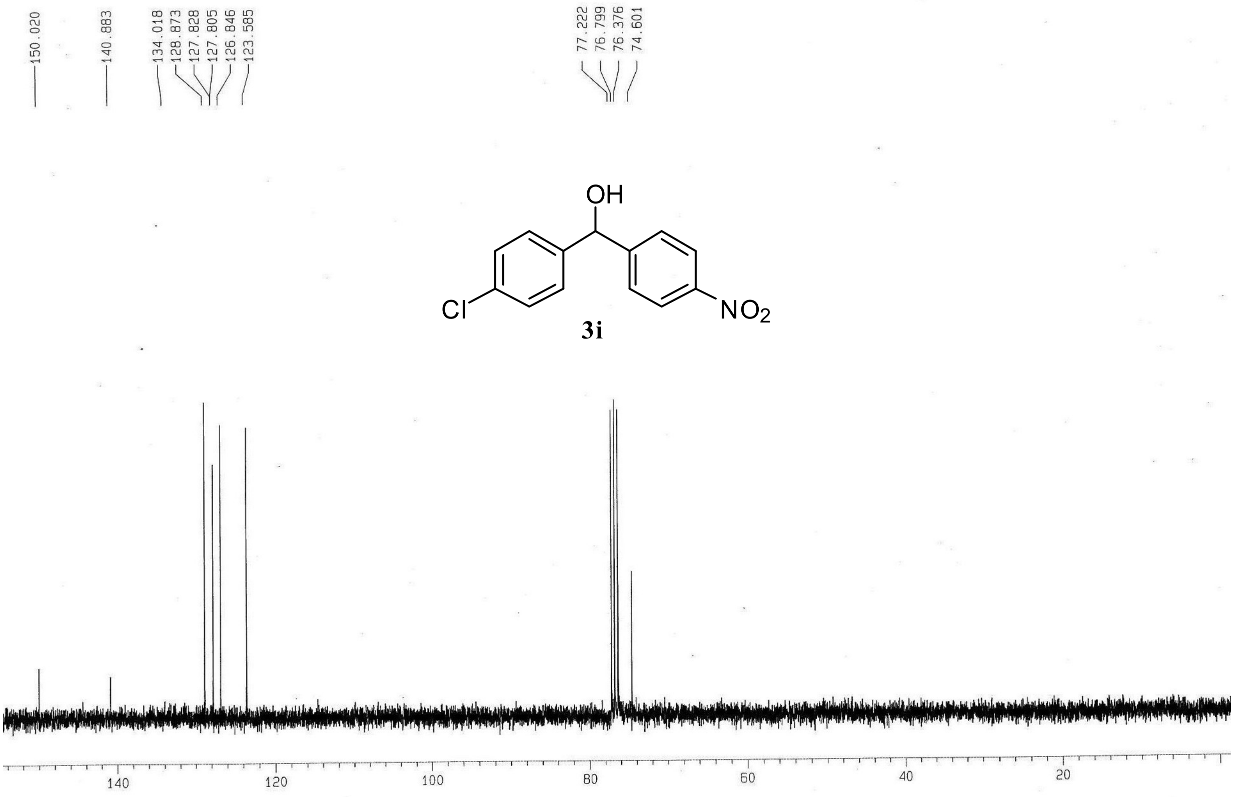

Attached Fig.18 ^{13}C NMR spectrum of (4-chlorophenyl)(4-nitrophenyl)methanol recorded in $CDCl_3$

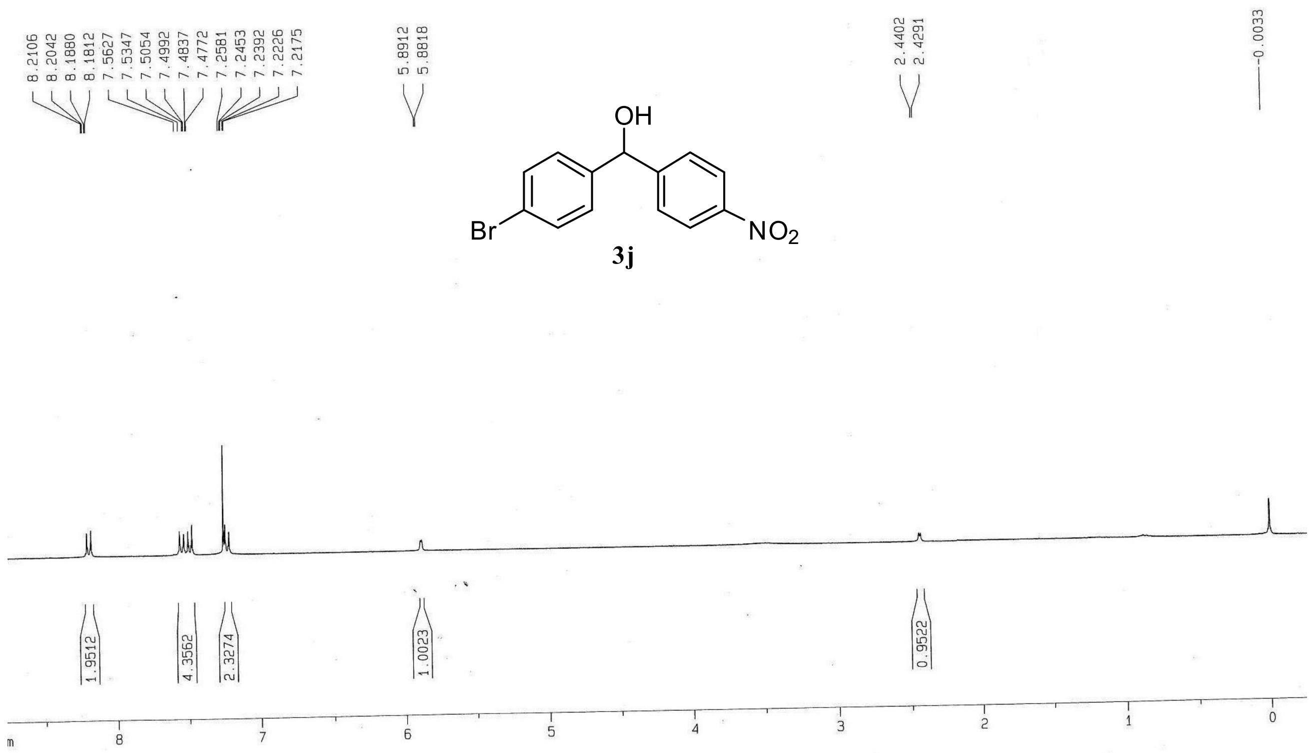

Attached Fig.19 ^{1}H NMR spectrum of (4-bromophenyl)(4-nitrophenyl)methanol recorded in $CDCl_3$

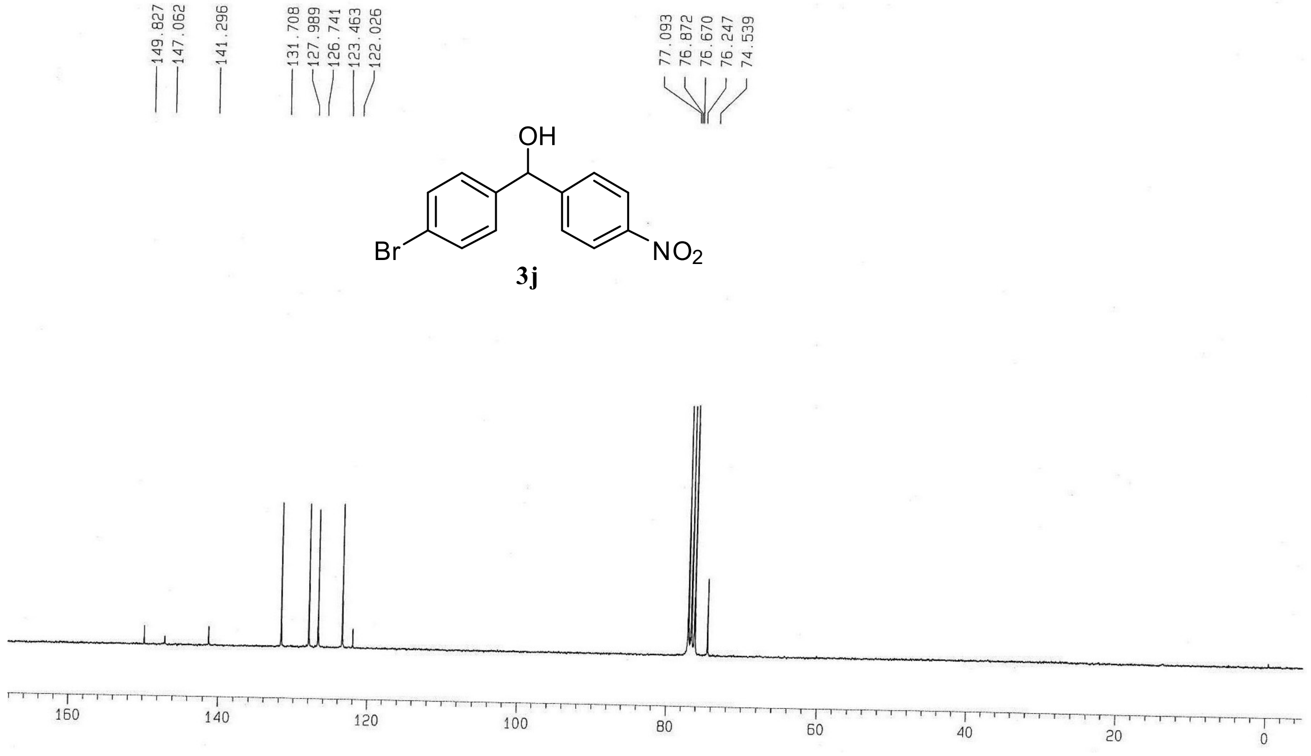

Attached Fig.20 ^{13}C NMR spectrum of (4-bromophenyl)(4-nitrophenyl)methanol recorded in $CDCl_3$

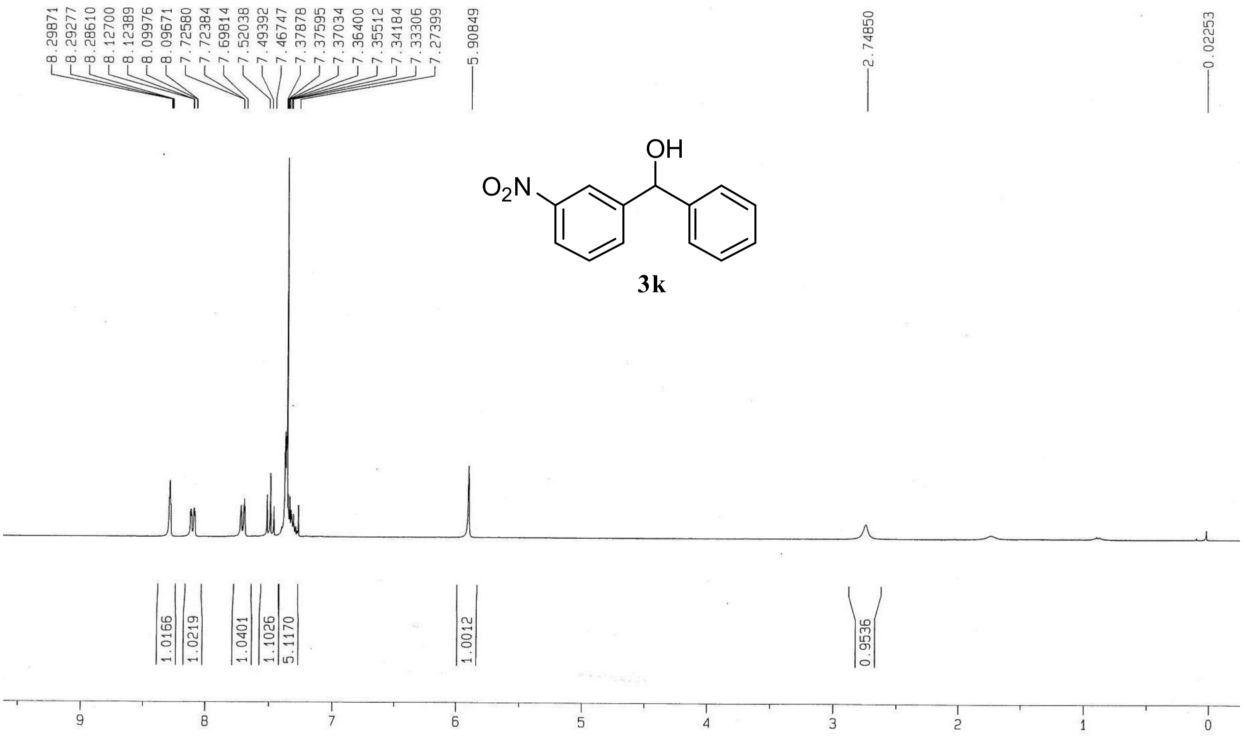

Attached Fig.21 ^{1}H NMR spectrum of (3-nitrophenyl)(phenyl)methanol recorded in $CDCl_3$

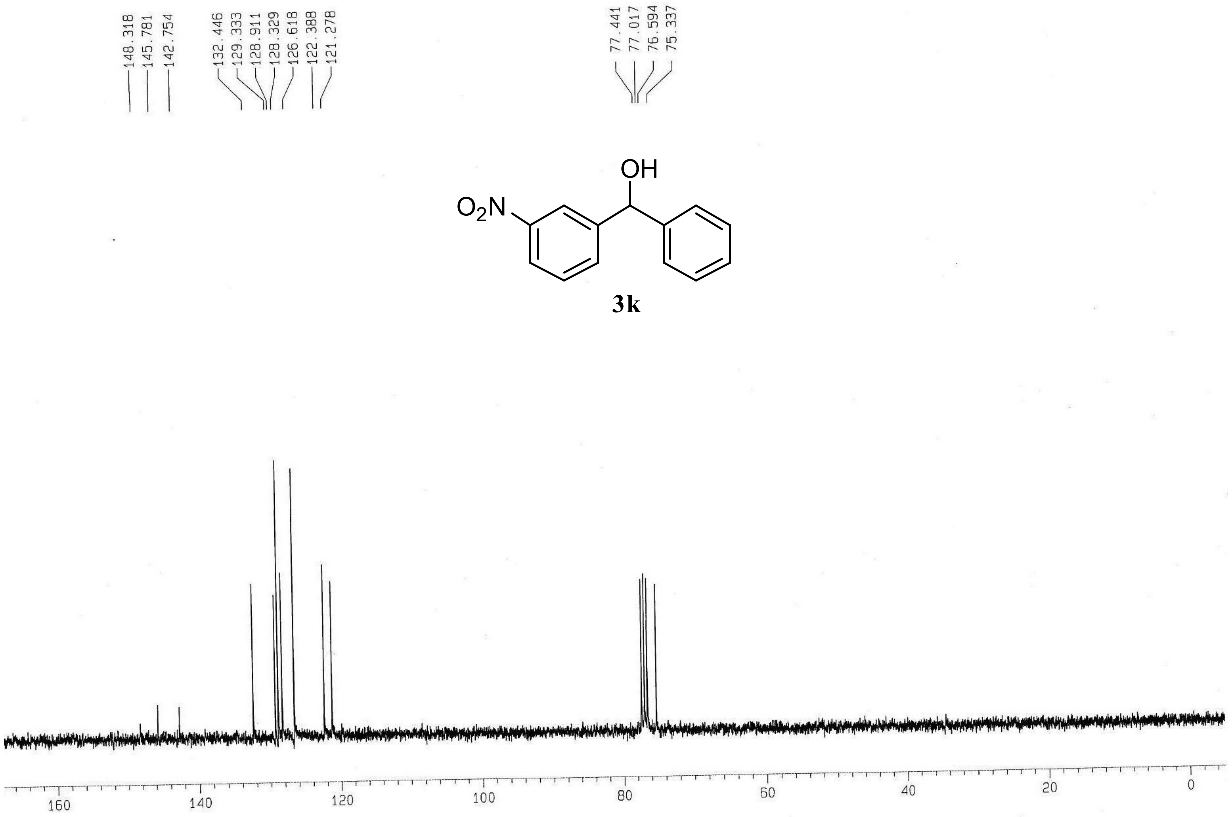

Attached Fig.22 ^{13}C NMR spectrum of (3-nitrophenyl)(phenyl)methanol recorded in $CDCl_3$

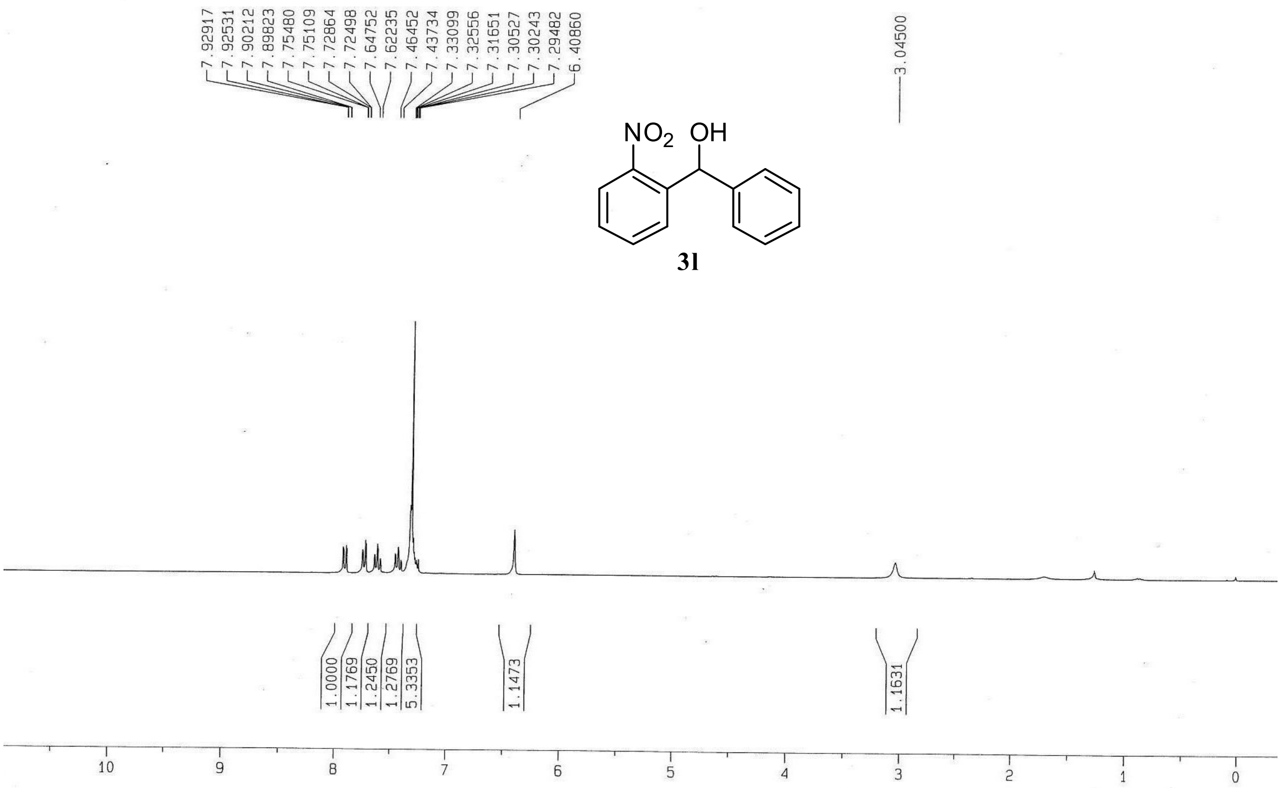

Attached Fig.23 ^{1}H NMR spectrum of (2-nitrophenyl)(phenyl)methanol recorded in $CDCl_3$

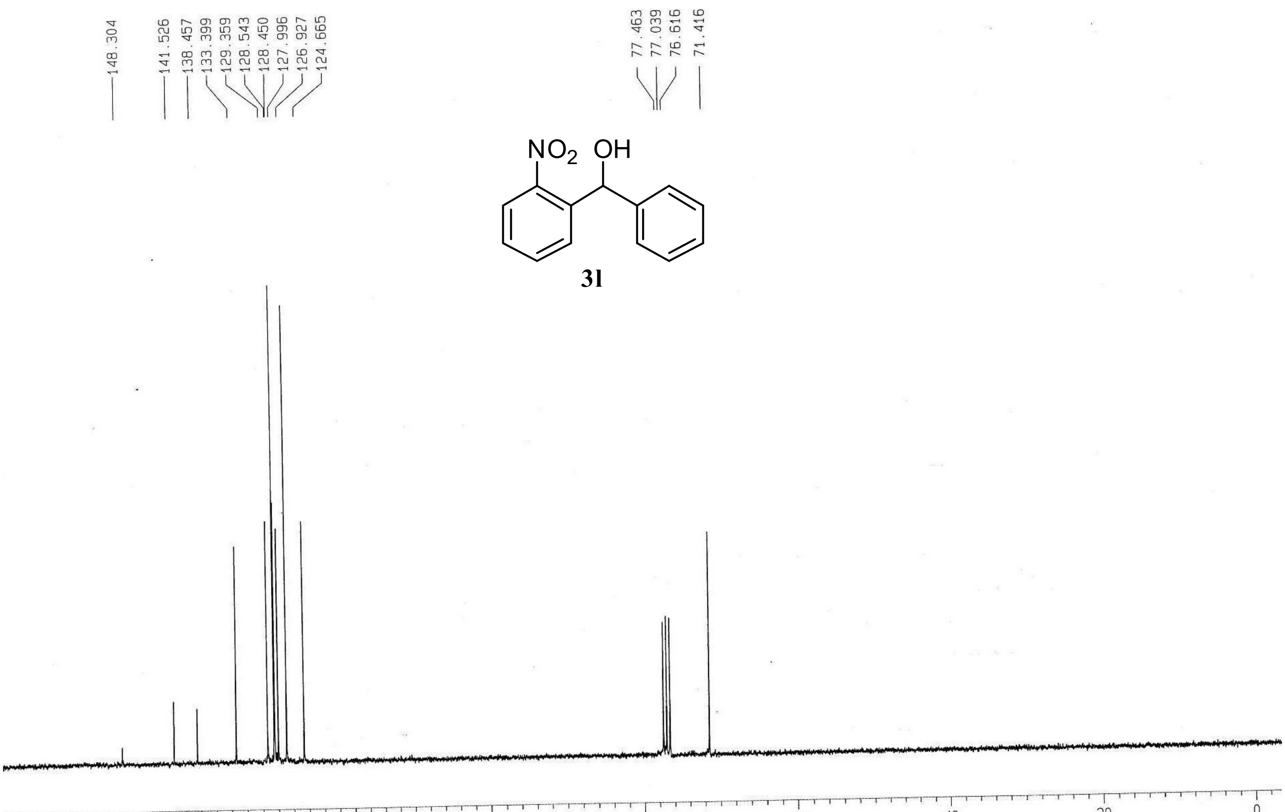

Attached Fig.24 ^{13}C NMR spectrum of (2-nitrophenyl)(phenyl)methanol recorded in $CDCl_3$

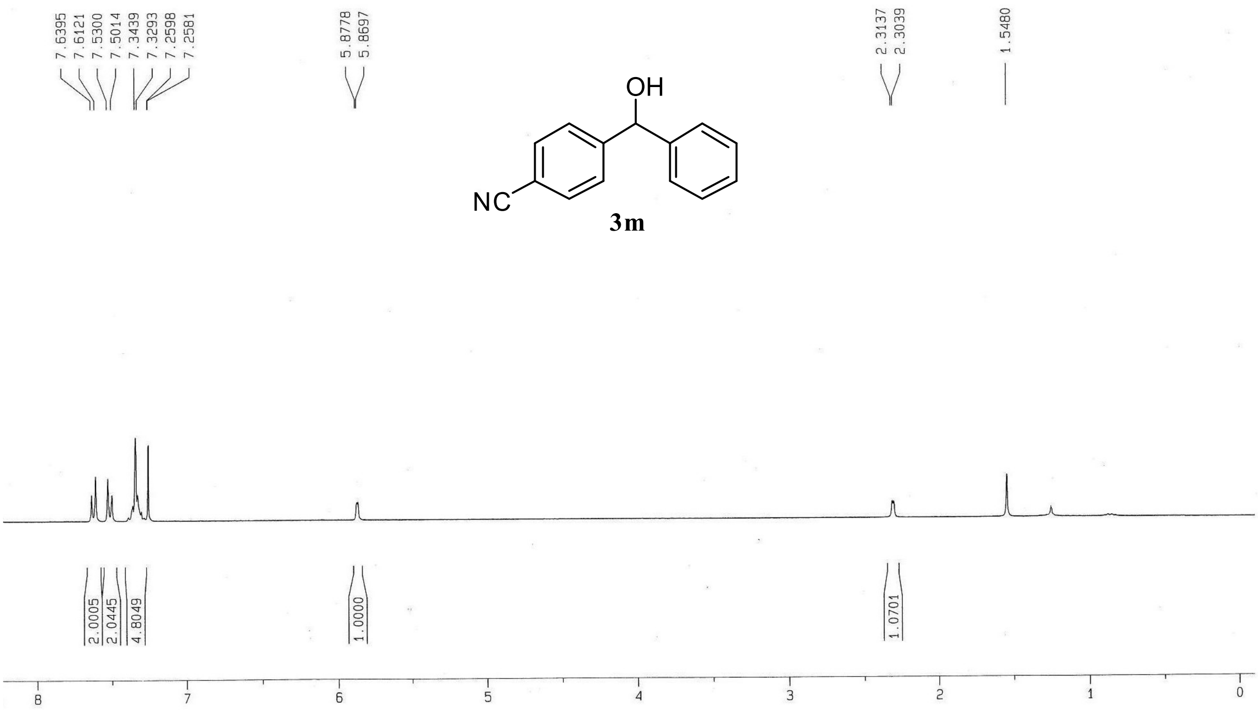

Attached Fig.25 ^{1}H NMR spectrum of 4-(hydroxy(phenyl)methyl)benzonitrile recorded in $CDCl_3$

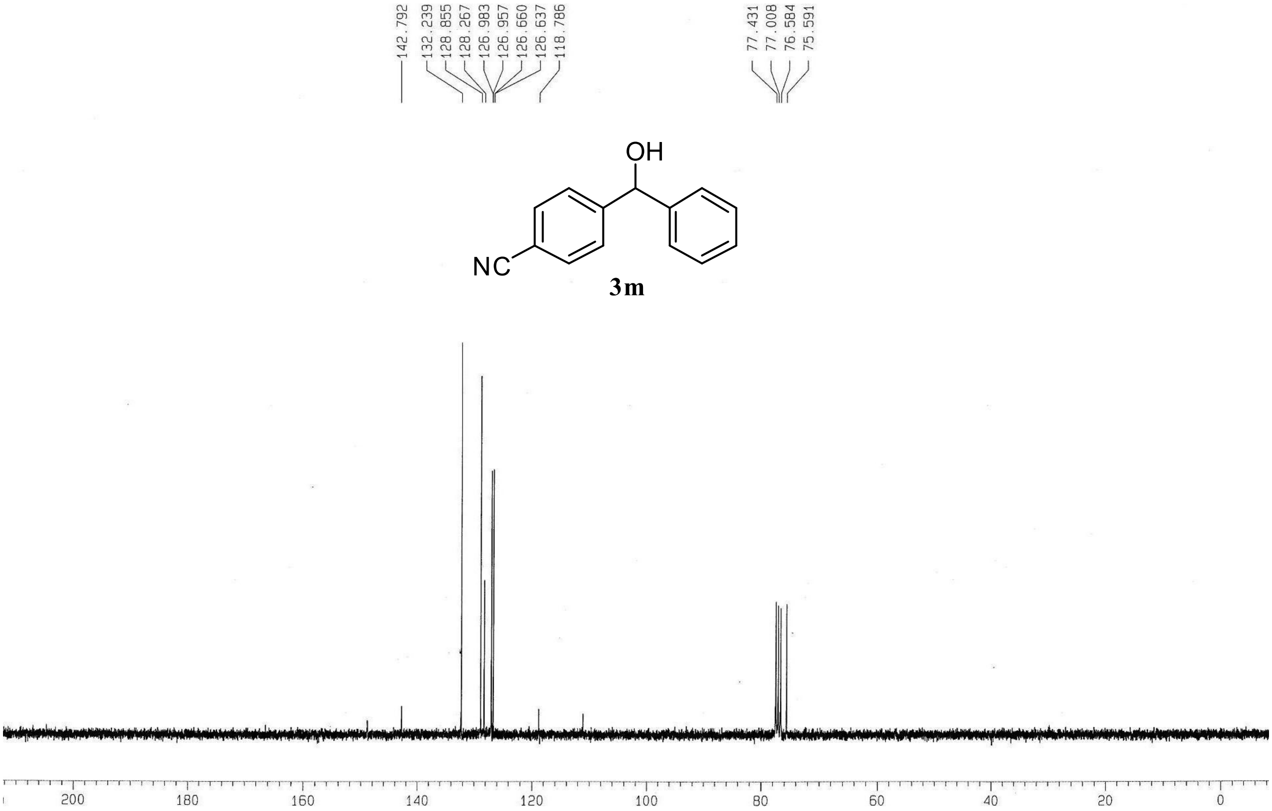

Attached Fig.26 ^{13}C NMR spectrum of 4-(hydroxy(phenyl)methyl)benzonitrile recorded in $CDCl_3$

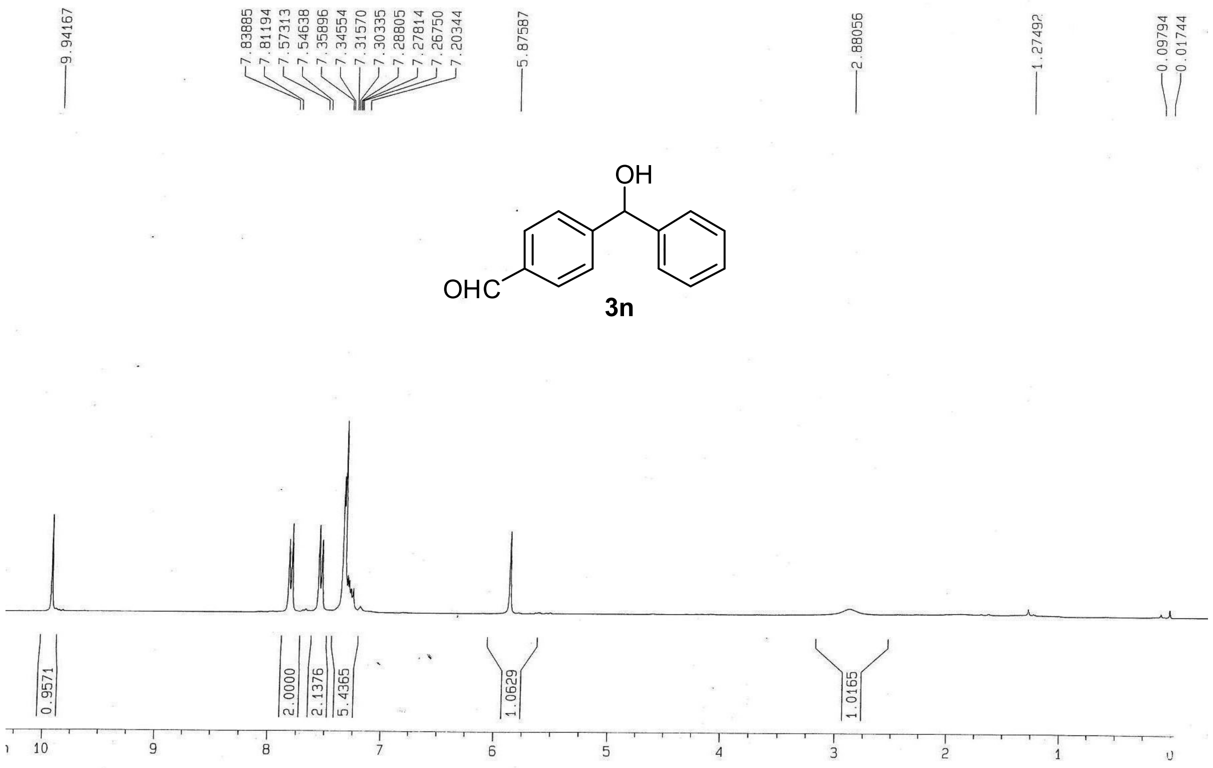

Attached **Fig.27** ^{1}H NMR spectrum of 4-(hydroxy(phenyl)methyl)benzonitrile recorded in $CDCl_3$

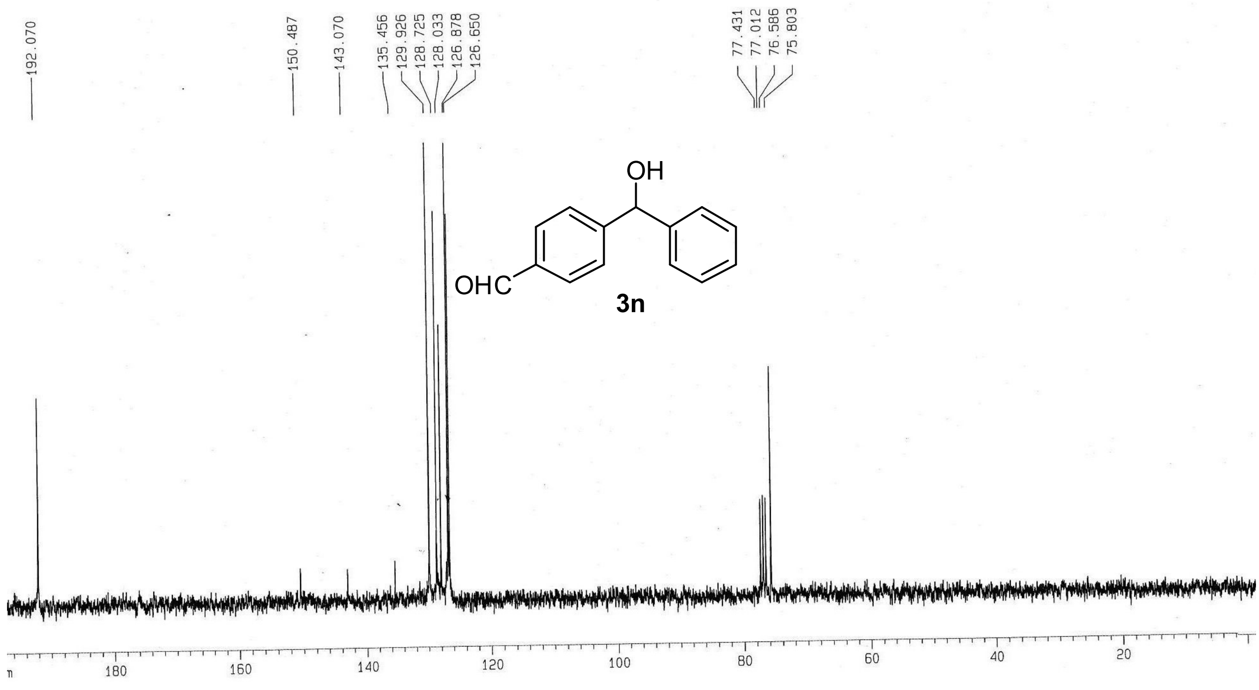

Attached **Fig.28** ^{13}C NMR spectrum of 4-(hydroxy(phenyl)methyl)benzonitrile recorded in $CDCl_3$

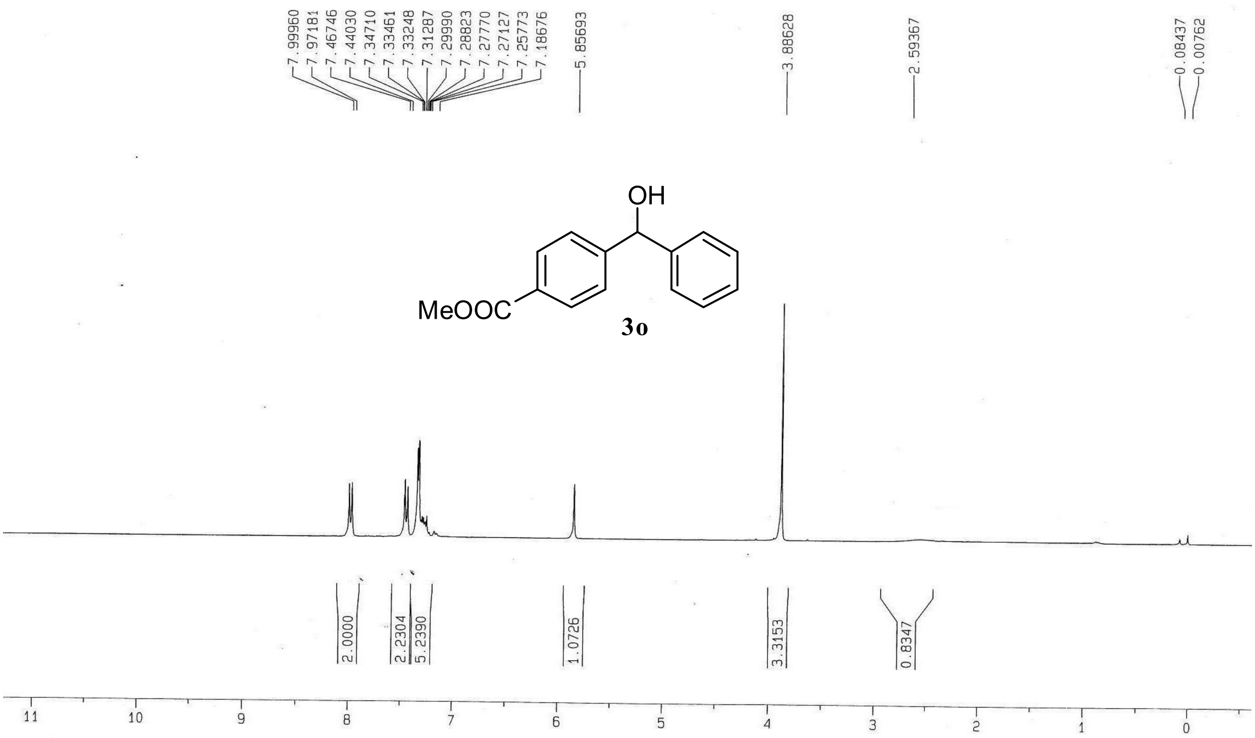

Attached Fig.29 ^{1}H NMR spectrum of 4-(hydroxy(phenyl)methyl)benzonitrile recorded in $CDCl_3$

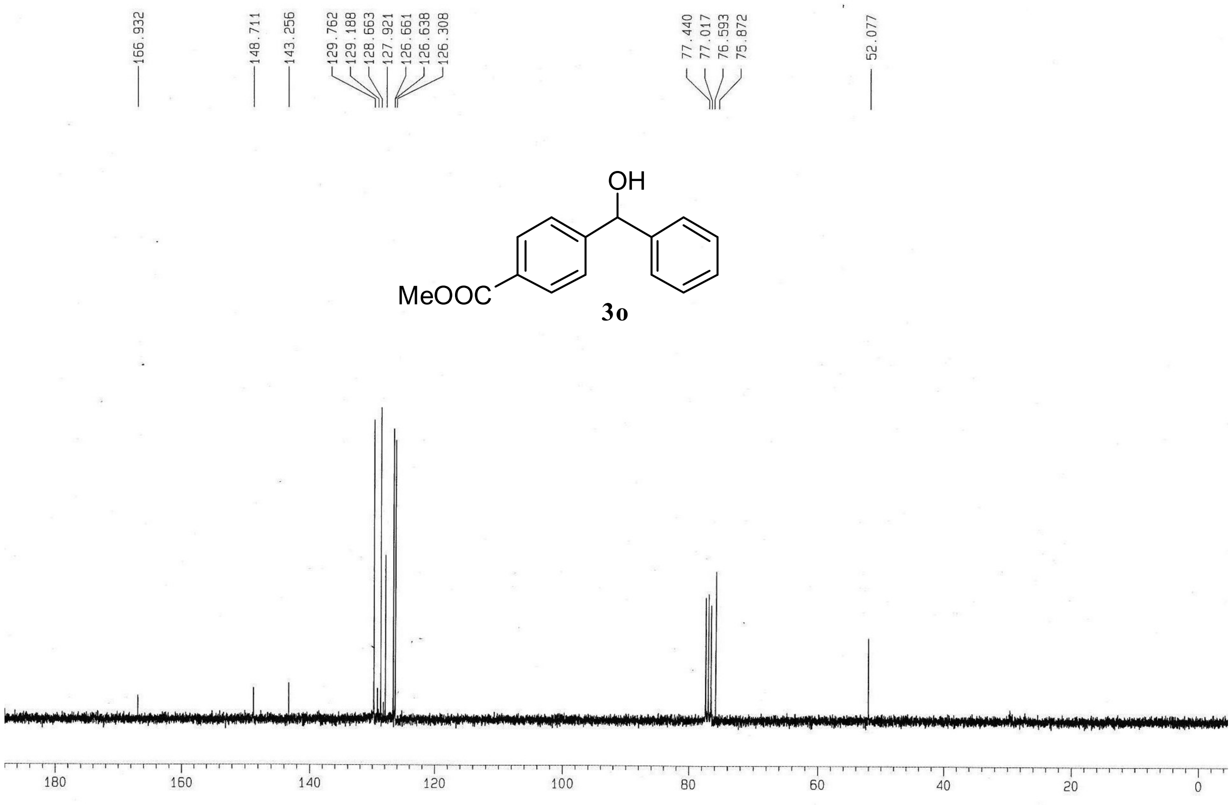

Attached Fig.30 ^{13}C NMR spectrum of 4-(hydroxy(phenyl)methyl)benzonitrile recorded in $CDCl_3$

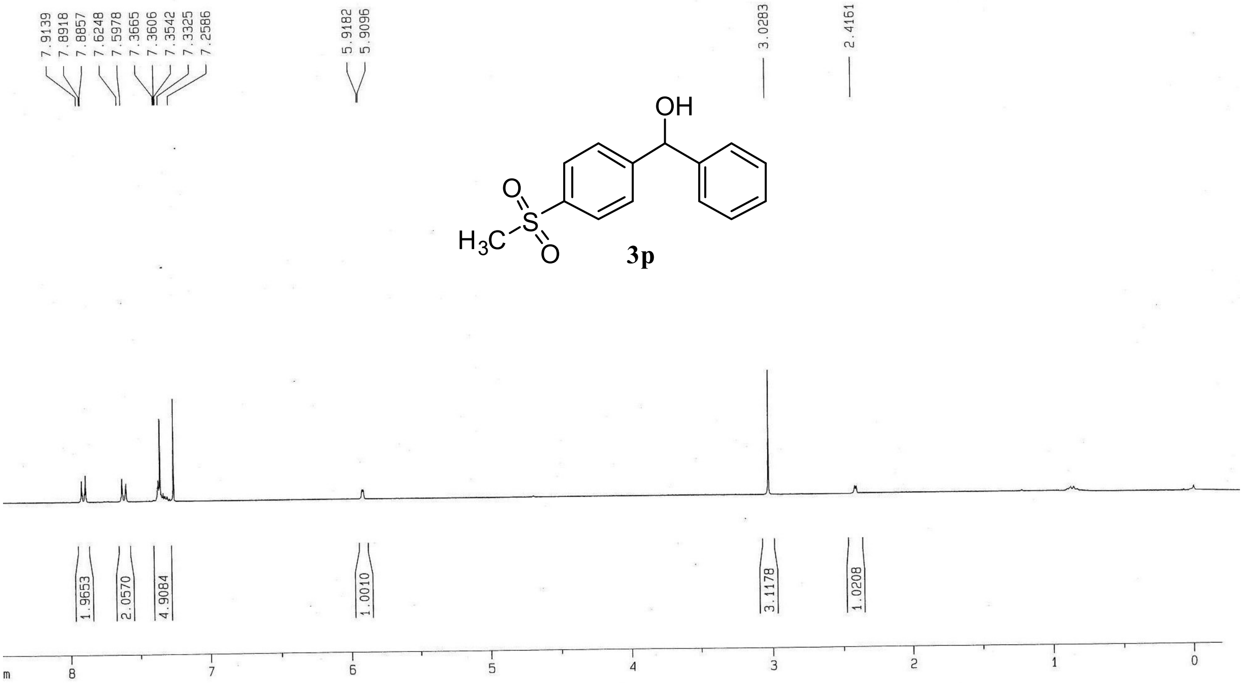

Attached Fig.31 ^{1}H NMR spectrum of 4-(hydroxy(phenyl)methyl)benzonitrile recorded in $CDCl_3$

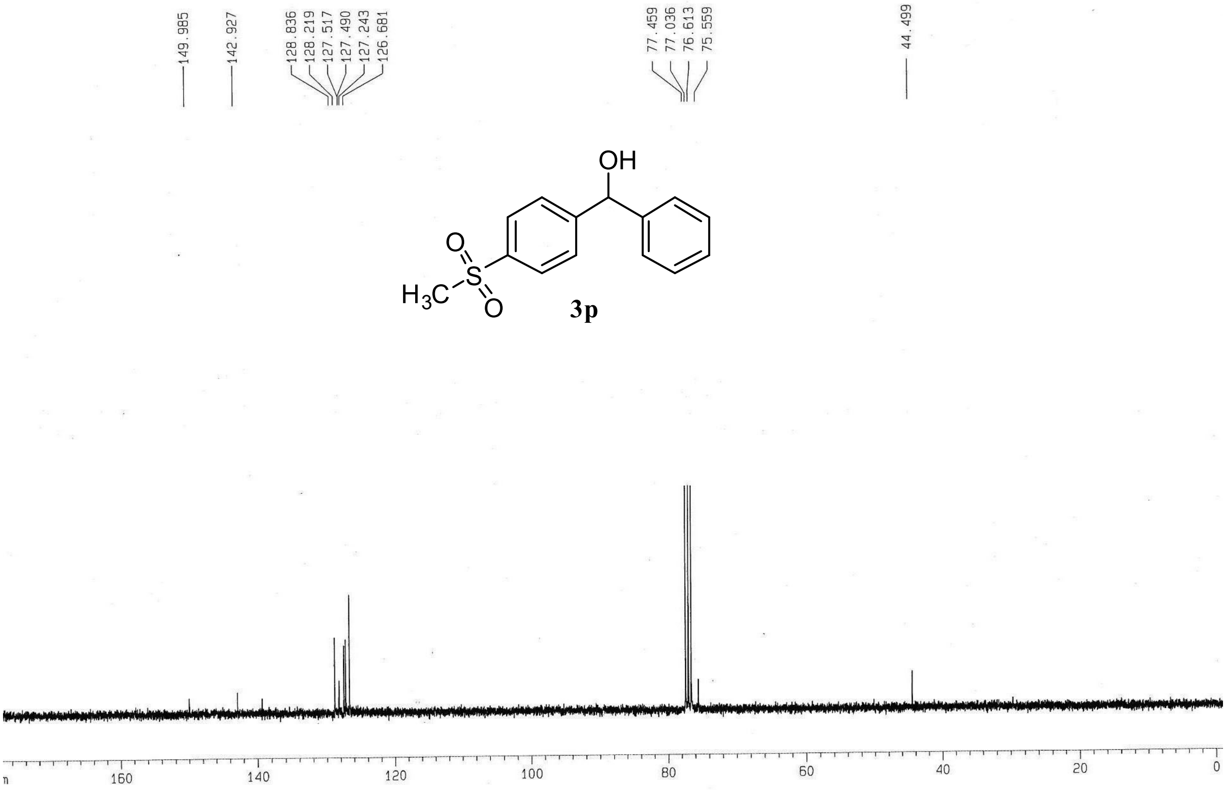

Attached Fig.32 ^{13}C NMR spectrum of 4-(hydroxy(phenyl)methyl)benzonitrile recorded in $CDCl_3$

6. The NMR spectra of benzil derivatives

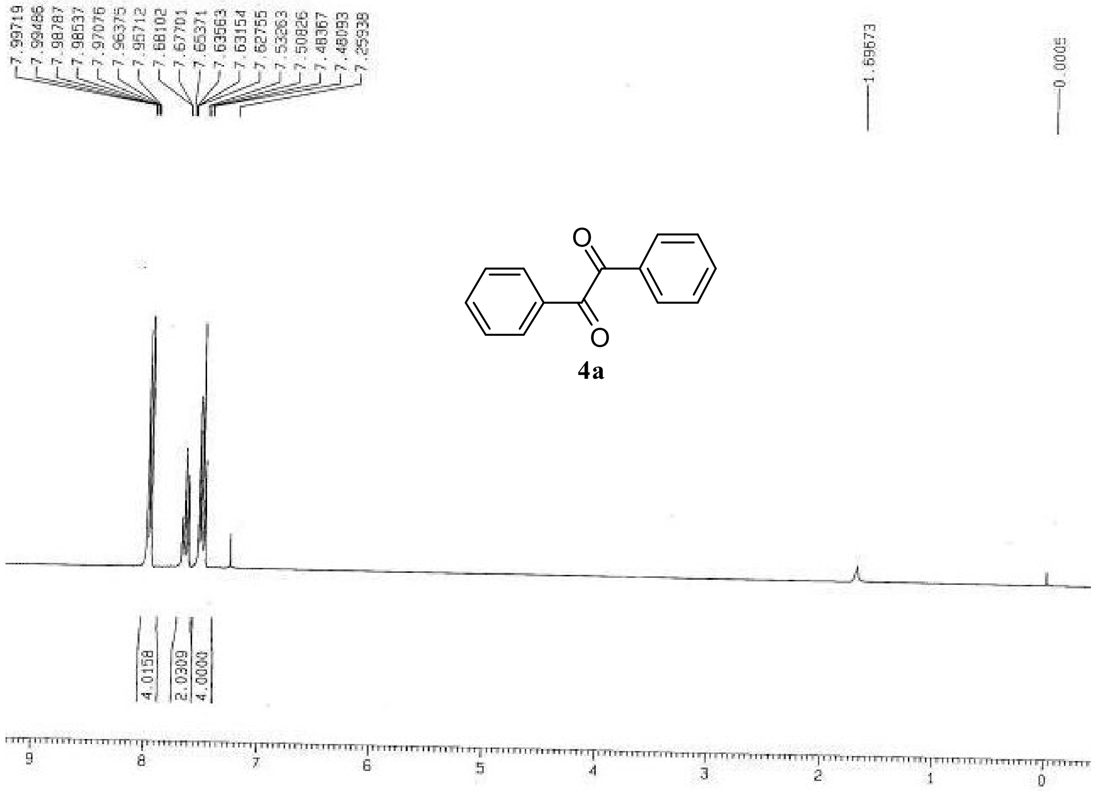

Attached Fig.33 ^{1}H NMR spectrum of benzil recorded in $CDCl_3$

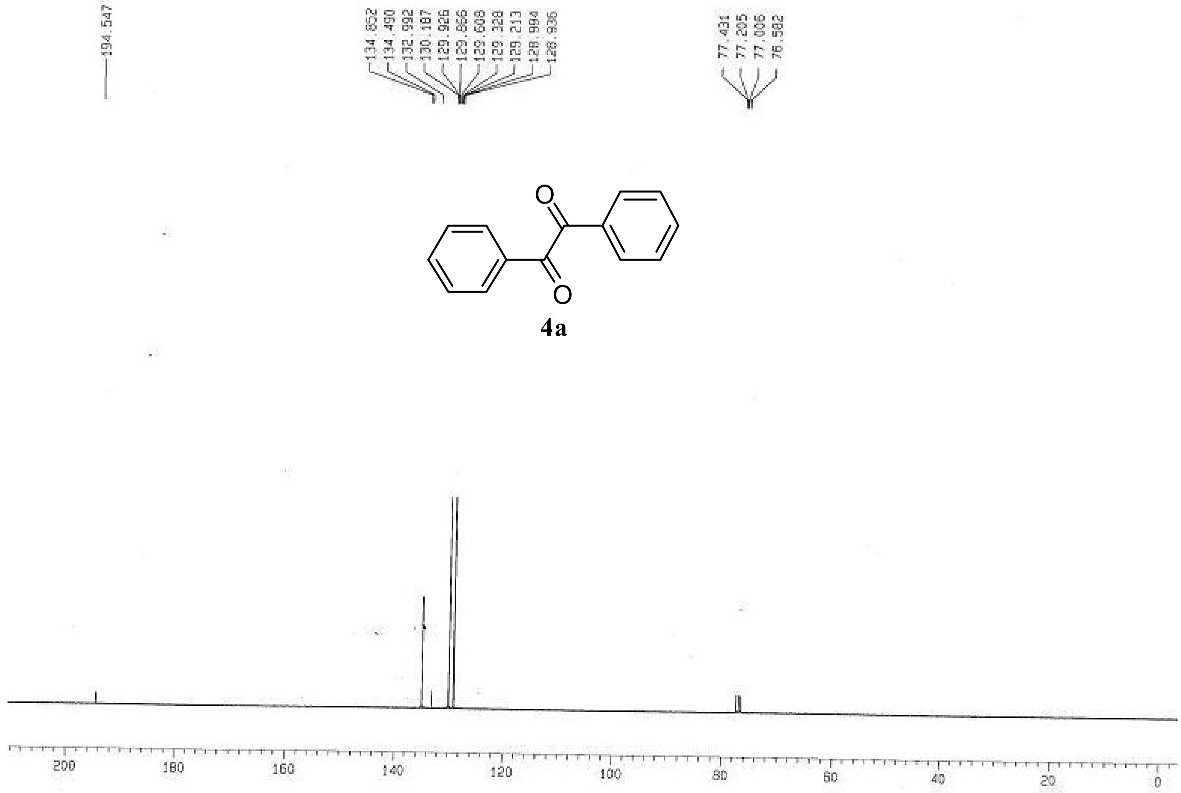

Attached Fig. 34 ^{13}C NMR spectrum of benzil recorded in $CDCl_3$

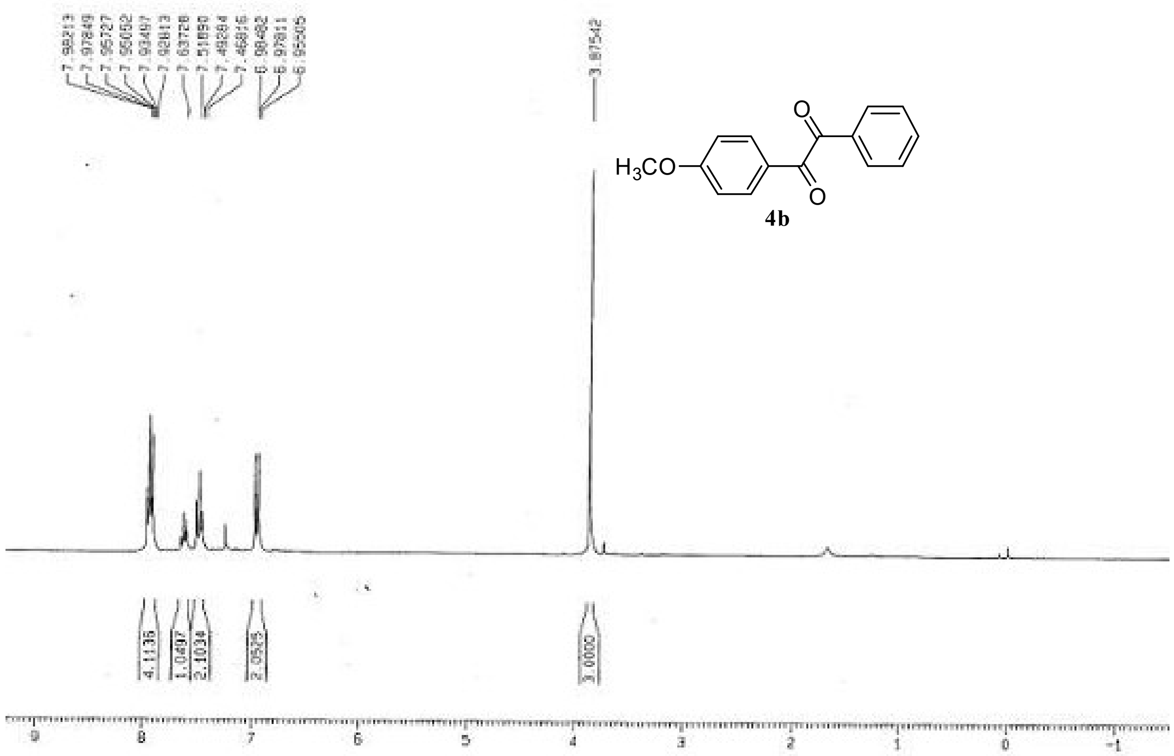

Attached Fig.35 ^{1}H NMR spectrum of 1-(4-methoxyphenyl)-2-phenylethane-1,2-dione recorded in $CDCl_3$

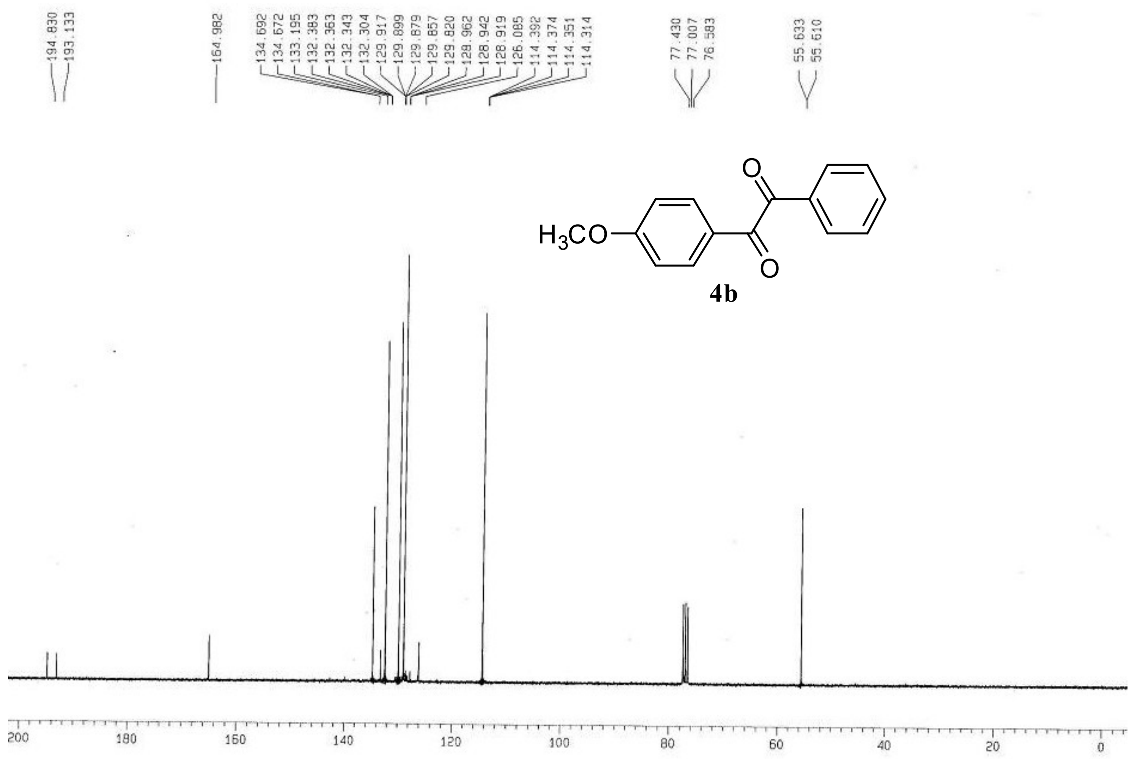

Attached Fig.36 ^{13}C NMR spectrum of 1-(4-methoxyphenyl)-2-phenylethane-1,2-dione recorded in $CDCl_3$

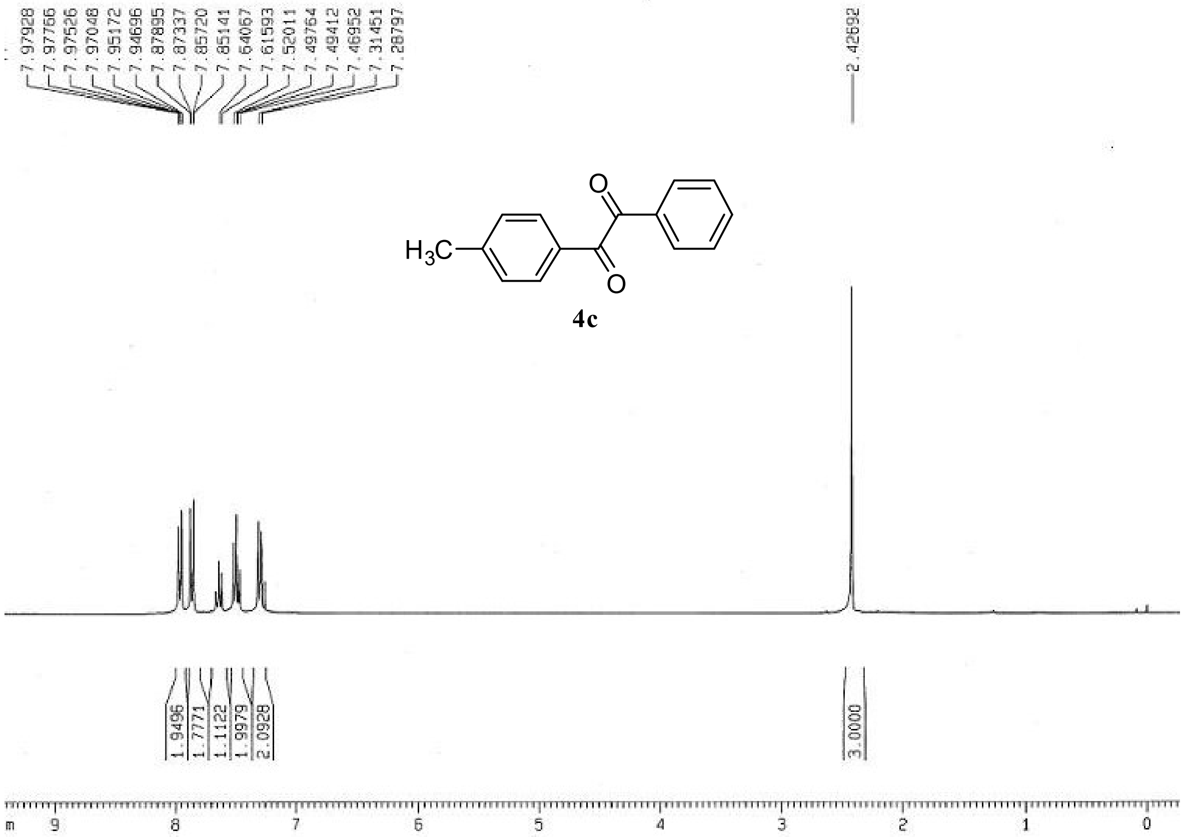

Attached Fig.37 ^{1}H NMR spectrum of 1-phenyl-2-(p-tolyl)ethane-1,2-dione recorded in $CDCl_3$

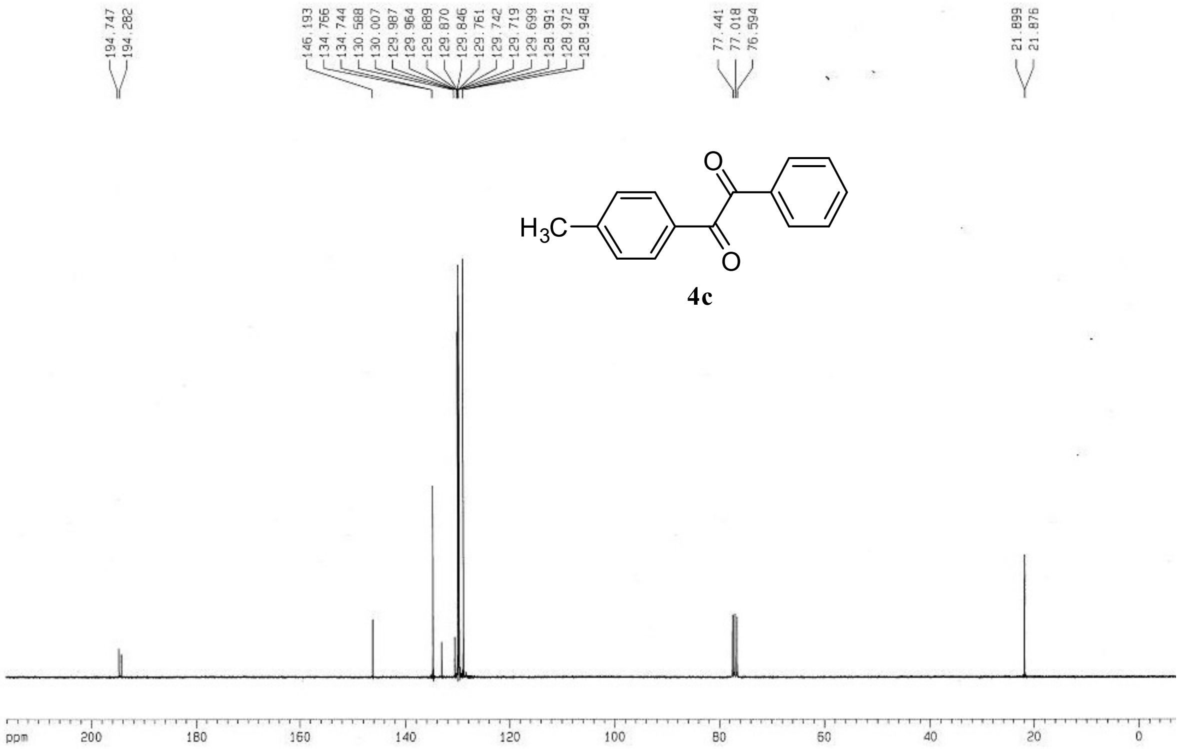

Attached Fig.38 ^{1}H NMR spectrum of 1-(4-fluorophenyl)-2-phenylethane-1,2-dione recorded in $CDCl_3$

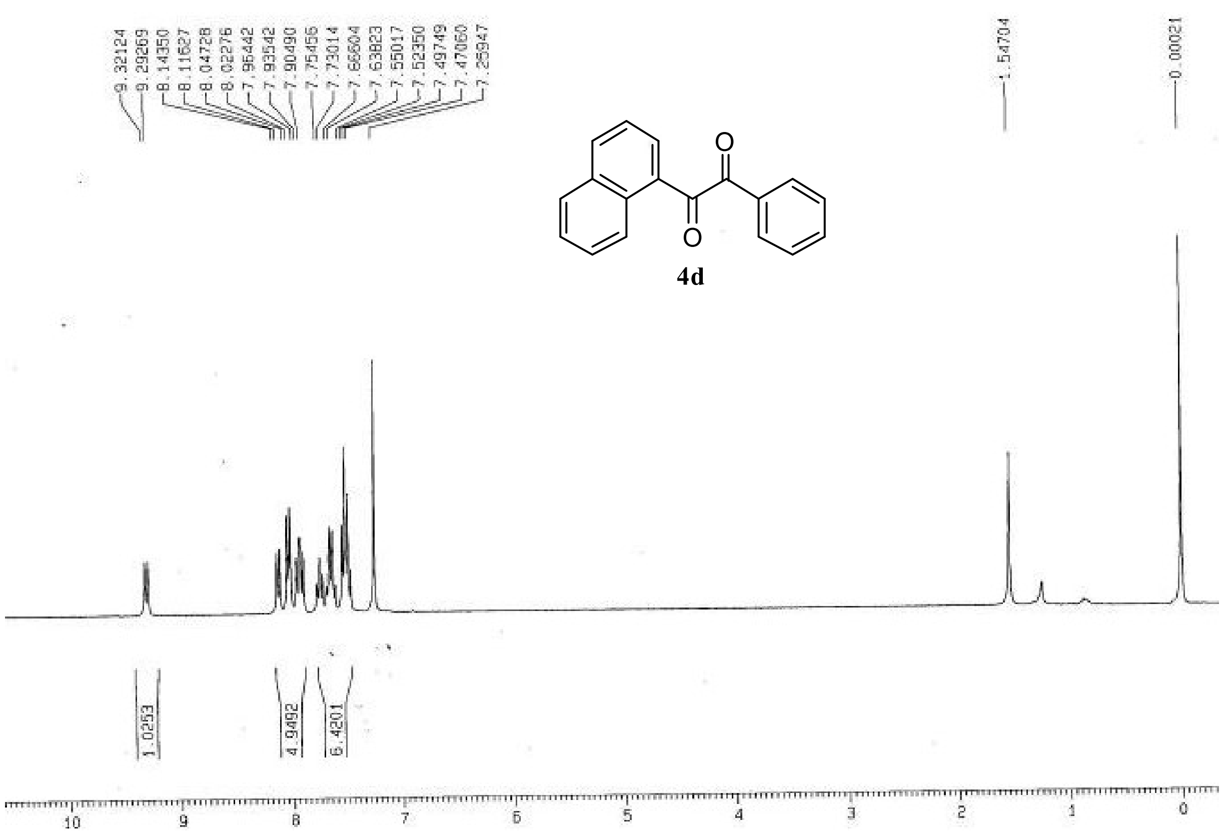

Attached Fig.39 ^{1}H NMR spectrum of 1-(naphthalen-1-yl)-2-phenylethane-1,2-dione recorded in $CDCl_3$

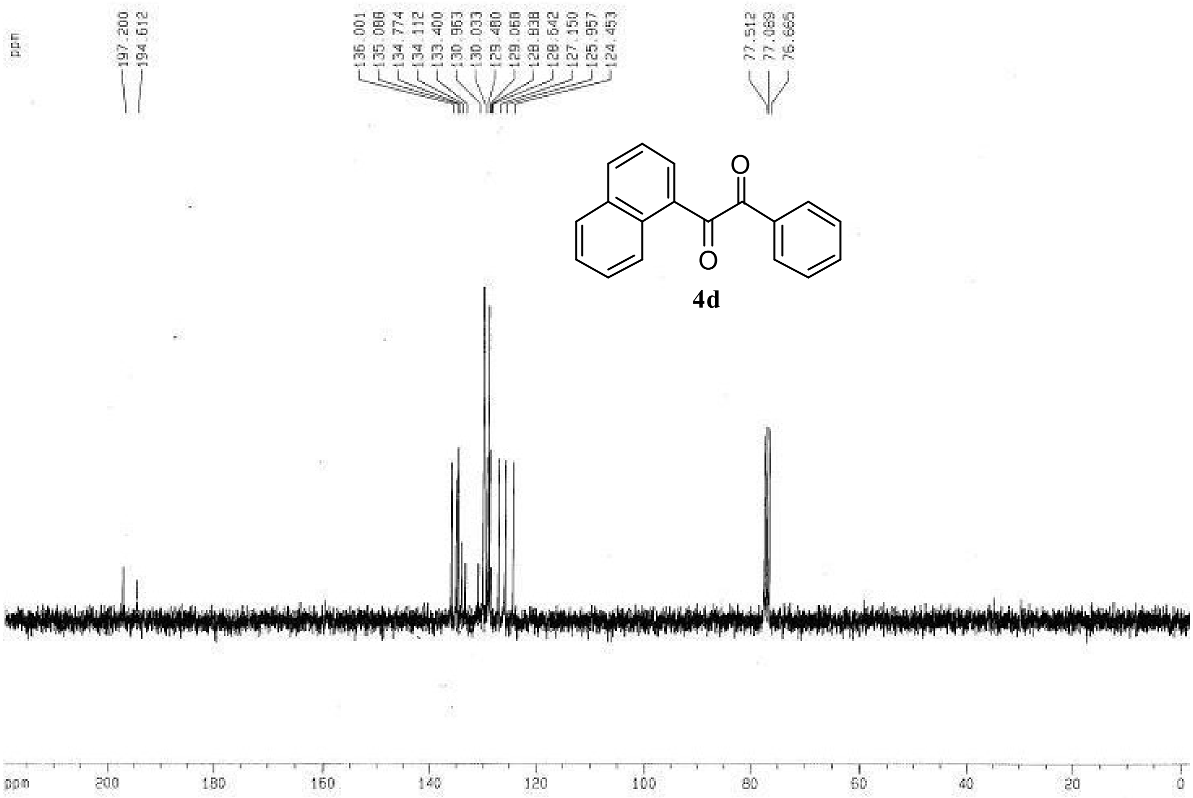

Attached Fig.40 ^{13}C NMR spectrum of 1-(naphthalen-1-yl)-2-phenylethane-1,2-dione recorded in $CDCl_3$

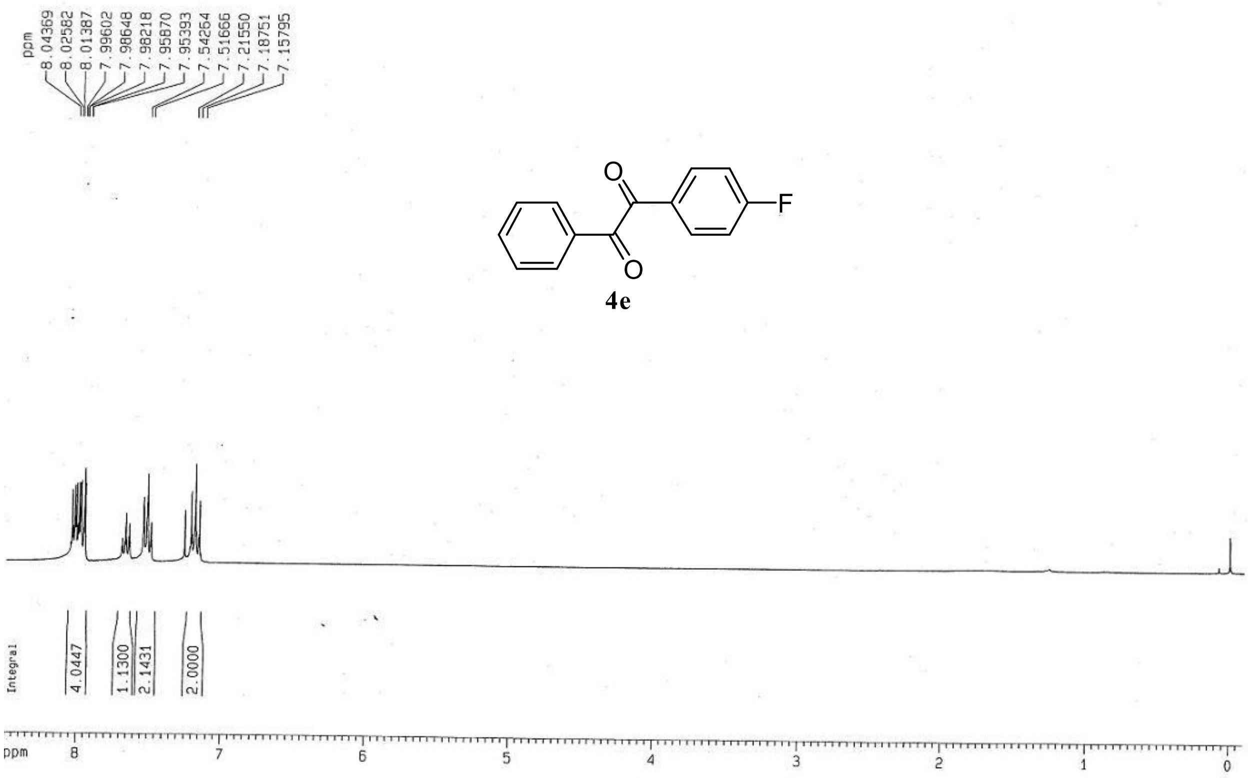

Attached Fig.41 ^{1}H NMR spectrum of 1-(4-fluorophenyl)-2-phenylethane-1,2-dione recorded in $CDCl_3$

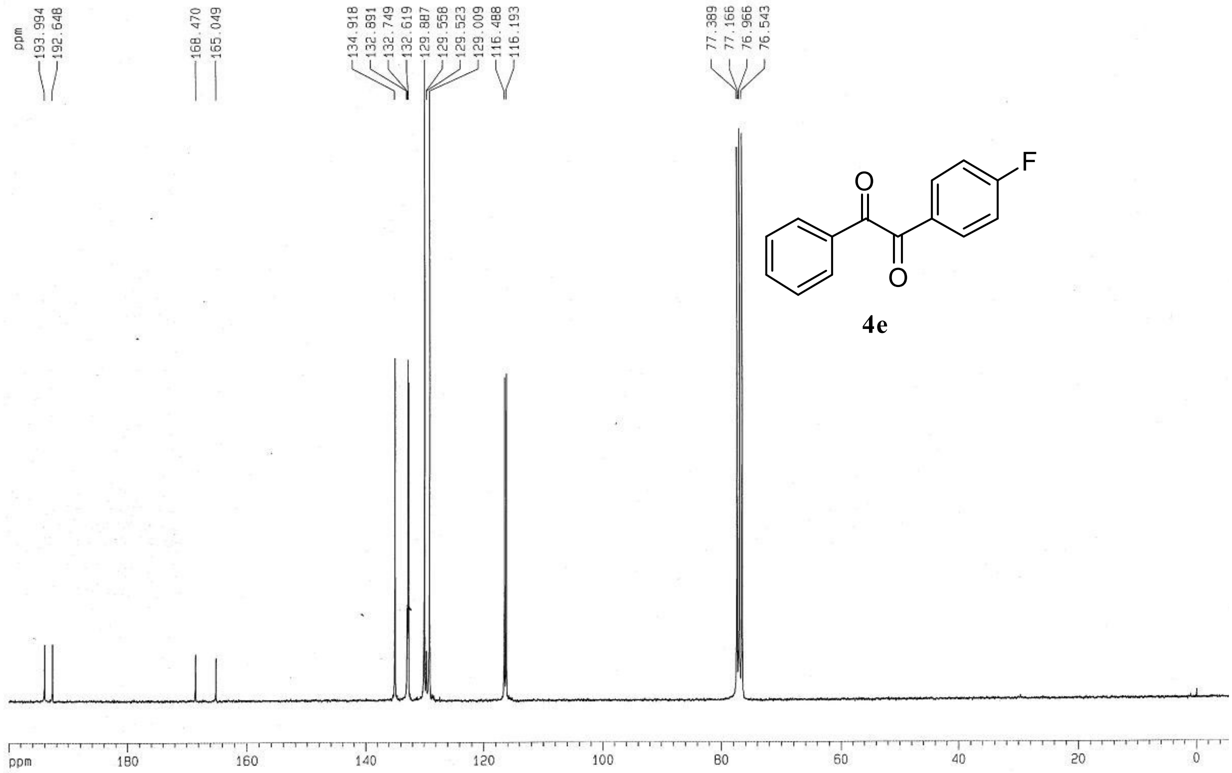

Attached Fig.42 ^{13}C NMR spectrum of 1-(4-fluorophenyl)-2-phenylethane-1,2-dione recorded in $CDCl_3$

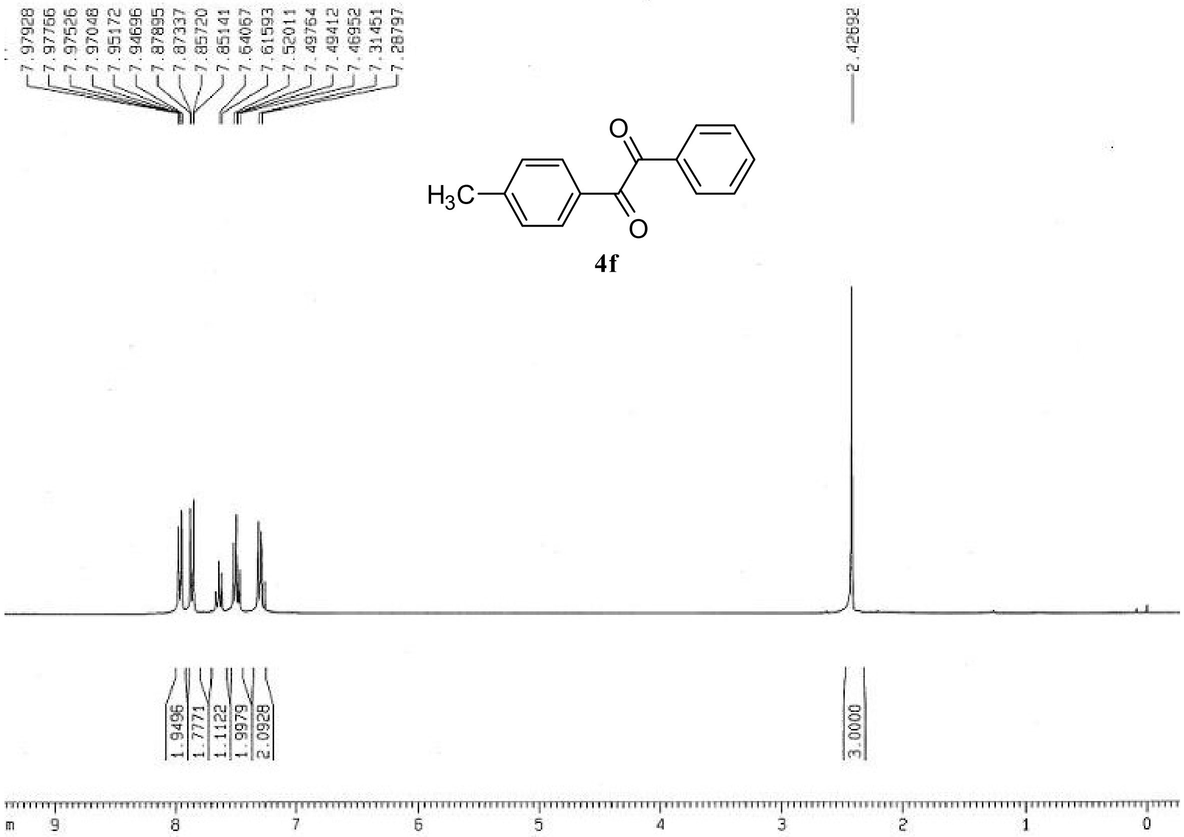

Attached Fig.43 ^{1}H NMR spectrum of 1-phenyl-2-(p-tolyl)ethane-1,2-dione recorded in $CDCl_3$

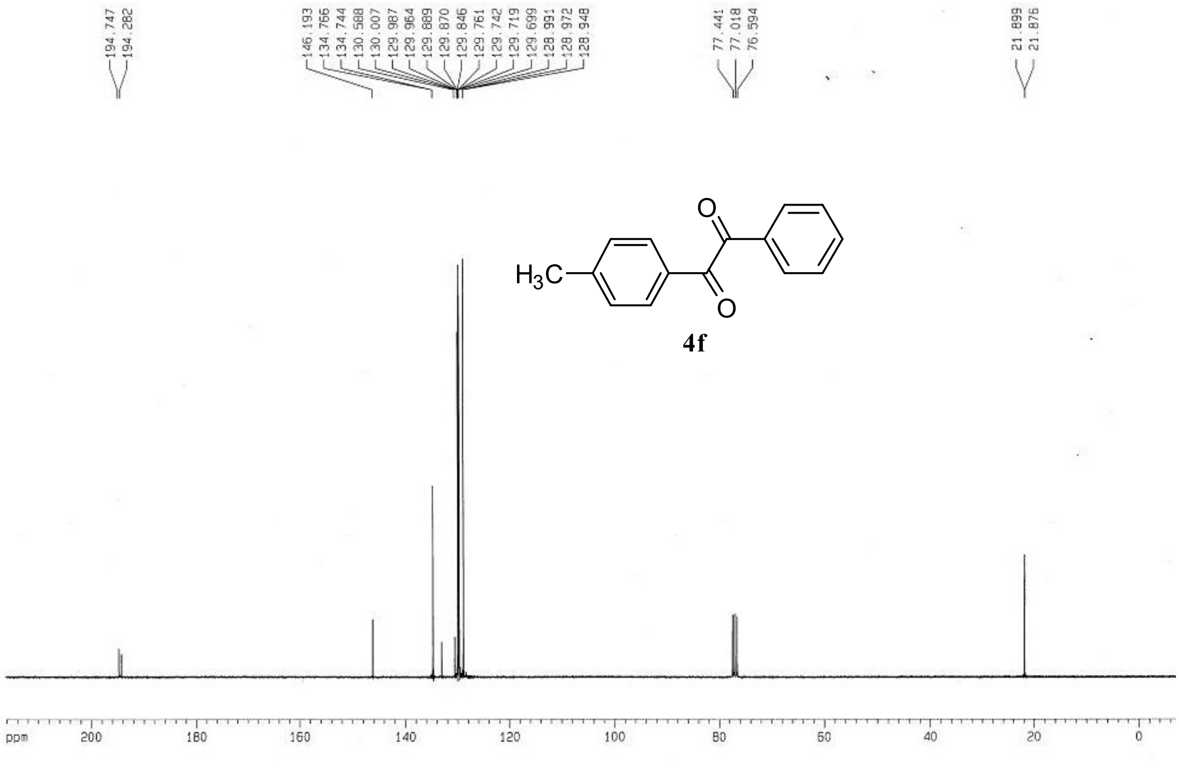

Attached Fig.44 ^{1}H NMR spectrum of 1-(4-fluorophenyl)-2-phenylethane-1,2-dione recorded in $CDCl_3$

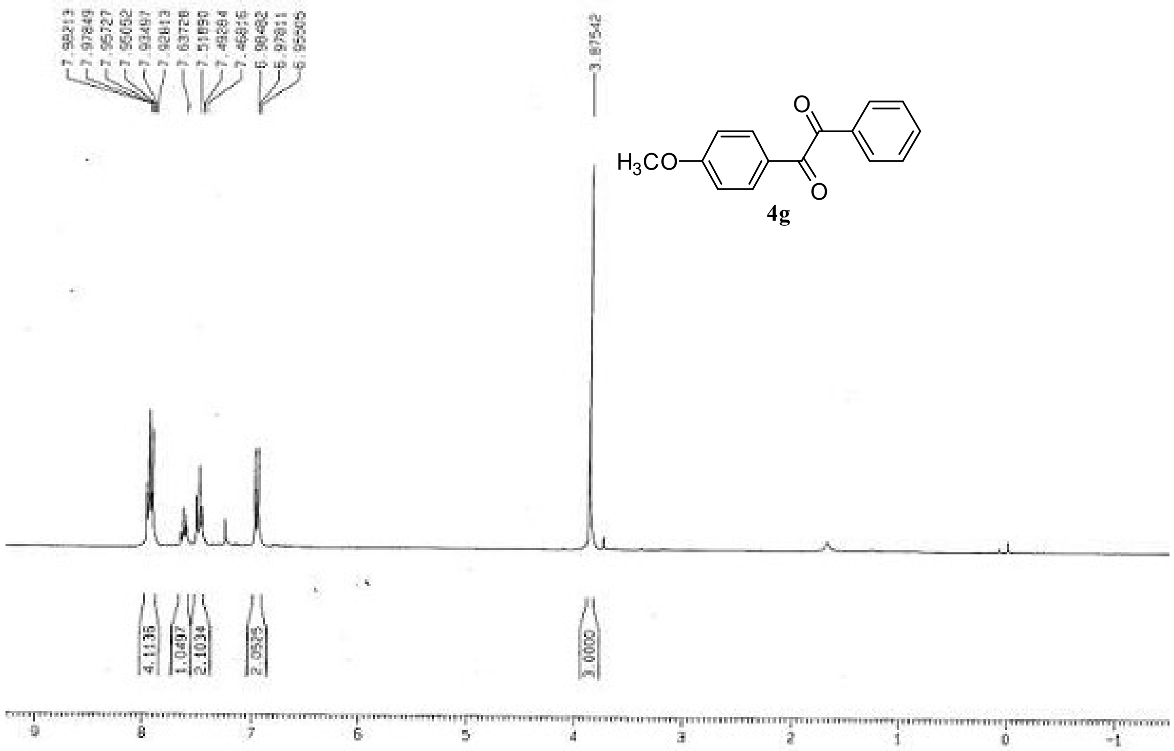

Attached Fig.45 ^{1}H NMR spectrum of 1-(4-methoxyphenyl)-2-phenylethane-1,2-dione recorded in $CDCl_3$

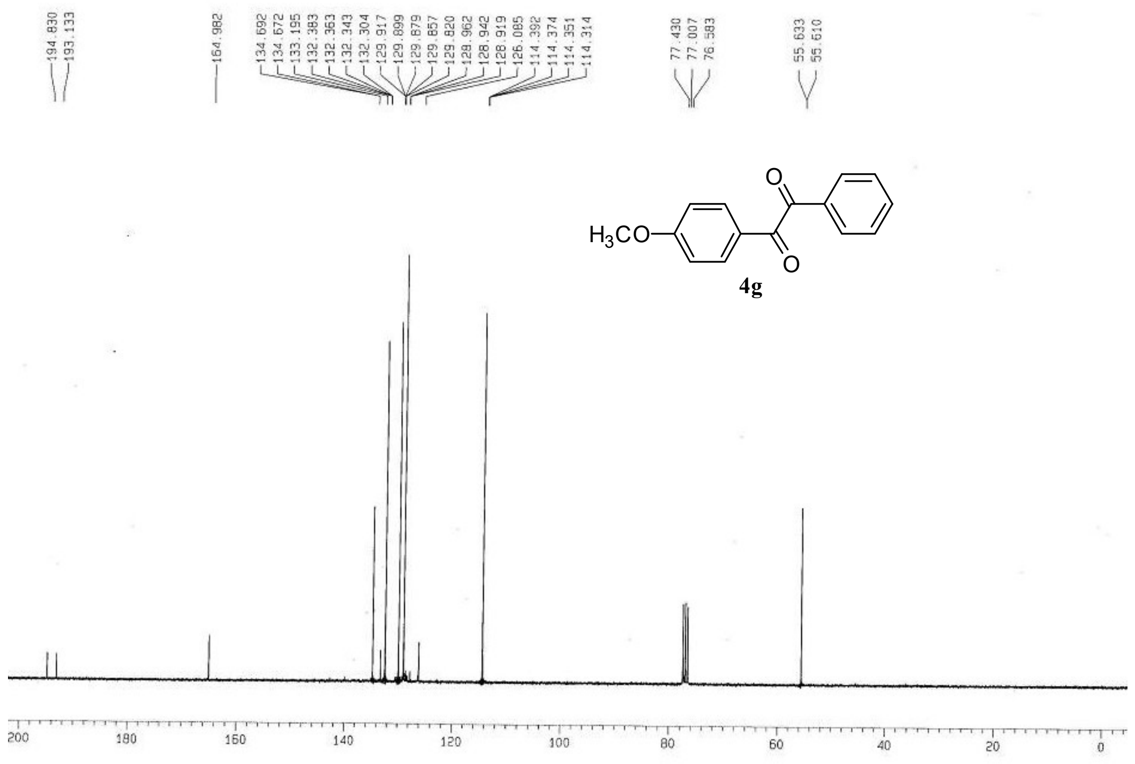

Attached Fig.46 ^{13}C NMR spectrum of 1-(4-methoxyphenyl)-2-phenylethane-1,2-dione recorded in $CDCl_3$

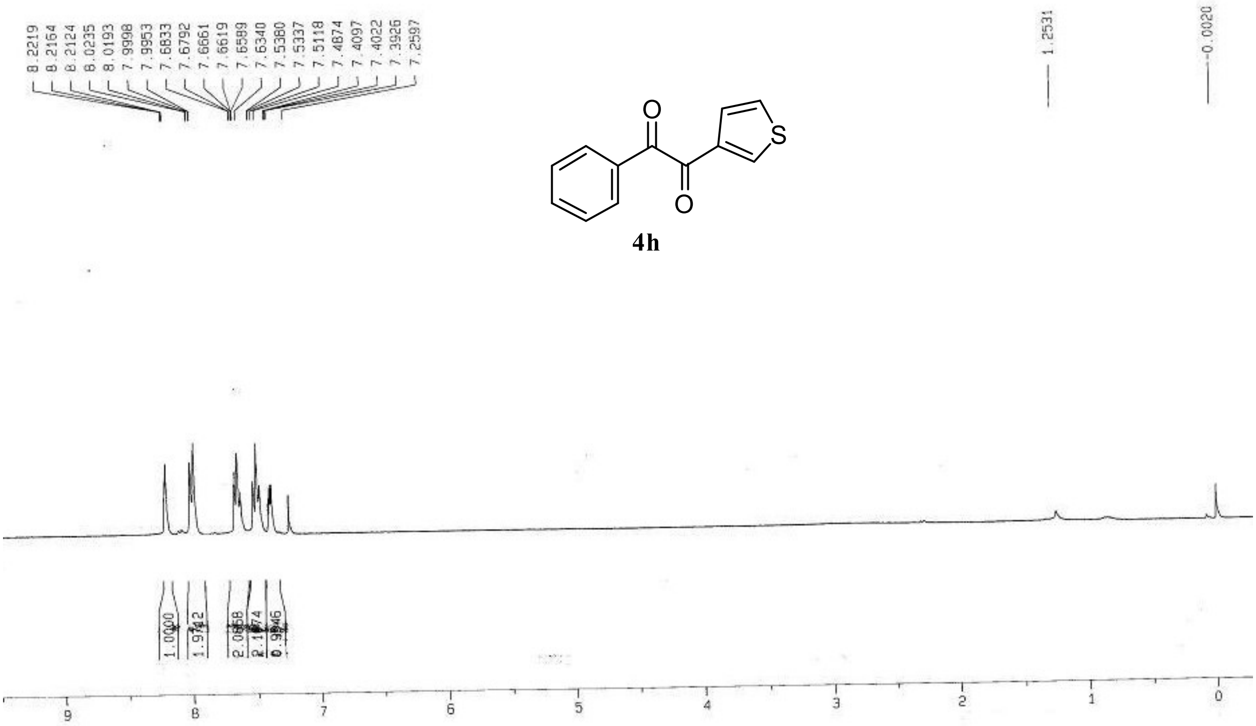

Attached Fig.47 ^{1}H NMR spectrum of 1-phenyl-2-(thiophen-3-yl)ethane-1,2-dione recorded in $CDCl_3$

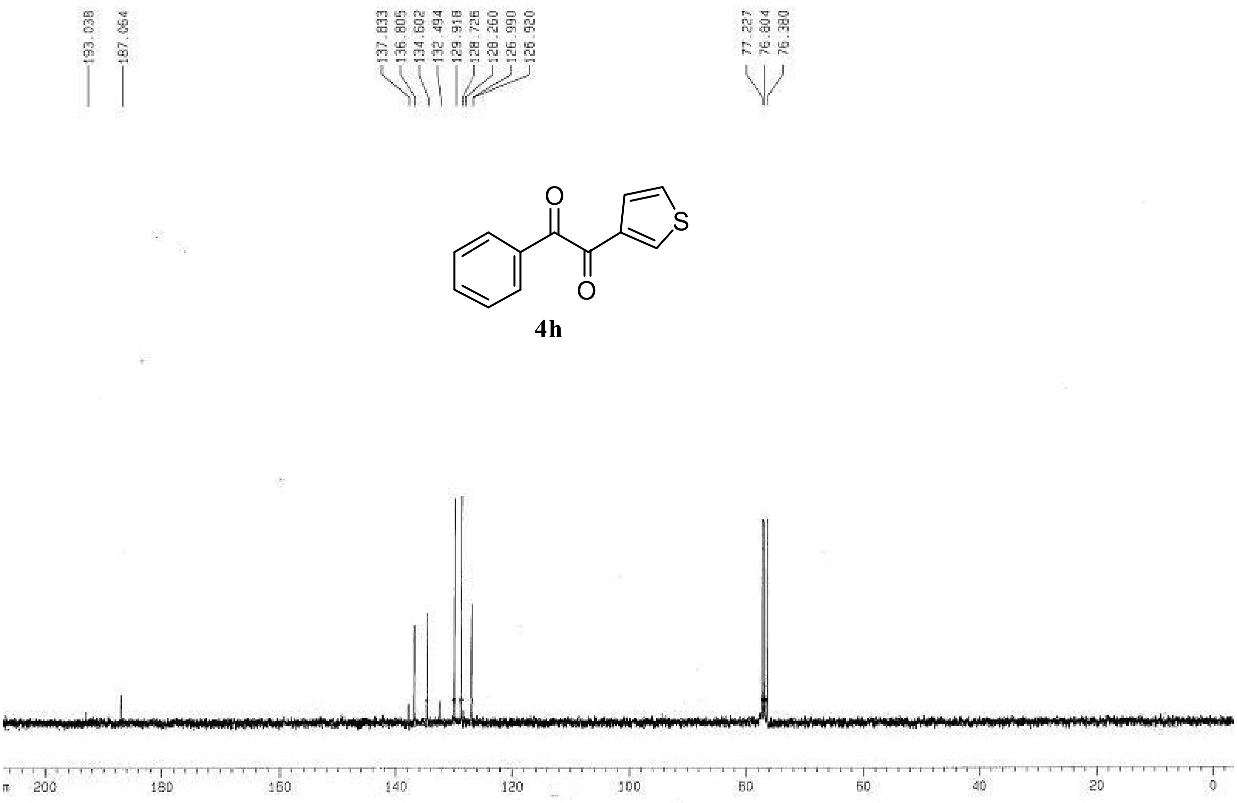

Attached Fig.48 ^{13}C NMR spectrum of 1-phenyl-2-(thiophen-3-yl)ethane-1,2-dione recorded in $CDCl_3$

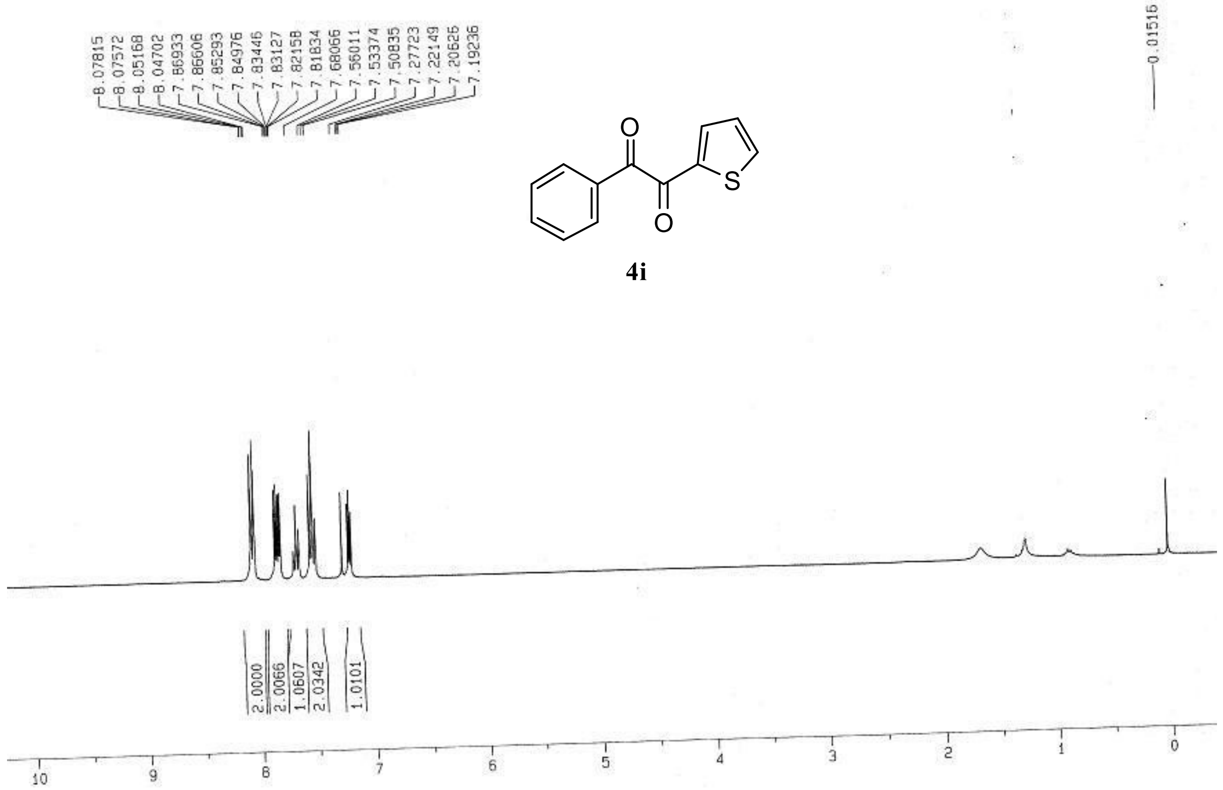

Attached Fig.49 ^{1}H NMR spectrum of 1-phenyl-2-(thiophen-2-yl)ethane-1,2-dione recorded in $CDCl_3$

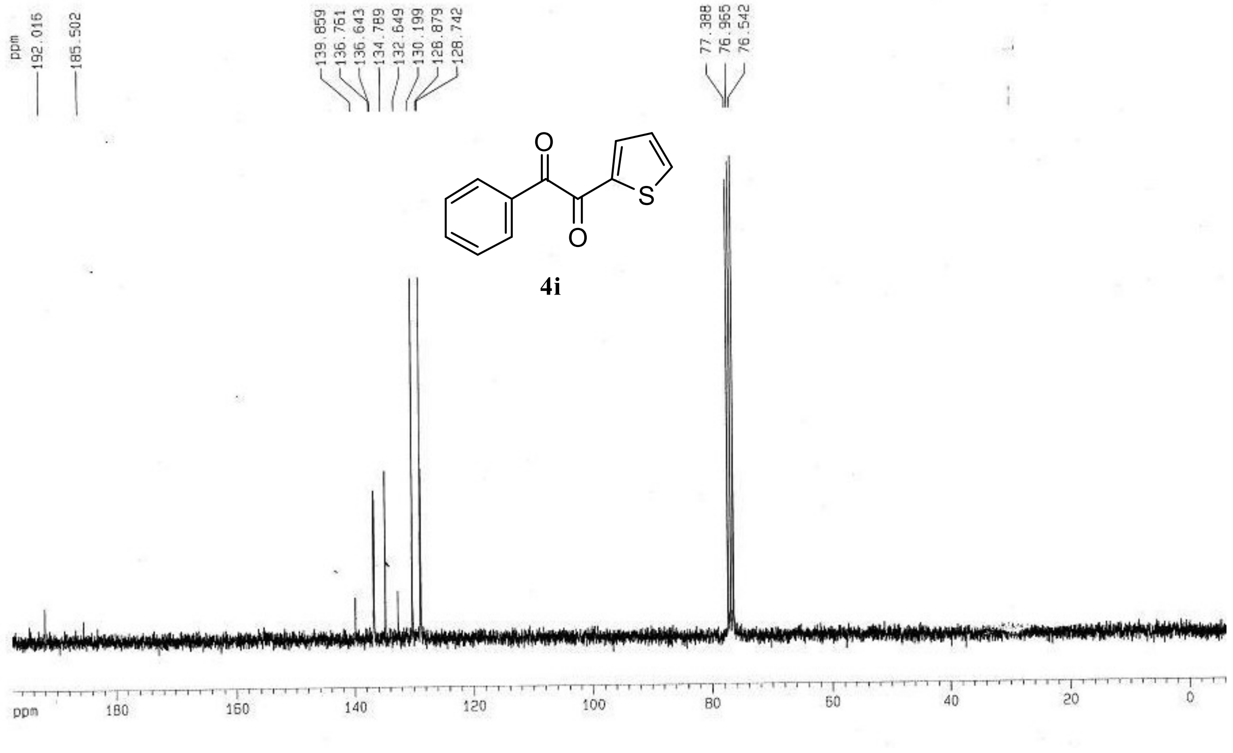

Attached Fig.50 ^{13}C NMR spectrum of 1-phenyl-2-(thiophen-2-yl)ethane-1,2-dione recorded in $CDCl_3$

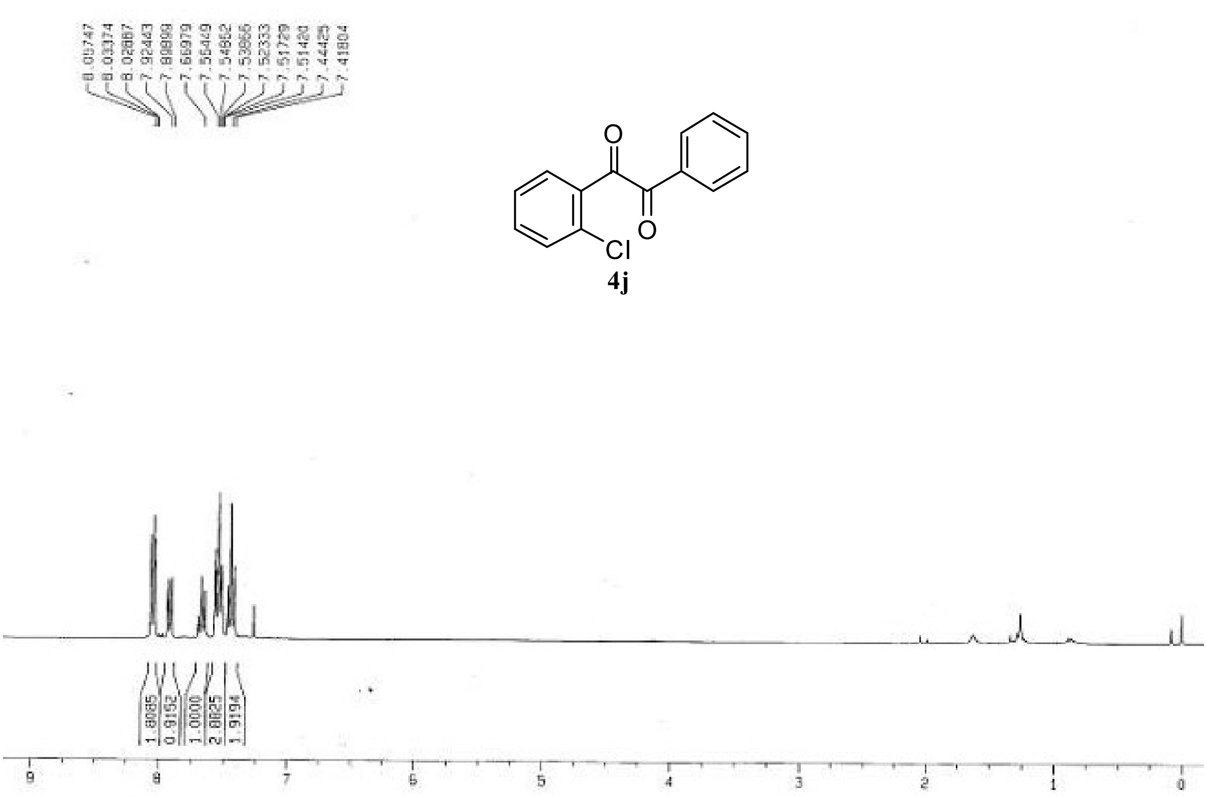

Attached Fig.51 ^{1}H NMR spectrum of 1-(2-chlorophenyl)-2-phenylethane-1,2-dione recorded in $CDCl_3$

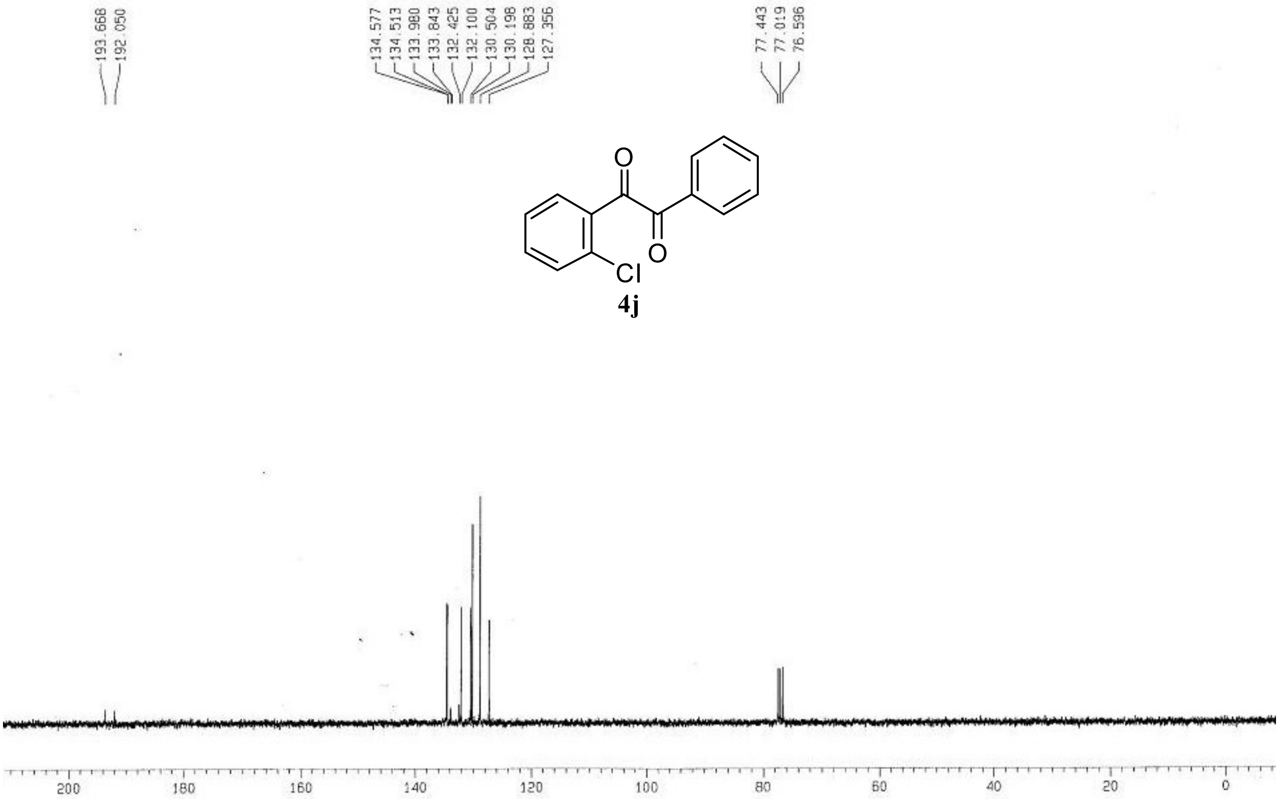

Attached Fig.52 ^{13}C NMR spectrum of 1-(2-chlorophenyl)-2-phenylethane-1,2-dione recorded in $CDCl_3$

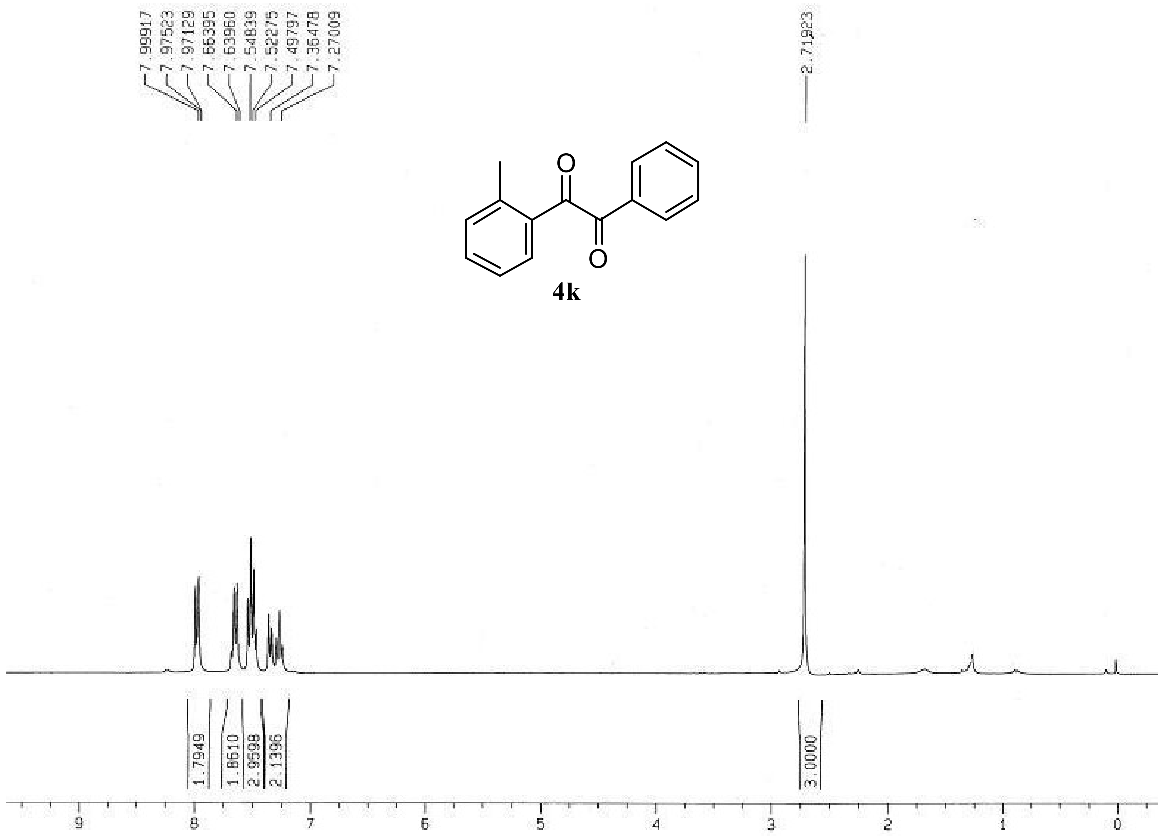

Attached Fig.53 ^{1}H NMR spectrum of 1-phenyl-2-(o-tolyl)ethane-1,2-dione recorded in $CDCl_3$

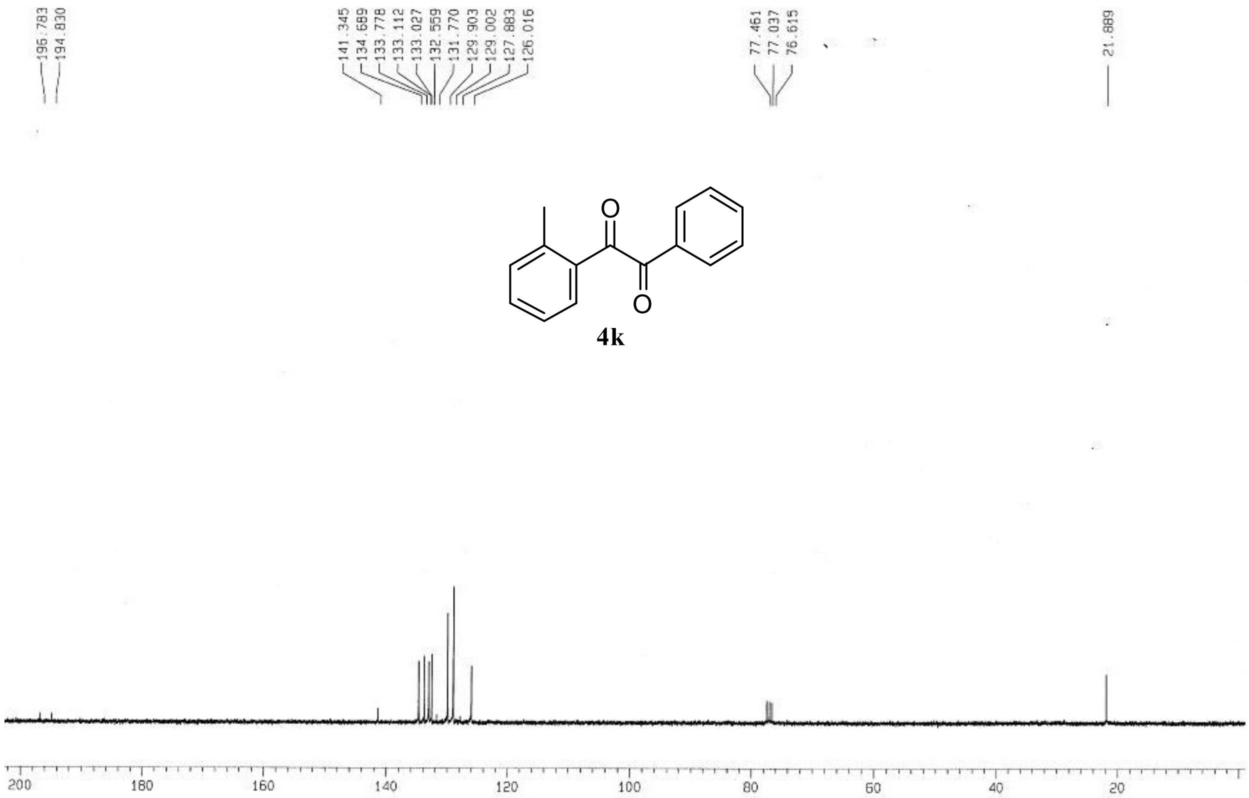

Attached Fig.54 ^{13}C NMR spectrum of 1-phenyl-2-(*o*-tolyl)ethane-1,2-dione recorded in $CDCl_3$

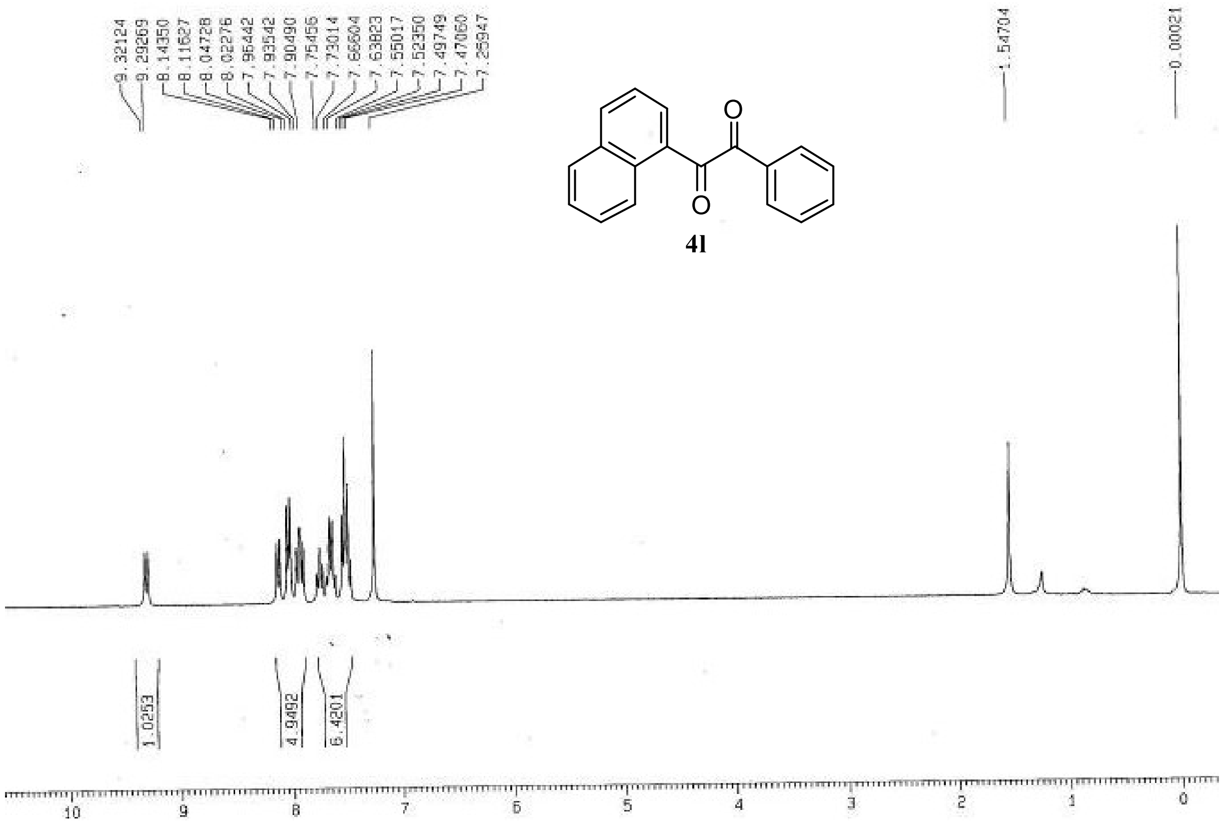

Attached Fig.55 ^{1}H NMR spectrum of 1-(naphthalen-1-yl)-2-phenylethane-1,2-dione recorded in $CDCl_3$

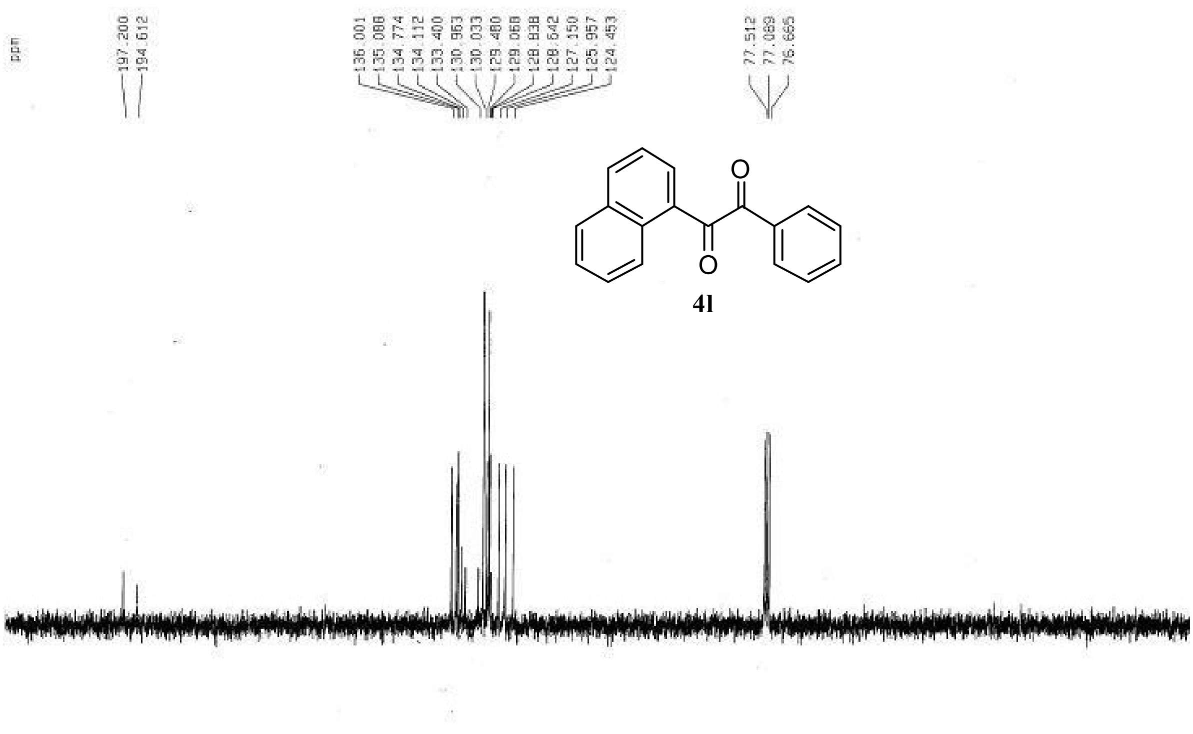

Attached Fig.56 ^{13}C NMR spectrum of 1-(naphthalen-1-yl)-2-phenylethane-1,2-dione recorded in $CDCl_3$

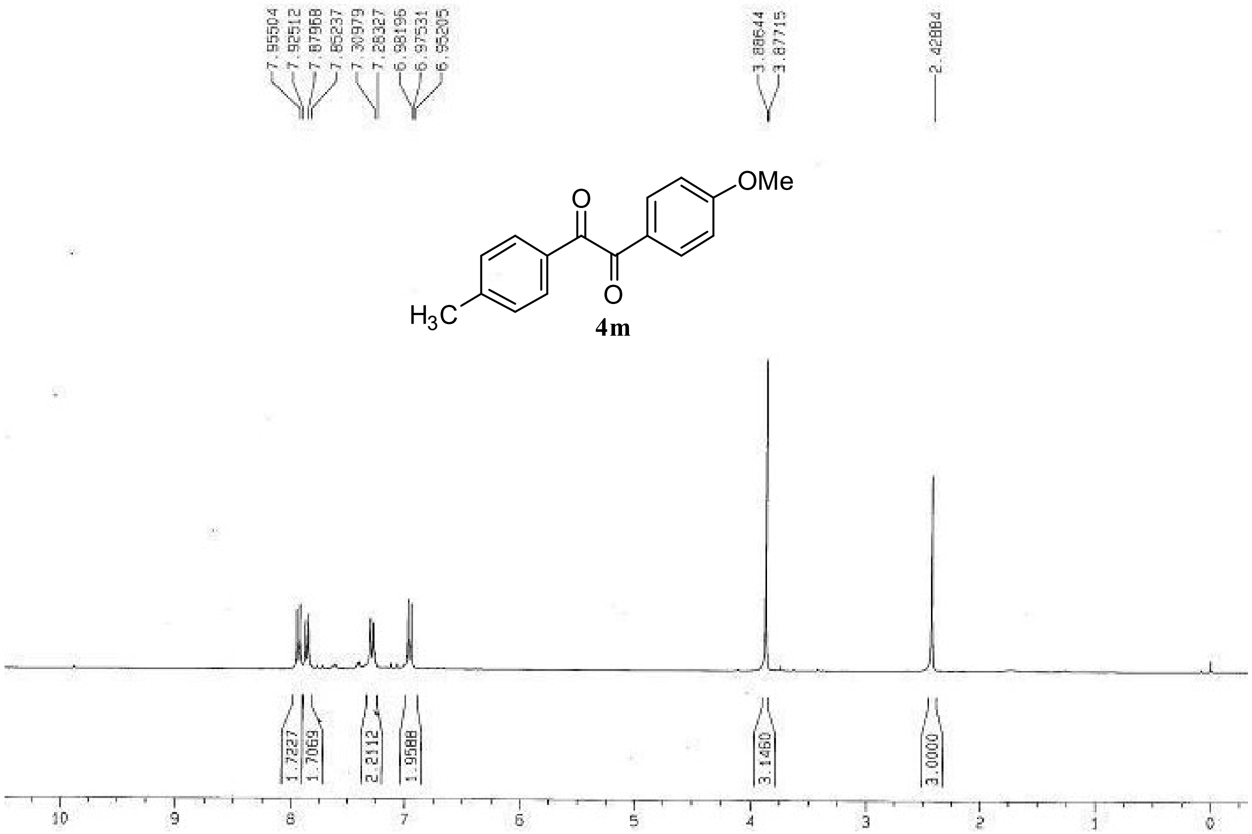

Attached Fig.57 ^{1}H NMR spectrum of 1-(4-methoxyphenyl)-2-(p-tolyl)ethane-1,2-dione recorded in $CDCl_3$

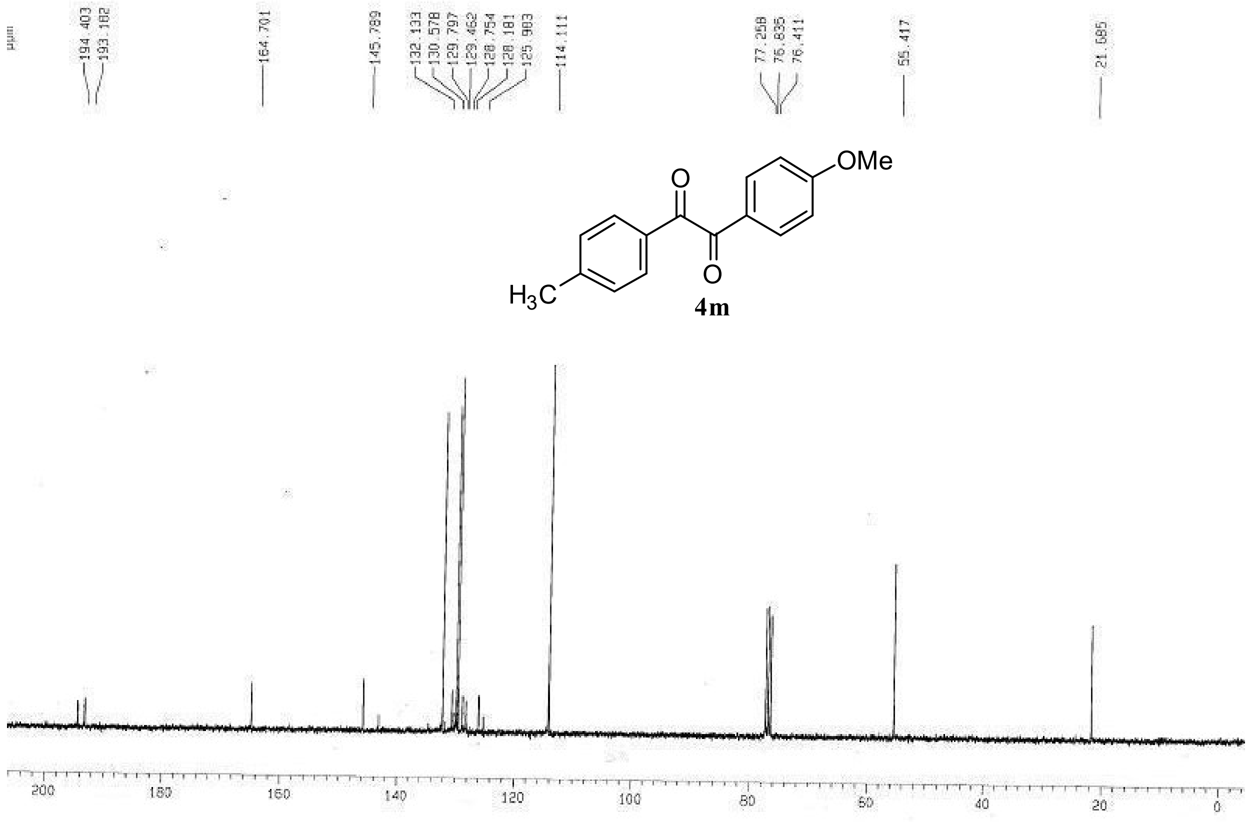

Attached Fig.58 ^{13}C NMR spectrum of 1-(4-methoxyphenyl)-2-(p-tolyl)ethane-1,2-dione recorded in $CDCl_3$

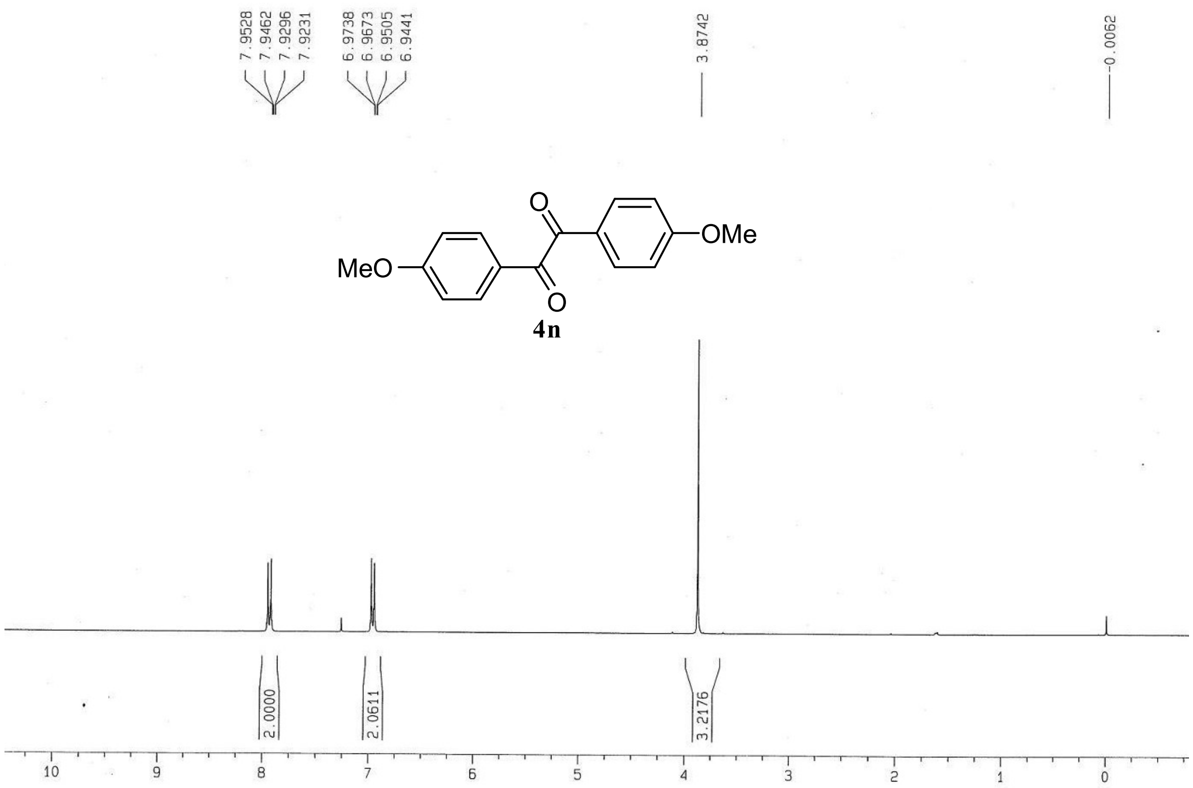

Attached Fig.59 ^{1}H NMR spectrum of 1,2-bis(4-methoxyphenyl)ethane-1,2-dione recorded in $CDCl_3$

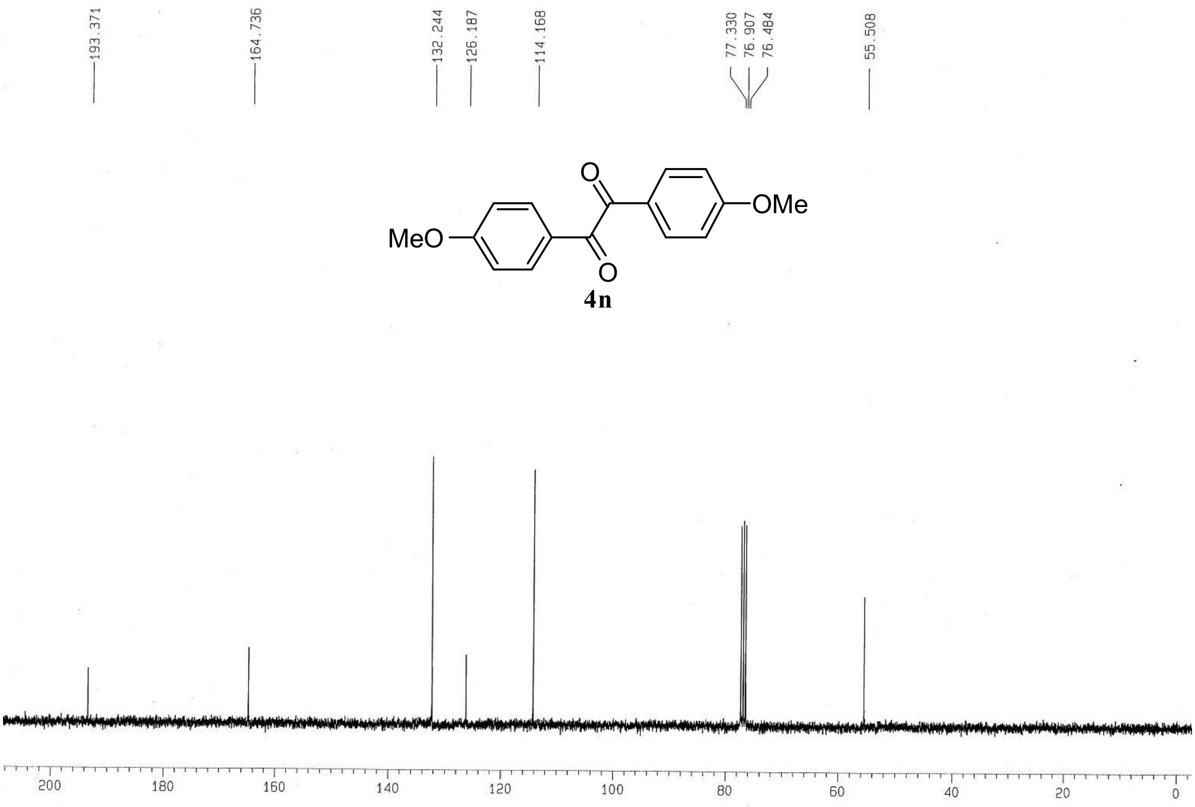

Attached Fig.60 ^{13}C NMR spectrum of 1,2-bis(4-methoxyphenyl)ethane-1,2-dione recorded in $CDCl_3$

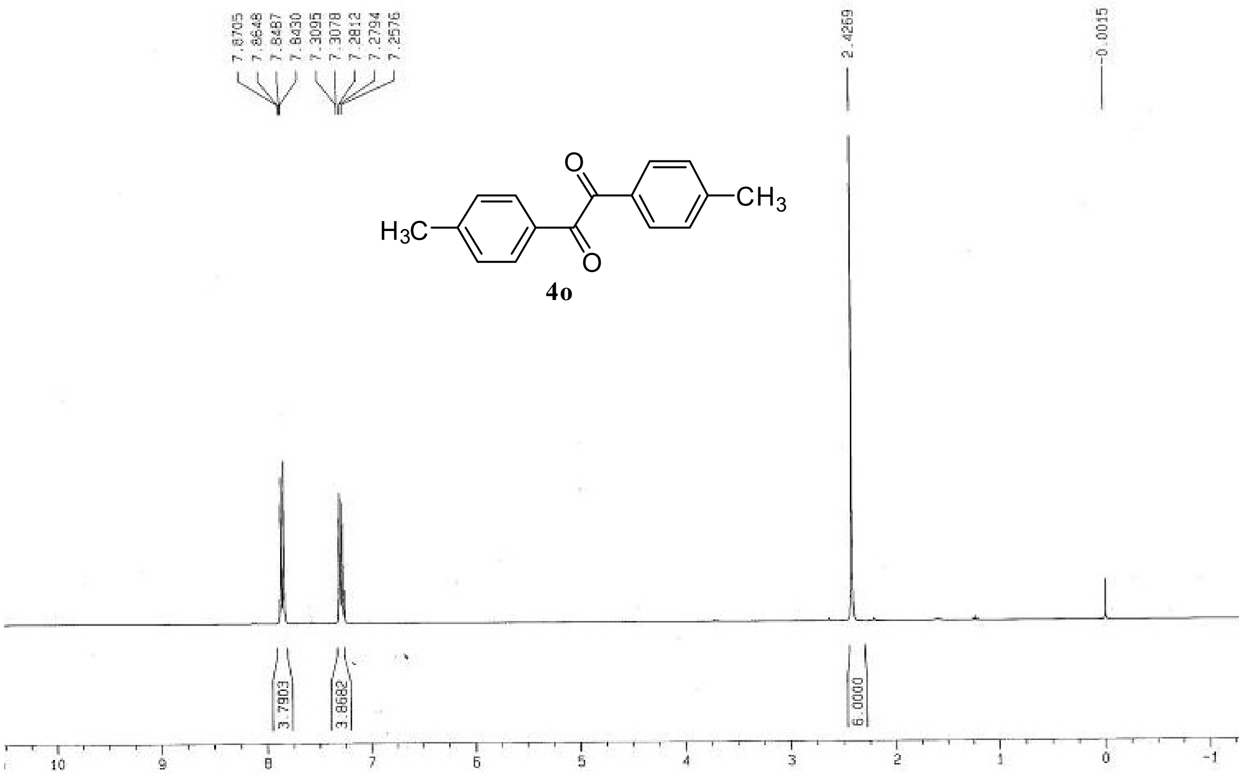

Attached Fig.61 ^{1}H NMR spectrum of 1,2-di-p-tolylethane-1,2-dione recorded in $CDCl_3$

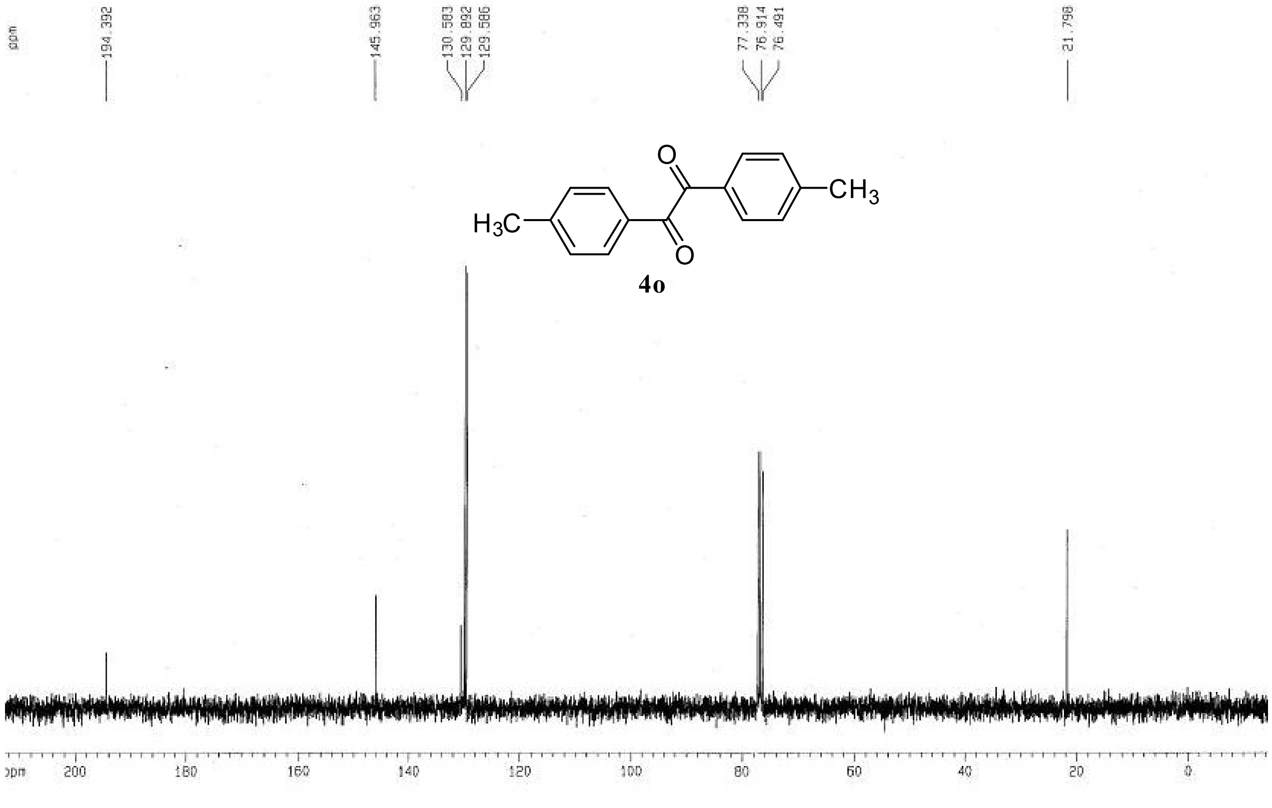

Attached Fig.62 ^{13}C NMR spectrum of 1,2-di-p-tolylethane-1,2-dione recorded in $CDCl_3$
